# Factors affecting lifetime reproduction, long-term territory-specific reproduction, and estimation of habitat quality in northern goshawks

**DOI:** 10.1371/journal.pone.0215841

**Published:** 2019-05-22

**Authors:** Richard T. Reynolds, Jeffrey S. Lambert, Shannon L. Kay, Jamie S. Sanderlin, Benjamin J. Bird

**Affiliations:** 1 Rocky Mountain Research Station, United States Forest Service, Fort Collins, Colorado, United States of America; 2 Rocky Mountain Research Station, United States Forest Service, Flagstaff, Arizona, United States of America; University of Lleida, SPAIN

## Abstract

One measure of habitat quality is a species’ demographic performance in a habitat and the gold standard metric of performance is reproduction. Such a measure, however, may be misleading if individual quality is a fitness determinant. We report on factors affecting lifetime reproduction (LR), the total number of lifetime fledglings produced by an individual, and long-term territory-specific reproduction in a multi-generational study of northern goshawks (*Accipiter gentilis*). LR increased with longer lifespans and more breeding attempts and was strongly correlated with the number of recruits in two filial generations indicating that LR was a good fitness predictor. Extensive differences in LR attested to heterogeneity in individual quality, a requisite for the ideal pre-emptive distribution model (IPD) of habitat settling wherein high quality individuals get the best habitats forcing lower quality individuals into poorer habitats with lower reproduction. In response to 7‒9-year prey abundance cycles, annual frequency of territory occupancy by breeders was highly variable and low overall with monotonic increases in vacancies through low prey years. Occupancy of territories by breeders differed from random; some appeared preferred while others were avoided, producing a right-skewed distribution of total territory-specific fledgling production. However, mean fledglings per nest attempt was only slightly lower in less versus more productive territories, and, contrary to IPD predictions of increases in annual territory-specific coefficients of variation (CV) in reproduction as breeder densities increase, the CV of production decreased as density increased. Rather than habitat quality *per se*, conspecific attraction elicited territory selection by prospecting goshawks as 70% of settlers comprised turnovers on territories, resulting in occupancy continuity and increased territory-specific reproduction. Top-producing territories had as few as 2 long-lived (high LR) and up to 6 short-lived (low LR) sequential breeders. While individual quality appeared to effect territory-specific heterogeneity in reproductive performance, our data suggests that differences in individual quality may be washed-out by a random settling of prospectors in response to conspecific attraction.

## Introduction

Lifetime reproduction (LR) reveals the full extent of variation in fitness potential among individuals, and differences among individuals are the sources of variation on which natural selection works [1, 2]. Common to many studies of LR in birds are extensive among-individual variation in LR and strong correlations between LR and lifespan (longevity) and number of breeding attempts [3, 4]. Other influencing factors include phenotype, habitat composition and structure, food abundance, weather, interspecific competition, weather during different stages of individual life histories, mate quality, predation, population dynamics, and individual covariates such as body size and condition [3, 5–12]. Estimates of variance in LR require data from complete life cycles of individuals [2] but the propensity of juveniles in many species to disperse from a study area make LR data difficult to obtain. Despite this, several studies in which locally-born individuals recruited as breeders into a study population showed that lifetime fledgling production and subsequent numbers of recruits were correlated, indicating that lifetime production of fledglings is a good predictor of fitness [13–15]. Studies of rate-sensitive fitness metrics report that early breeding in life should be favored by selection because starting to breed early can improve an individual’s LR by increasing the number of breeding attempts [1, 16, 17] and changes in reproductive rates are most pronounced in the early years [3, 18, 19]. On the other hand, delayed reproduction may be favored if costs of early reproduction (i.e., reduced survival, lowered future reproduction, and somatic maintenance) outweigh the benefits [20–22]. Alternatively, focusing on the entire lifespans is fundamental for understanding a species’ life history, population ecology, and trade-offs among life history traits. For example, longer lifespans allow more reproductive attempts and increased chances of reproducing during periods of favorable environmental conditions [23–25].

Age at first breeding in many raptors is density-dependent and manifests as increased proportions of young breeders when a population of breeders falls below habitat saturation (i.e., due to high adult mortality) [26–28]. In northern goshawks (*Accipiter gentilis*; here after goshawk), breeding by both sexes <2-years of age occurs occasionally in stable populations but up to 30% in expanding populations of breeders (i.e., due to sudden increases in food resources) [29–31]. Age of first breeding had significant effects on LR of female goshawks in Germany that started breeding at age 1-year. These young females had significantly lower LR than females that delayed breeding until age 3-years, but interestingly there were no differences in breeding lifespans of females that started breeding early or delayed until age 3-years [19]. Higher costs of reproduction by young, inexperienced males vs. females in highly size-dimorphic raptors (males smaller than females) with strongly divergent breeding sex roles (i.e., goshawks) whereby males maintain territories and provision their families with food through the breeding season while females incubate and brood at nests, may be why young females are reported breeding more frequently than young males [32–34]. Age-specific variation in mean number of fledglings per breeding attempt in European goshawks followed the general pattern of age-related reproduction in many birds [35]—a concave curve showing initial increases with age, a peak at mid-age (6- to 7-years-old in goshawks), and a decline in old age [18, 19, 33, 36]. Whether age-specific reproduction follows the concave pattern, whether young breeders produce fewer fledgling in their initial attempt, and whether early breeding affects breeding lifespans and LR in goshawks in America is poorly known.

Because age-specific fledgling production in birds initially improves with increasing age, levels off, and declines in old age, it is often assumed that there is an advantage for both juveniles and adults to pair with adults. In studies where only two age classes of raptors (those in juvenal plumage and those in adult plumage) were considered, there were typically fewer juvenile/adult pairing and more juvenile/juvenile and adult/adult pairing than expected by chance [37–39]. However, when actual ages of adult/adult pairs were considered, there was no tendency for hawks of similar age to pair [38, 39]. In fact, in certain years, pairing in the two age groups in sparrowhawks (*A*. *nisus*) was non-random while in other years pairing was nearly random, differences due perhaps to yearly differences in the available adults [37]. Because many juvenile raptors molt into adult plumage in their second year, is important to recognize that strong mate fidelity may affect assessments of mate choices and that assessments should therefore focus on the actual ages of individuals in the year of pairing. Little variation in the age at first breeding, high breeder survival, and turnover rate can affect appraisals of age-based assortative mating [39–41]. In addition to age, variations in individual body mass (physiological condition) or structural body size (ensemble of morphometrics) may provide cues to the “quality” of potential mates and several authors documented mass- or size-based non-random pairing in raptors [11, 12, 38, 42] while others were unable to do so [43–45]. If, in species with strong territory fidelity, individuals preferentially pair with high quality territory owners, then these individuals choose a territory by default. Of course, a territory owner’s condition might be attributable to its territory quality, making the disentanglement of individual and territory quality problematic [11].

Habitat quality in birds is often attributed to vegetation composition and structure around nests because vegetation characteristics can influence prey abundance and availability [46–48], predation [49–51], competition [52–54], and environmental conditions [55]. Because each of these can affect an individual’s survival (lifespan), breeding attempts, and LR [19, 56, 57], an individual’s reproductive performance in a habitat is a frequently used as a metric of habitat quality [58]. This is because individuals are assumed to preferentially settle into habitats that confer survival and breeding success and there is then an expectation of a congruence between an individual’s fitness and its evolved habitat preferences [59–61]. Understanding the adaptive significance of habitat use in heterogeneous landscapes requires a demonstration of choice and a determination of the fitness consequences of choices [62]. Empiricists often use proxies of fitness such as survival, breeding phenology, brood size, and breeding success, but proxies are seldom shown to be correlated with numbers of fledglings recruited as breeders‒the measure of contributions to future gene pools. Also, there is a lack of consensus on how to measure habitat quality because indices of vegetation structure or productivity have uncertain links to individual fitness and because studies of differences in reproduction among habitats differing in vegetation traits often fail to separate the effects of individual quality (i.e., fitness; the genetic contribution to future generations) from the quality of occupied habitats [24, 58, 63].

Territory-specific reproductive output by goshawks is typically highly variable; some territories are occupied frequently by breeders and produce many fledglings while others were occupied inconsistently and produced few fledglings [64–66]. While variation in territory occupancy and reproduction in birds is typically attributed to variation in habitat quality, it has been argued that some of this variation may be attributable to variations in individual genetic and learned characteristics such as abilities to contest territories and to find and utilize resources through site familiarity about the physical and biotic features of the habitat [63, 67–69]. Based on this argument, several authors suggested that goshawks distribute themselves among habitats in heterogeneous landscapes according to the ideal pre-emptive distribution (IDP) model [70]. The IPD model predicts that the highest quality goshawks acquire the best territories thereby forcing lower quality individuals into poorer territories where reproduction is lower [66, 71, 72]. Thus, expectations under the IPD model are: (1) an among-year non-random occupation of territories, (2) an increase in occupancy of infrequently-used (low quality) territories as a population grows, (3) less variability among-year fledgling production in high quality vs. low quality territories, and (4) more frequent occupancy of low quality territories by young sub-adult individuals [37, 73, 74]. On the other hand, if habitat settling in goshawks follows the IPD model, then individual quality may exaggerate, moderate, or offset the effects of habitat quality, potentially confounding the study of habitat quality [24]. Determining the relationship between fitness and individual versus territory quality is likely to be problematic in long-term multi-generational studies where territories are occupied by multiple (sequential) breeders with variable lifespans, and fledgling production, especially if settling into territories is not “ideal” (individuals do not settle in the best available territories) in the first place. Furthermore, in species with strong territory and mate fidelity, there are likely to be correlations among reproductively-based quality-rankings of territories and/or mates with lifespan, number of breeding attempts, and number mates, especially when increased lifespan results in more breeding attempts and sequential mates. Alternatively, Sergio et al. [24] found that over the long term the effect of parental quality seemed to wash out in black kites (*Milvus migrans*) and that territory quality might then be judged solely on total fledgling production.

Here we report on lifespan, age at first breeding, breeding lifespan, number of breeding attempts, age-specific reproduction, mate choice, and morphological (e.g., body mass, tarsom, wing and tail length) and environmental factors (e.g., breeding density, numbers of mates, and quality ranks of mates and territories) that affect individual LR and recruit production. We also report on how territory-specific differences in (1) years of occupancy by breeders, (2) numbers of unique breeders and mates, and (3) long-term fledgling production affects the study of territory (habitat) quality in a 20-year (1991‒2010) multiple-generational, longitudinal study of male and female goshawks in Arizona, USA. Our aims were to document the extent of variation in LR among goshawks, identify individual and environmental correlates of LR, determine the extent to which LR was a predictor of fitness (genetic contribution to the next generation), and identify factors that could potentially affect a future study of habitat quality based on relationships between the territory-specific demographic performance of individual goshawks and the composition and structure of their forest habitats.

## Methods

### Study area

The study area (1,728 km^2^) was all of the Kaibab Plateau above 2,182 meters above sea level (m.a.s.l.) in northern Arizona, USA (36°26′16′′N, 112°11′55′′W). The Kaibab Plateau is composed of nearly continuous forests of pure ponderosa pine (*Pinus ponderosa*) between ~ 2,075‒2,450 m.a.s.l., a dry mixed-conifer forest comprised of ponderosa pine, Douglas-fir (*Pseudotsuga menziesii*), white fir (*Abies concolor*), blue spruce (*Picea pungens*), and quaking aspen (*Populus tremuloides*)] between 2,045‒2,650 m.a.s.l., and a wet mixed-conifer forest comprised of Engelmann spruce (*P*. *englemannii*), subalpine fir (*A*. *lasiocarpa*), blue spruce, white fir, Douglas-fir, quaking aspen, and ponderosa pine above 2,600 m.a.s.l. Pinyon (*Pinus edulis*)-juniper (*Juniperus* spp.) woodlands occurred below the study area between 1,830‒2,075 m.a.s.l., and a shrub-steppe plain occurred below 1,830 m.a.s.l. [75, 76]. With the exception of several narrow (<1 km) meadows and areas burned by high-severity wildfire, forests on the study area were contiguous [77]. The southern one-third of the study area included the Grand Canyon National Park-North Rim (GCNP), and the northern two-thirds included the Kaibab National Forest (KNF). Forests on the Kaibab Plateau are isolated from other forests by 80 to 250 km of shrub-steppe plain [78]. For detailed descriptions of the study area see [78, 79].

#### Field methods and background

We monitored territory occupancy and reproduction of both male and female goshawks from April through September on a maximum of 125 territories from 1991‒2010 [77, 78]. Northern goshawks are long-lived, monogamous, and territorial forest-dwelling *Accipiter* with high mate and territory fidelity [32, 34, 80]. Active nests and territories were identified when a nest was found with an adult in incubation or brooding postures or if eggs or nestlings were observed. Breeding adults were captured with dho-gaza nets in their nest areas using a live, great horned owl (*Bubo virginianus*) lure from 10 days after egg-hatch to 10 days post-fledging [81]. Breeding adults were initially sexed based on behavior at nests and was confirmed by measures taken when captured on body mass (measured to nearest gram with 1kg and 2kg spring scales), tarsus-metatarsus (tarsom) length, toe-pad length (maximally-stretched distance between the junction of the toe-pad with the hallux talon and junction of the toe-pad with the third digit talon [82]), wing cord (unflattened), and tail length measured to the nearest mm. In years when breeders could not be trapped or resighted, sex determination was as based on behavior at nests. All goshawks received a USGS leg band and a colored aluminum band with unique alpha-numeric codes that were readable from 80 m with 40‒60× telescopes [81]. If a reading of a code was ambiguous (i.e., due to wear), hawks were recaptured, identified by their USGS band, and given a new color band. Use of two bands showed no cases of band loss among resighted or recaptured individuals over the 20 years. Annual field efforts of crews comprised of 15–23 persons were focused on determining territory occupancy (finding nests), visiting active (eggs laid) nests, banding and measuring nestlings, and capturing, measuring, banding, and resighting breeding goshawks. Resighting of banded individuals showed that breeders had strong annual fidelity to territories [78].

Active nests were visited weekly to determine their status, count young, and estimate the timing and causes of nest failures. Nestlings were banded in the 10 days before fledgling. Number of young produced per breeding attempt was taken as the count of nestlings at banding (20‒30 days of age) or, uncommonly, counts of young in nest areas within 10 days post-fledgling if nestlings were not banded [77]. Brood sizes ranged from 1–4, mean annual nest failure rate (fledged no young) was 0.23 (range = 0.12–0.48), and mean annual brood size of successful nests (fledged ≥1 young) was 2.0 fledglings (range = 1.5–2.5 fledglings) [77]. Due to pronounced reversed size dimorphism in goshawks (females mass 1.4 times larger than males), nestlings can be reliably sexed at banding on the basis of morphological measurements, including body mass, tarsus-metatarsus length, and toe-pad length [34, 81, 83]. Our procedure misclassified only 2 of 104 (1.9%) banded nestlings that were subsequently retrapped or resighted as breeders; both had been classified initially as females but were determined to be males on recapture. We were unable to band all nestlings at some nests because of unsafe tree climbing conditions (e.g., snags), late discovery of nests (e.g., at or after fledgling), or logistical constraints in years with many breeders. Nonetheless, fledglings produced at all unclimbed nests were tallied [77]. Individuals were tallied as recruits to the breeding population when they were first trapped or resighted (if banded as nestlings) in a nest area when they were discovered incubating, brooding, or feeding fledglings. Local (*in situ*) recruitment is defined as recruitment of locally-born and banded nestlings into the local breeding population. Immigrant recruitment was estimated at 54% of recruits [75].

Surveyed portions of our study area were saturated with territories, which we defined as an exclusively-used, circular areas centered on nests (if only 1 nest was known in a territory) or the geographic center between 2 or more alternate nests weighted by the number of times each was used by the hawks [78]. Territory size (11.3 km^2^) was estimated as a radius equal to half the mean distance (3.8 km ±0.08 km, range = 1.2–8.4 km, *n* = 588 first-order neighbor distances) among first-order neighboring pairs. Dividing the total study area (1,728 km^2^) by 11.3 km^2^ resulted in an estimated total of 144 territories in the study area. Thus, our sample of 125 monitored territories comprised ~ 87% of potential total territories [77]. The annual frequency of breeding on 121 territories with ≥9 years of monitoring was highly variable, ranging between 8–86% of territories with breeders ($\bar{x}=40\%$) [77]. Variation in the proportion of territories with eggs tracked annual variation in prey abundance in response to variations in pulses of primary forest productivity (with 0–2-year lags) driven by El Niño-Southern Oscillation (ENSO) precipitation at a periodicity of 3–5 wet followed by 3–5 dry years [84–86]. Primary productivity of overstory and understory plants cascaded up through primary and secondary consumers resulting in annual monotonic increases (or decreases) in bird and mammal prey abundance with successive wet (or dry) years [77]. We ranked a year’s quality for breeding based on the proportion of territories occupied by breeders in that year; in good breeding years, more territories had breeding hawks, brood sizes were larger, and fewer nests failed [77].

Unless banded as nestlings (ages known), all breeding individuals at first capture (typically in June–July) were assigned to one of 3 age-classes based on plumage and eye color, where “age” refers to full years since birth. A 2-year-old sub-adult (a hawk in its 3^rd^ year) had many juvenal feathers mixed with adult plumage, yellow-to-orange eyes; a 3-year-old sub-adult (in its 4^th^ year) had predominant adult plumage, scattered juvenal feathers, upper breast with coarse streaking and barring, orange eyes), and a ≥4 year-old adult (in its ≥5^th^ year) had full adult plumage, breast with fine streaking throughout, orange-red to red eyes; hereafter a “≥4-year-old”). Plumage characteristics used to age 2- and 3-year-old unbanded hawks matched the plumages of individuals banded as nestlings at their first capture as 2- and 3-year-olds breeders. Minimum age at first breeding for banded recruits was 2-years [77].

#### Ethics statement and animal welfare

Capturing and banding of goshawks were conducted under United States Fish and Wildlife Service Banding and Auxiliary Marking permit (#21294), United States Geological Service Scientific Collecting permit (#MB044583-0), Arizona Fish and Game Department Scientific Collecting permit (#SP708255), Grand Canyon National Park Scientific Research and Collecting permit (#GRCA-2014-SCI-0025), and Colorado State University Animal Care and Use Committee permit (#05-086A-01). All research activities were consistent with American Ornithologists Union guidelines for capturing and handling birds. All authors declare no conflicts of interest.

#### Lifetime reproduction

We report lifespans, age at first breeding, breeding lifespans (years from first to last breeding), number of breeding attempts, and LR for known-age individuals (banded as nestlings or aged as 1- or 2-years-old on sub-adult plumages; hereafter “known-age” hawks). We separately report minimum lifespans (minimum age of ≥4-years-old at first breeding + subsequent years of breeding), numbers of breeding attempts, breeding lifespans, and LR of individuals that were first captured in full adult plumage and assigned ages of ≥4-years-old. In studies of life history characteristics of breeders, individuals breeding in the first year of a study (or in newly discovered territories thereafter) have unknown breeding histories and individuals still alive at the end of a study have unknown future breeding careers. To minimize bias resulting from inclusion of such individuals, we excluded from our sample of hawks all individuals found breeding on territories when the territories were first discovered. For goshawks to be included in our sample, they would have to have been known replacements of prior breeders (i.e., turnovers) on monitored territories. In the years when turnovers occurred, all new recruits were considered to be first time breeders. To exclude individuals potentially alive at the end of the study (2010), we eliminated all hawks that were newly recruited after 2000. This cutoff resulted in the inclusion of new recruits first breeding in cohorts 1992 through 2000, and left only 1 male and 1 female from these cohorts last known to be alive in 2008.

LR was determined for known (banded) individuals only. Male breeders were particularly difficult to trap and resight; a few could not be captured, and others were not captured until their second or third breeding year. Likewise, we were unable to resight some banded males and females in one or more breeding attempts, especially when an attempt failed before a year’s trapping or resighting was completed. We assumed the same male or female was breeding in a missed year (or years) when those years were bracketed by resights of the same individual, an assumption supported by the strong breeder fidelity to territories [78, 87]. Similarly, we inferred the identity of unbanded breeders or breeders with partially read bands (identity uncertain) up to 3 years prior or subsequent to their capture (or conclusive band readings) only if breeding by these individuals was preceded or followed by >3 years of no breeding on their territories based on the assumption that the prior breeder died. Because no hawks changed territories in one year and returned to breed on their original territory the following year, we were confident in identity inferences of hawks missed in a single year when that year was bracketed by breeding of the same individual on a territory. However, given an approximate 4-year mean breeding lifespan (see below), our confidence in inferring the identity of missed breeders declined as numbers of years without resightings increased. In all cases, broods were assigned to the inferred identity of the male and female breeder.

Due to the possibility of breeders immigrating to or emigrating from the Kaibab, ambiguity remains as to whether all breeding attempts by goshawks in our study were documented. However, based on strong goshawk fidelity to breeding territories on the Kaibab and elsewhere [34, 78, 80, 87], we believe that movement of breeders to or from the Kaibab would have been minimal. Furthermore, the breeding dispersals we observed were rarely beyond 5 territories and any immigrating/emigrating breeder would have to cross as much as 250-km of desert scrubland to nest in other forests. Because of our intensive territory monitoring [78], we believe few if any breeding attempts were missed once territories were discovered. Lastly, we assumed that breeders were the parents of all individuals in their broods. Violations of this assumption were likely rare because only 1 of 77 nestlings at 39 goshawk nests on the Kaibab Plateau had a genotype not consistent with both parents [88], suggesting that extra-pair fertilizations (EPF) on the Kaibab were lower than in other raptors (reviewed in [89]). Due to observed high mate fidelity of Kaibab goshawks, reproduction of paired males and females was not entirely independent. However, because breeding lifespan of pair members seldom overlapped completely, we report LR for both sexes. We tested for differences between the distributions of breeding lifespans, breeding attempts, and LR of known-age goshawks and hawks aged ≥4-years-old at first breeding with two-sample Kolmogorov-Smirnov tests. We tested for differences in numbers of lifetime breeding attempts for goshawks that started breeding early versus late in 3‒4-year periods of good breeding versus poor breeding with a Poisson regression model that included an interaction between timing of breeding (early/late) and breeding year quality (good/poor). Periods of good and poor breeding years were defined by the annual proportions of territories occupied by breeders, using 50% as the threshold (i.e., each year in a period of good breeding years had >50% of territories with breeders, and poor years had ≤50% of territories with breeders).

#### Age-specific reproduction

We evaluated age and breeding year effects on nest success (eggs laid, ≥1 young fledged) and fledgling production with generalized additive mixed models (GAMMs). Nest success (success = 1, failure = 0) was examined using a binomial GAMM with a logit-link function. Age-specific reproduction was analyzed separately for known-age (see above) males and females and goshawks aged of ≥4-years-old on their full adult plumage at first breeding. However, to characterize a year effect on reproduction, a GAMM model was fit using a maximal sample size of the combination of known-age and ≥4-year-olds females only. Each GAMM included year and female age as smoothed fixed effects and band ID and territory ID as random effects. Age-specific effects on fledgling production by both sexes were investigated in separate analyses: at the population level, and at the individual level. The population-level analysis included all hawks where the response variable was the number of fledglings produced per year by individuals in each age class, including individuals that bred only once, and failed nests (0 fledglings). The individual-level included only hawks that bred from one to the next year, and also included nest failures. Our intent in the individual-level analyses was to determine whether individual goshawks followed the same pattern of age-specific as individuals in the population-level analyses (*sensu* [18, 33]). At the individual-level, we fit Gaussian GAMMs with identity-link functions since the response variable (change in number of fledglings produced from one to the next breeding attempt) was not constrained to be ≥0. All analyses were conducted in R [90] and the GAMM models were fit using the *gamm* function in the *mgcv* package with cubic regression splines [91, 92]. Autocorrelation plots showed no significant violations of assumptions. We plotted the raw data for age-specific changes in nest success and fledgling production for both the population- and individual-levels of analysis.

We investigated the effects of early breeding experience on future life-history traits by comparing lifespan, breeding lifespan, number of breeding attempts, number of nest failures, and LR of both sexes first breeding first at age 2-years and then at age 3-years to goshawks that delayed first breeding to ≥4-years-old. We then combined 2- and 3-year-old first-time breeders into a single group and compared this group to goshawks first breeding at age ≥4-years. Lifespans and breeding lifespans were fit using Gaussian models with log-link functions, nest failure data were fit using binomial models with logit-link functions, and number of breeding attempts and LR were analyzed using Poisson models with log-link functions. We used Tukey multiple comparison tests to determine significant differences between groups.

#### Mate choice

We investigated mate choice with regard to age-based assortative mating by comparing mate ages at initial pairings for known-age hawks and hawks aged ≥4-years (where appropriate, inclusive of their known-age mates) separately. We tested for correlations between mate ages with the Wilcoxon-Pratt signed rank test, a nonparametric test that accounts for ties (pairs with same age). To test for mean mate age differences among groups of pairs with different age compositions and previous breeding experience, we used ANOVA and Tukey-Kramer [93] multiple comparisons to control for family-wise error rate. We then combined the samples of known-age and ≥4-year-old hawks in an investigation of the effects of varying mate ages on fledgling production in all breeding attempts (initial and all subsequent pairings) with heat maps of the maximum and ranges of fledglings produced in each attempt.

We also explored any evidence of assortative pairing based on mate quality where quality was indexed by body condition (mass) and structural body size (mass, wing cord, tail length, tarsom); both condition and size are metrics frequently used to predict reproductive fitness and mate quality [11, 94]. Because metrics of body size may be less informative singularly than with a multivariate approach, we used principle components analysis (PCA), which summarizes covarying patterns of variation in morphometric data to produce independent composite variables that can be interpreted as size and shape axes [95]. To investigate whether body sizes of mates could predict LR among mates, we first transformed the raw size measurements of mass, wing length, tail length, and tarsom values into *z*-scores by sex to account for sexual dimorphism, and calculated the total number of fledglings produced by each mating pair (*LR*_*pair*_). We then performed a PCA on the size measurements on each sex separately, and took the first PC as a predictor of *LR*_*pair*_. We fit two generalized additive mixed models (GAMMs) to assess whether (1) mass or (2) size (i.e., the first PC) of either males or females was significantly related to *LR*_*pair*_, and included random effects for individual birds to account for repeated observations among individuals. GAMMs were used to assess potential non-linear relationships between size and *LR*_*pair*_.

#### Individual and environmental covariates of LR

Various life-history metrics such as lifespan, age at first breeding, breeding attempts, nest failures, and morphological metrics, such as body size and condition, are frequently used as measures of individual fitness [96, 97]. We used Poisson generalized linear models (GLM) to investigate the effects of 9 explanatory variables for individual goshawks (lifespan, age at first breeding, breeding attempts, nest failures, body size [mass relative to mean mass of all sex-specific mates], tarsus-metatarsus length, wing cord, tail length, and body mass), and 5 environmental explanatory variables (number of mates, proportion of territories with breeding pairs, directional changes in mass of changed mates, territory rank, mate rank) on LR of individual male and female goshawks (variables and acronyms described in Table 1). In all cases, the unit of observation was an individual male or female, and the response variable was the number of young produced in their lifetime. We used morphological measurements that were taken when a goshawk was initially captured as an adult. Measurements included body mass using Pesola scales, caliper-determined length of metatarsus, wing cord (from the bend of as unflattened wing to the tip of the longest primary), and length of central tail feathers. Trapping of breeders, especially males, was a protracted process that required stealth, patience, and expediency. To minimize disturbance in nest areas, we occasionally released difficult-to-capture breeders before morphological measurements were completed. In cases with missed measures, we used measures taken at subsequent recaptures (e.g., next breeding attempt), or, if not recaptured, we used non-parametric (most morphological variables were not normally distributed) single imputation in R [98] with the package *MissForest* [99] to estimate missing morphometrics. We standardized all quantitative variables (mean = 0, SD = 1) and conducted analyses using all explanatory variables for those individuals with complete morphological data.

**Table 1:** Explanatory variables for individual and environmental effects on lifetime reproduction (LR) of northern goshawks in Arizona, USA. Table includes variable name and variable description for generalized linear models.

Variable Name	Description
***Lifespan***	Number of years an individual lived (hatch to disappearance).
***Agefirstbreeding***	An individual’s age at first breeding (eggs laid).
***Breedingattempts***	Total breeding attempts in an individual’s lifespan.
***Avgbrpairs***	Average annual proportion of territories with breeders during an individual’s reproductive years. A measure of quality of year for breeding and an indicator of the density of breeders. Proportion of territories with breeders was calculated as the number of known territories with breeders in a year divided by the number of territories known in the prior year.
***Nummates***	Number of different mates an individual bred with in its lifetime.
***Mateswitch***	Averaged direction of change in a mate’s mass following change of mate. Change values were -1 for new mate smaller than previous mate, 0 for no change in mate mass, 1 for new mate larger than previous mate. For individuals with just one mate, the value was coded as 0.
***Nestfailures***	Frequency of nest failure (eggs laid, no fledglings produced) over lifespan of an individual.
***Avgpermass***	Hawk mass relative to average mate mass (hawk mass/average mass of all of its mates). A measure of the extent of reversed (males smaller than females) size dimorphism. Only mass of individuals taken at first capture was used. Imputed mass values were used for individuals with missing data.
***Avgterrank***	Average rank of territories used by hawks during their reproductive years. Rank determined by rank-ordering territories on final counts of fledglings standardized by number of years each was monitored. The most productive territory received a rank of 1. Territories with the same total fledglings received the same rank.
***Avgmaterank***	Average rank of mates during a hawk’s lifespan. Rank determined by rank-ordering banded males and females separately on total lifetime production of fledglings where a ranking of 1 was the most productive. Mates with the same total fledglings received the same rank.
***Tarsom***	Tarsom length (mm).
***Mass***	Body mass (g).
***TailL***	Tail length (mm).
***WingC***	Wing cord (cm).

All explanatory variables were quantitative, except *mateswitch*. *Mateswitch* had 3 categories (smaller mate as baseline, no change in mate size, and larger mate). We used R package *MuMIn* [100] for model selection based on *AICc* [101], because the Pearson *χ*^2^ goodness-of-fit statistic with our most general GLM indicated no overdispersion for all data sets (male, excluding *tarsom*, *wingC*, *mass*, and *tailL*: $\hat{c}\;<\;1.000$, *χ*^2^ = 36.57, *df* = 64; male, including *tarsom*, *wingC*, *mass*, and *tailL*:$\hat{c}\;<\;1.000$, *χ*^2^ = 27.48, *df* = 50); female, excluding *tarsom*, *wingC*, *mass*, and *tailL*: $\hat{c}\;<\;1.000$, *χ*^2^ = 41.39, *df* = 78; female, including *tarsom*, *wingC*, *mass*, and *tailL*:$\hat{c}\;<\;1.000$, *χ*^2^ = 43.00, *df* = 71). *Avgbrpairs*, the proportion of territories with breeders in a particular year, was included as a measure of a year’s quality for breeding; the greater the proportion of territories with breeders, the better was the breeding year. *Avgbrpairs* was also a measure of density of breeders because, as the proportion of breeding pairs increased, so did the density of breeders. We report model-averaged slope estimates and the relative importance of terms (sum of *AICc* weights over all models including the explanatory variable). We ran correlations (Pearson’s or rank) among all quantitative explanatory variables of LR separately for males and females. Because only *lifespan* had a Pearson correlation >0.7, we excluded *lifespan* from all candidate model sets.

#### Fledgling production and fitness

We evaluated the reliability of fledgling success as a measure of individual fitness by comparing individual fledgling production to the number of fledglings eventually locally recruited. We displayed among-individual variation in LR by rank-ordering (most to least productive) male and female breeders on numbers of fledglings produced and plotting cumulative numbers of fledglings against cumulative numbers of breeders. Because a quasi-Poisson model showed no significant overdispersion, we used a Poisson GLM [98] and package *AER* [102] to examine the relationship between the LR of male and female breeders whose young were banded and the numbers of first (F1) and second generation (F2) recruits they produced (there were too few F3 recruits for modeling). Parameter estimates ±SE are given unless otherwise specified.

#### Territory occupancy and reproduction

We tested if goshawks annually nested preferentially or randomly in 79 territories each monitored at least 18 years in a chi-square goodness-of-fit test (*sensu* [63, 103, 104]). We binned territories into groups of 3 years of occupancy (1‒3, 4‒6, 7‒9, and so on) with the final bin containing the last four years in order to meet assumptions of the test. Additionally, we visually assessed territory preference by plotting overlapping frequency distributions of observed number of years territories were occupied with a random simulation. If no preference (i.e., hawks randomly nested in territories every year) the expectation is that the majority of territories would be occupied for about half the monitoring period (i.e., 10 years). Conversely, if territories were chosen preferentially some would be occupied for a few years only while others (preferred territories) would be occupied through much of the monitoring period. Preferential choice produces convex distributions of occupancy while the absence of preference produces a concave distribution.

Reproductive performance in a habitat is the gold standard metric of habitat quality and long-term total reproduction in a habitat is a function of the number of successful breeding attempts and brood sizes per attempt. We investigated differences in territory-specific mean fledglings produced per breeding attempt and mean long-term total fledgling production in infrequently versus frequently occupied territories in 3 non-overlapping cohorts of territories: 36 territories studied 20 years (1991‒2010); 25 territories studied 19 years (1992‒2010; 1 territory excluded because of loss due to high-severity fire in 2000); and 18 territories studied 18 years (1993‒2010). We used analysis of covariance (ANCOVA) and the *F*-test for evidence of differences in regression slopes among cohorts.

## Results

### Lifespan, breeding attempts, and fledgling production

In our 20-year study we monitored reproduction of 195 male and 250 female goshawks at 846 active (eggs laid) nests on as many as 125 territories (totaling to over 2,112 territory-monitoring years). Breeding occurred on average in only 40% of territories every year, 21% (176/846) of active nests failed and 79% (670) fledged ≥1 young [77]. In cases of partial reads of band codes where resighting was limited to band leg and/or band color, or when nests failed before resighting was completed (see Methods), the identity of breeders was inferred in 154 male and 123 female cases. When resights were missed in one or more successive years on a territory but were bounded by successful resights of the same individual, the identity of a missed breeder was inferred to be the same individual in 28% (43 of 154 inferences) of male cases and 40% (49 of 123) of females cases. For partial or failed resightings in a single year either before or after a breeder was trapped or resighted on a territory and the single year was not bounded by successful resights, the identity of the breeder was inferred to be the same as the trapped or resighted individual in 51% (79 of 154 inferences) for male cases and 49% (60 of 123) for female cases. Similarly, for partial or failed resightings in 2 successive years not bounded by resights, the identity of a breeder was inferred to be the same as the trapped or resighted individual in 18% (27 of 154) male cases and 10% (12 of 123) female cases. For partial or failed resightings in 3 successive years, the identity of a breeder was inferred to be the same as the trapped or resighted individual in 3% (5 of 154) male cases and 2% (2 of 123) female cases. Each of the above unbounded identity inferences were made only if there were breeding gaps of ≥4 successive years in a territory that preceded or followed the inference years. While confidence in these inferences declined with increasing successive years of missed resightings, we nonetheless believe that strong territory fidelity and frequent turnovers of banded hawks following ≥4 breaks in breeding by Kaibab goshawks supported our inferences.

Forty-five male and 58 female goshawks we banded as nestlings recruited as breeders into the local population and were therefore of known-age. In addition to these, a few unbanded goshawks (13 males, 30 females) were aged as 2- or 3-year-olds based on their subadult plumage at first breeding (see Methods). However, the majority of recruits (137 males, 162 females) were unbanded and in full adult plumage at first breeding and could be aged only as ≥4-years at that time. In our analyses of lifespans, breeding lifespans, and LR, we eliminated all goshawks whose reproductive histories were unknown (i.e., those initial breeders in newly discovered territories). This eliminated all breeders in the 1991 cohort and a few others in later cohorts (S1 Fig). To ensure that we included only individuals whose full breeding lifespans were confidently observed, we eliminated all hawks that recruited as breeders after 2000. Our final sample of hawks included 69 males and 95 females, all from the 1992‒2000 cohorts.

**Fig 1:**
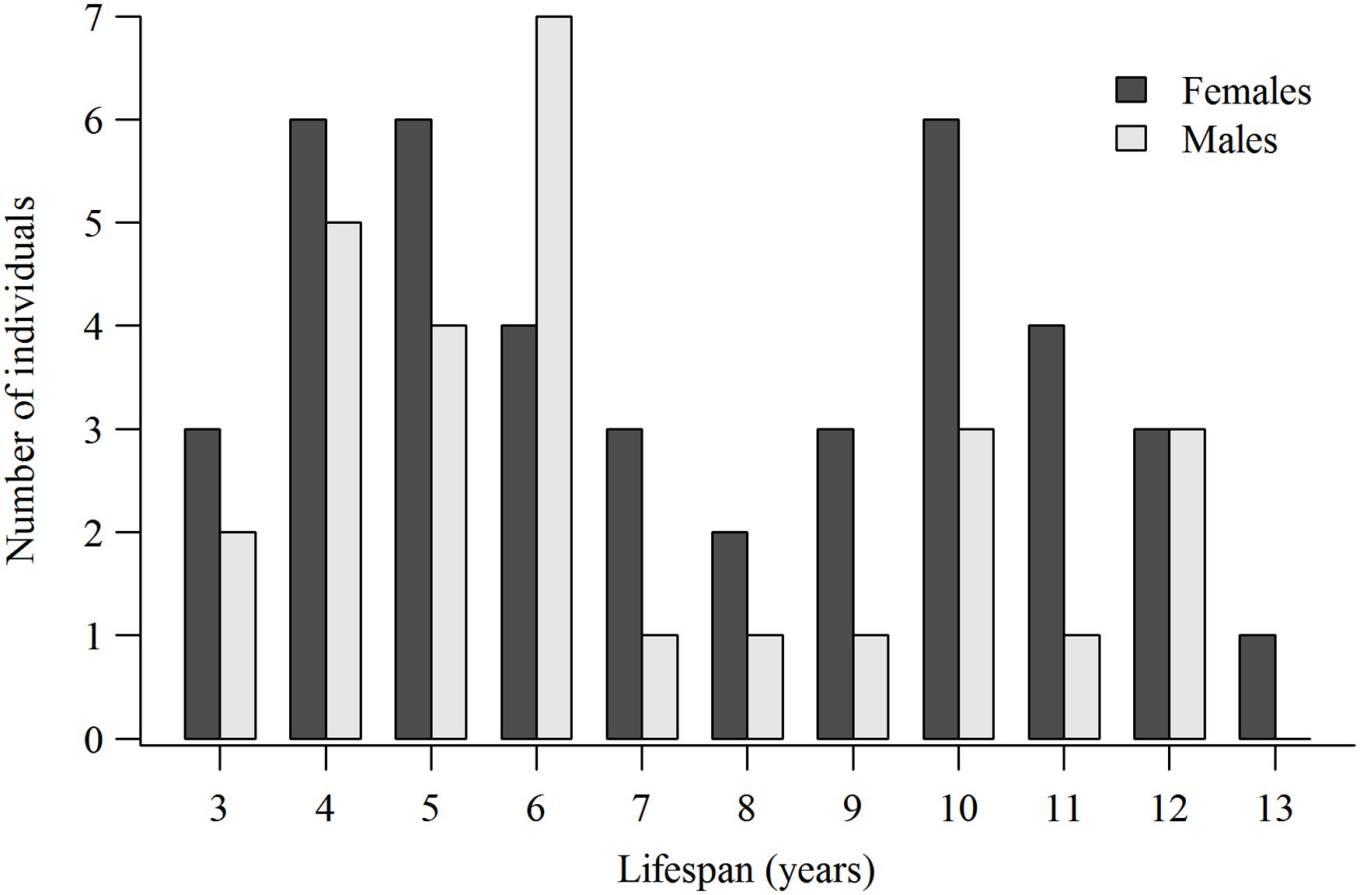
Number of goshawks by lifespan of known-age males and females. Lifespan of 28 male and 41 female northern goshawks of known-age (banded as nestlings or aged 2- or 3-years-old based on plumage at first breeding) northern goshawks first breeding in the 1992‒2000 cohorts of breeders in Arizona, USA, 1991–2010.

We first report lifespans, breeding lifespans, number of breeding attempts, and LR for the 69 (28 males, 41 females) known-age goshawks separately from the 95 (47 males, 48 females) ≥4-years-old hawks. Lifespans of known-age hawks showed multi-modal distributions with a major peak at 6-years and lesser peaks at 10- and 12-years-old in males, and major peaks at 4-, 5-years, and 10-years-old in females (Fig 1). The peaks of older hawks reflect the single (occasionally 2) long-lived individual in each annual cohort (S1 Fig). Except for slightly lower mean LR among known-age males (4.4 fledglings) than known-age females (5.8 fledglings), ages at first breeding, lifespans, breeding lifespans, and number of breeding attempts were similar for both sexes (Table 2). Mean minimum lifespans of ≥4-year-old males (7.8 years) was 1 year longer than lifespans of known-age males (6.8 years), whereas mean female lifespans was only slightly longer for the ≥4-year-old group (8.0 years) than for the known-age group (7.4 years). Interestingly, while mean LR in the 2 groups were the same for females (5.8 fledglings), the ≥4-year-old male mean LR (5.9 fledglings) was more than a fledgling greater than LR of known-age males (4.9 fledglings). Despite these differences, breeding lifespans and number of breeding attempts were nearly identical for both known-age and ≥4-year-old males and females (Table 2). Lower LR of known-age males likely reflected the inclusion of 2- and 3-year-old in this group of males that, while they made similar numbers of breeding attempts, they suffered higher nest failure rates than older breeders (see below). Because the Kolmogorov-Smirnov tests showed no between-group differences in distribution of breeding lifespans, numbers of breeding attempts, and LR (*P* = 0.9934, *P* = 0.9994, *P* = 0.8806, respectively), we combined the 2 groups of hawks with the caveat that we report the minimum lifespans of ≥4-year-old hawks (≥4-years + years observed alive).

**Table 2:** Life history characteristics of breeding goshawks. Mean±SE and median (range) of age at first breeding, lifespan, breeding lifespan (first to last breeding year), number of breeding attempts, and lifetime production (LR) of fledglings of 164 (69 known-age plus 95 ≥4-years-old) male and female northern goshawks first breeding in the 1992–2000 cohorts in Arizona, USA.

	Males		Females	
	Known age (*n* = 28)	≥4-years-old (*n* = 47)	Known age (*n* = 41)	≥4-years-old (*n* = 48)
**Age at first breeding**^1^	2.9±0.27 3 (2–9)		3.2±0.24 3 (2–8)	
**Lifespan**^2^ **(years)**	6.8±0.54 6 (3–12)	≥7.8±0.41 ≥7 (5–16)	7.4±0.47 7 (3–13)	≥8.0±0.39 ≥8 (5–16)
**Breeding lifespan (years)**	3.8±0.48 3 (1–9)	3.9±0.41 3 (1–12)	4.2±0.46 3 (1–11)	4.1±0.39 4 (1–12)
**Breeding attempts**	3.1±0.34 2.5 (1–7)	3.4±0.33 3 (1–11)	3.4±0.35 3 (1–10)	3.4±0.31 3 (1–11)
**Lifetime reproduction**^3^	4.4±0.57 3 (0–11)	5.9±0.63 6 (1–19)	5.8±0.65 5 (0–18)	5.8±0.63 5 (0–23)

^1^Age at first breeding unknown for hawks aged ≥4-yr-old.

^2^Lifespan of hawks aged ≥4-years-old at first breeding shown as a minimum.

^3^Total fledglings produced in lifetimes of individuals (LR).

**Fig 2:**
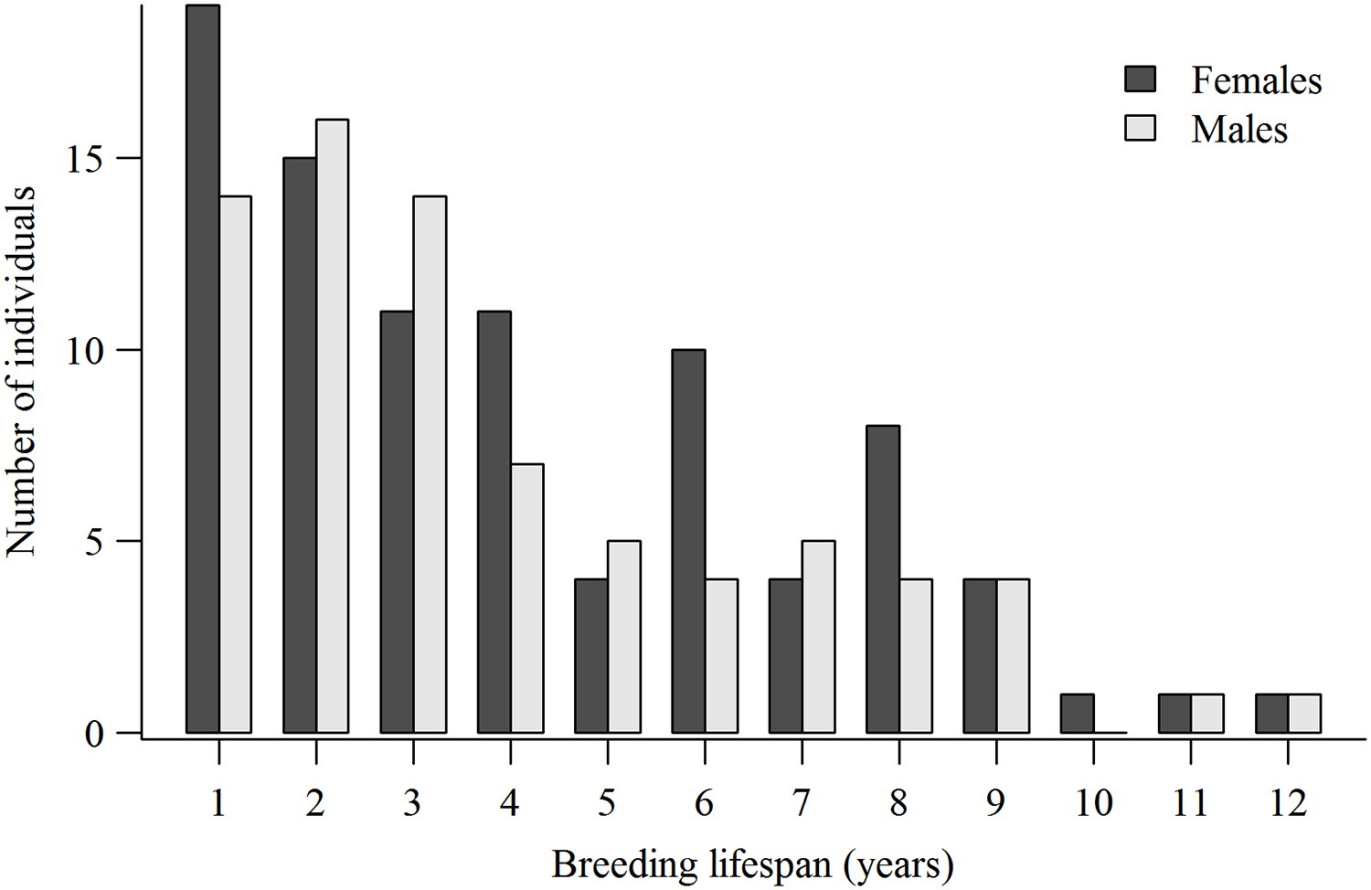
Breeding lifespans (years from first to last breeding) of 75 males and 89 females of combined known-age and ≥4-year-old goshawks first breeding in the 1992‒2000 cohorts in Arizona, USA.

**Fig 3:**
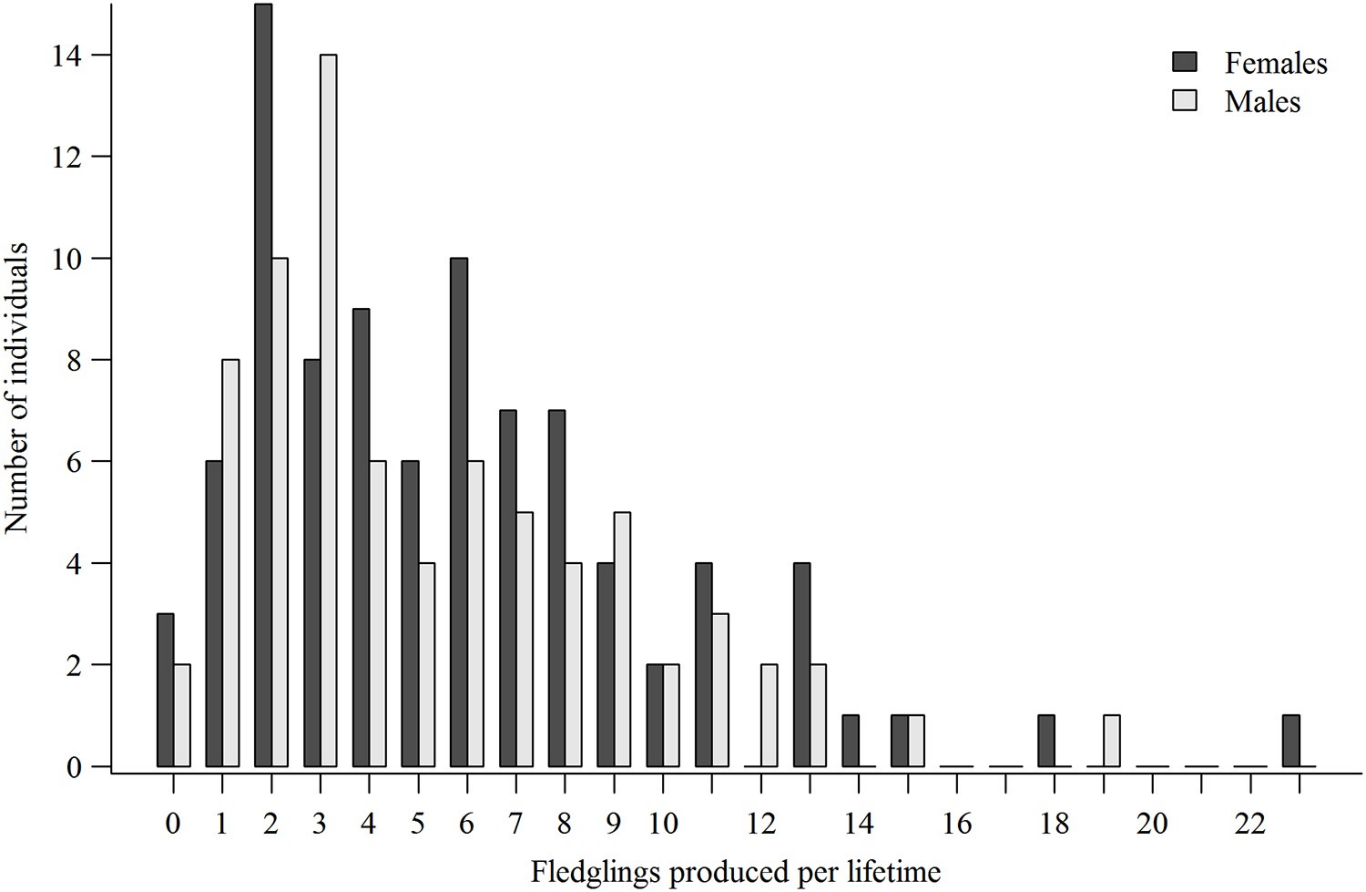
Number of fledglings produced by goshawks during their lifetimes. Individual lifetime reproduction (LR) of 75 males and 89 females of combined known-age and ≥4-year-old goshawks first breeding in the 1992‒2000 cohorts in Arizona, USA.

Plots of breeding lifespans and LR for both sexes of combined known-age and ≥4-year-old breeders showed strongly right-skewed individual variation in fitness potential (Figs 2 and 3). The combined male mean breeding lifespan was 3.9 ±0.31years (median = 3, range = 1–12), mean breeding attempts was 3.3±0.24 (median = 3, range = 1–11 attempts), and mean LR was 5.3 ±0.45 fledglings (median = 4, range = 0–19). For females, the combined mean breeding lifespan was 4.1±0.30 years (median = 3, range = 1–12 years), mean breeding attempts was 3.4 ±0.23 (median = 3, range = 1–11), and mean LR was 5.8 ±0.45 fledglings (median = 5, range = 0–23). Combined mean lifetime productivity peaked at 3 fledglings for males and 2 fledglings for females with approximately 60% of males and 53% of females producing 0‒5 fledglings, and only 15% of males and 16% of females producing 10 or more fledglings.

Pairs of goshawks that started breeding at the beginning of a 3- to 4-year period of good breeding conditions made more breeding attempts ($\bar{x}=3.3$ attempts, $\hat{\lambda }=3.3$) than those starting late in the phase ($\bar{x}=2.9$ attempts, $\hat{\lambda }=2.9$) (see [105]). However, a significant interaction was found (*P* = 0.05) between timing (early/late) and quality (good/poor), where pairs starting to breed in the last year of a poor period made the most lifetime breeding attempts ($\bar{x}=4.1$ attempts, $\hat{\lambda }=4.0$) as breeding by these pairs continued into the best of breeding years. Finally, we note that the 3‒4-year cycles of good and poor breeding conditions may have introduced bias to our estimates of goshawk lifespans as lifespans would have been underestimated for individuals surviving into, but not through, periods of poor breeding conditions due to the low detectability of non-breeders.

### Age at first breeding

**Fig 4:**
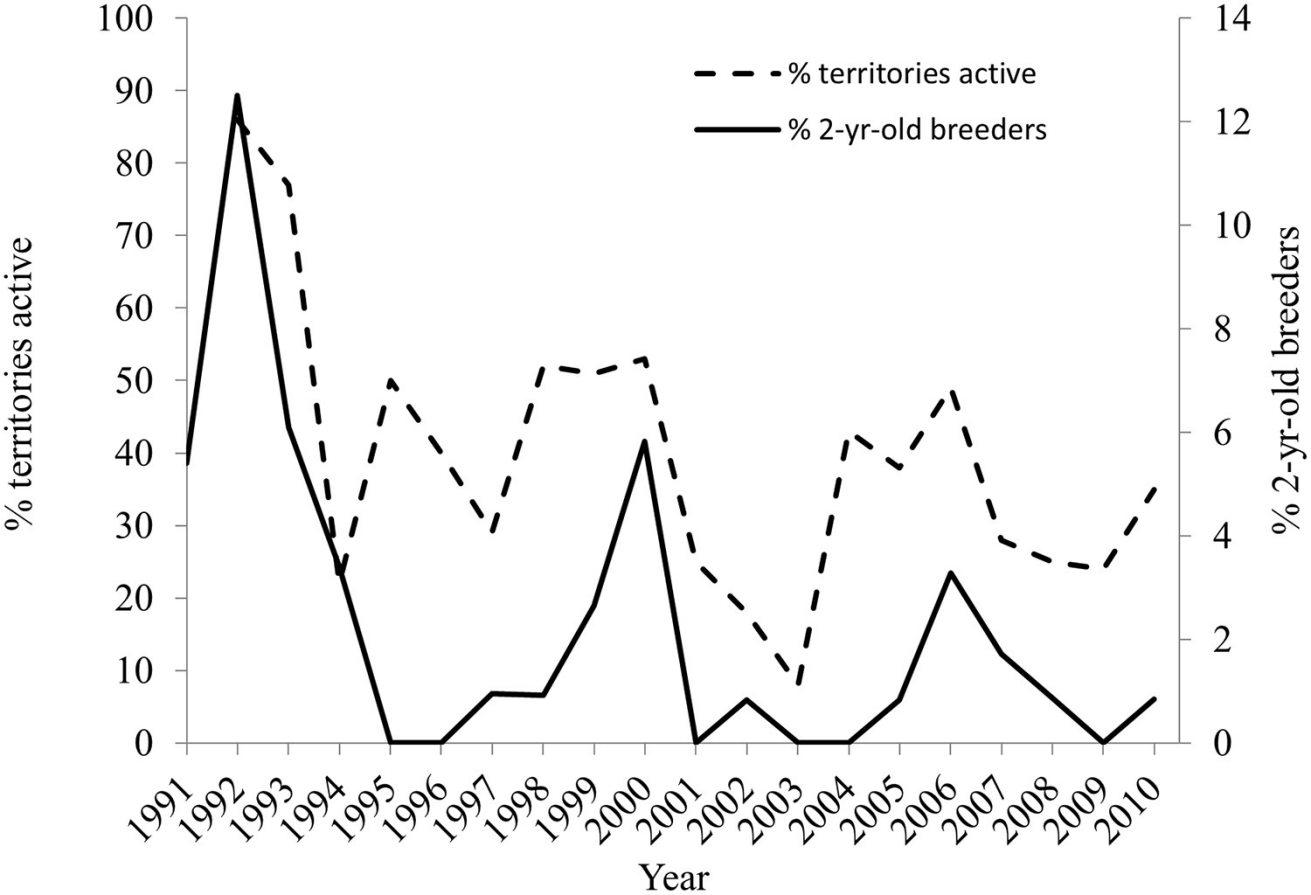
Annual percent of breeders that were 2-years-old was a function of a year’s quality for breeding. Annual percent of breeders that were 2-years-old as a function of the quality of a breeding year (estimated as the percent of territories with egg-laying pairs; [77]) of northern goshawks in Arizona, USA. Monotonic increases in the recruitment of 2-year-old breeders indicated improving breeding conditions and the existence of vacancies on territories following periods of poor breeding.

The minimum age at first breeding for 195 males and 250 females was 2-years and mean age at first breeding by the 58 known-age male and 88 female goshawks was 3.6±0.21-years (median = 3, range = 2–9) for males and 3.6 ±0.18-years (median = 3, range = 2–9) for females (Table 3). Of the 58 males and 88 females of known-age, 77% of the males and 80% of the females first bred at or before age 4-years, and 87% of males and 83% of females bred at least once by age 5-years (Table 3). If goshawks aged ≥4-years-old at first breeding had proportional age distributions as known-age hawks that were 4-years or older at their first breeding, then about 60 (43%) of the 137 ≥4-years-old males would have been 4-years-old, 36 (26%) would have been 5-years, 30 (22%) would have been 6-years-old, and 12 (9%) would have been older than 6-years. For ≥4-years-old females, about 76 (47%) of the 162 females would have been 4-years-old, 15 (9%) would have been 5-years, 35 (22%) would have been 6-years, and 35 (22%) would have been older than 6-years. Given these estimates, the mean ages at first breeding was 5.2-years for the ≥4-year-old males and 5.3-years-old for the females. These means exceeded the mean ages of first breeding by known-age males and females by about 1.6 years and show that, by extension, the actual lifespans of hawks aged ≥4-year-old at first breeding would be greater than their mean minimum lifespans of about 7.6-years (Table 2). The proportion of 2-years-old breeders in the breeding population was highly variable among years and showed monotonic increases with each successive year of improved breeding conditions as more territories were filled by active breeders. On the other hand, the proportions of breeding 2-year-olds declined sharply with the first year of declining conditions (Fig 4). Annual fluctuations in the proportions of 3-year-old first-time breeders were not as extensive as for 2-year-olds and exceeded 3% only in 1998 (9.2%) and 2000 (11.7%).

**Table 3:** Number of individuals by age (number of full years since hatch) at first breeding (%) for 58 male and 88 female northern goshawks of known-age in Arizona, USA, 1991–2010.

	Age										
	1	2	3	4	5	6	7	8	9	10	Mean
**Males**											
age 1st breeding		17 (29)	18 (31)	10 (17)	6 (10)	5(9)	0	0	2 (3)	0	3.6
**Females**											
age 1st breeding		25 (28)	31 (35)	15 (17)	3 (3)	7 (8)	4 (5)	2 (2)	1 (1)	0	3.6

### Age-specific reproduction

#### Nest success

**Fig 5:**
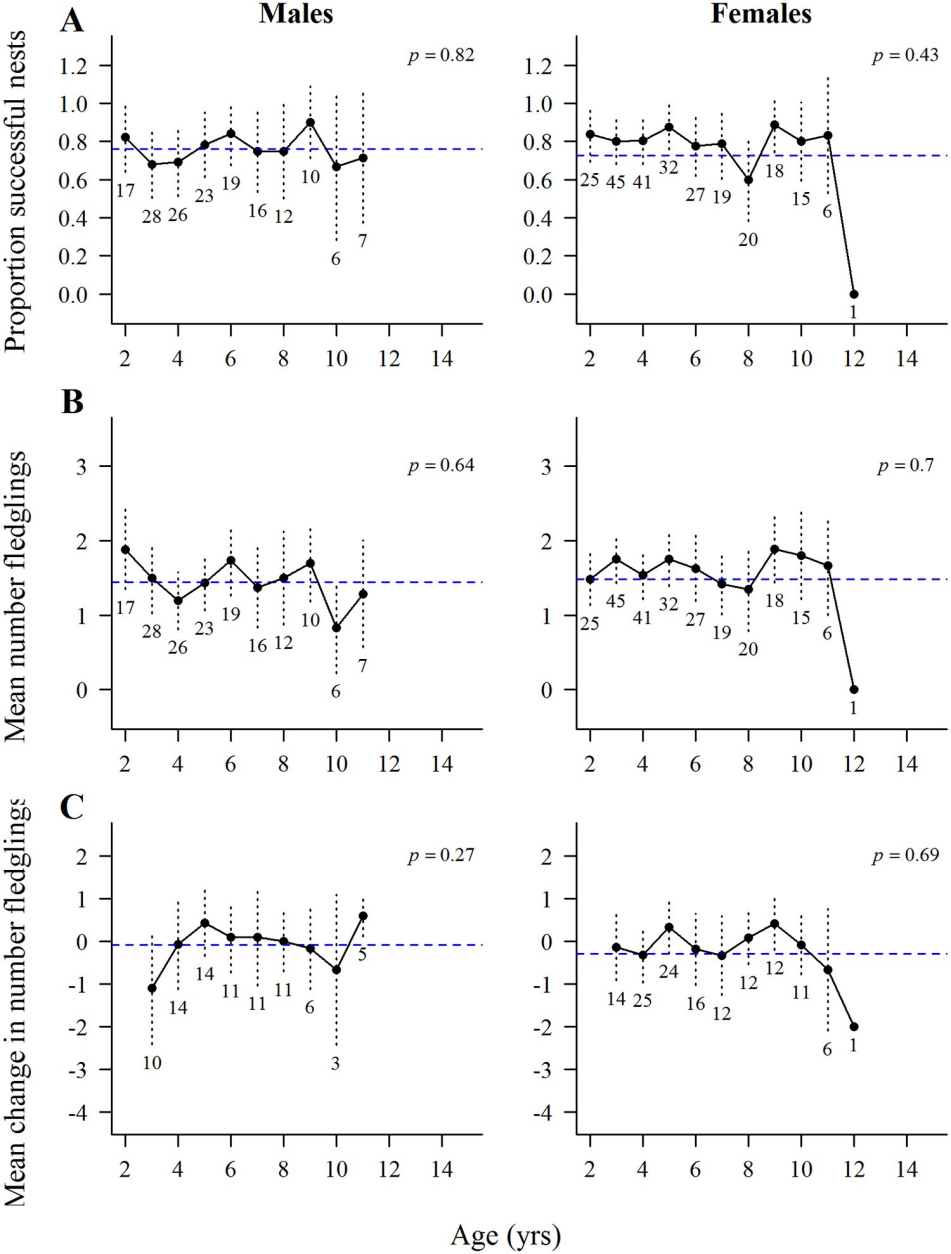
Age-specific reproduction by known-age goshawks. Means, ±2 standard errors, and overall mean values (horizontal dashed line) of the raw data from GAMM analyses of age-specific reproduction by known-age male and female northern goshawks in Arizona, USA. Numbers below standard error bars indicate the number of males and females in each age group. (A) nest success (fledged ≥1 young) as a function of age of 164 breeding attempts by 58 males and 249 attempts by 88 females, (B) fledgling production as a function of age in 164 breeding attempts by 58 males and in 249 attempts by 88 females (population-level analyses), and (C) changes in numbers of fledglings produced in 85 breeding attempts by 40 males and 133 breeding attempts by 55 females that bred in one and the next year (individual-level analyses).

**Fig 6:**
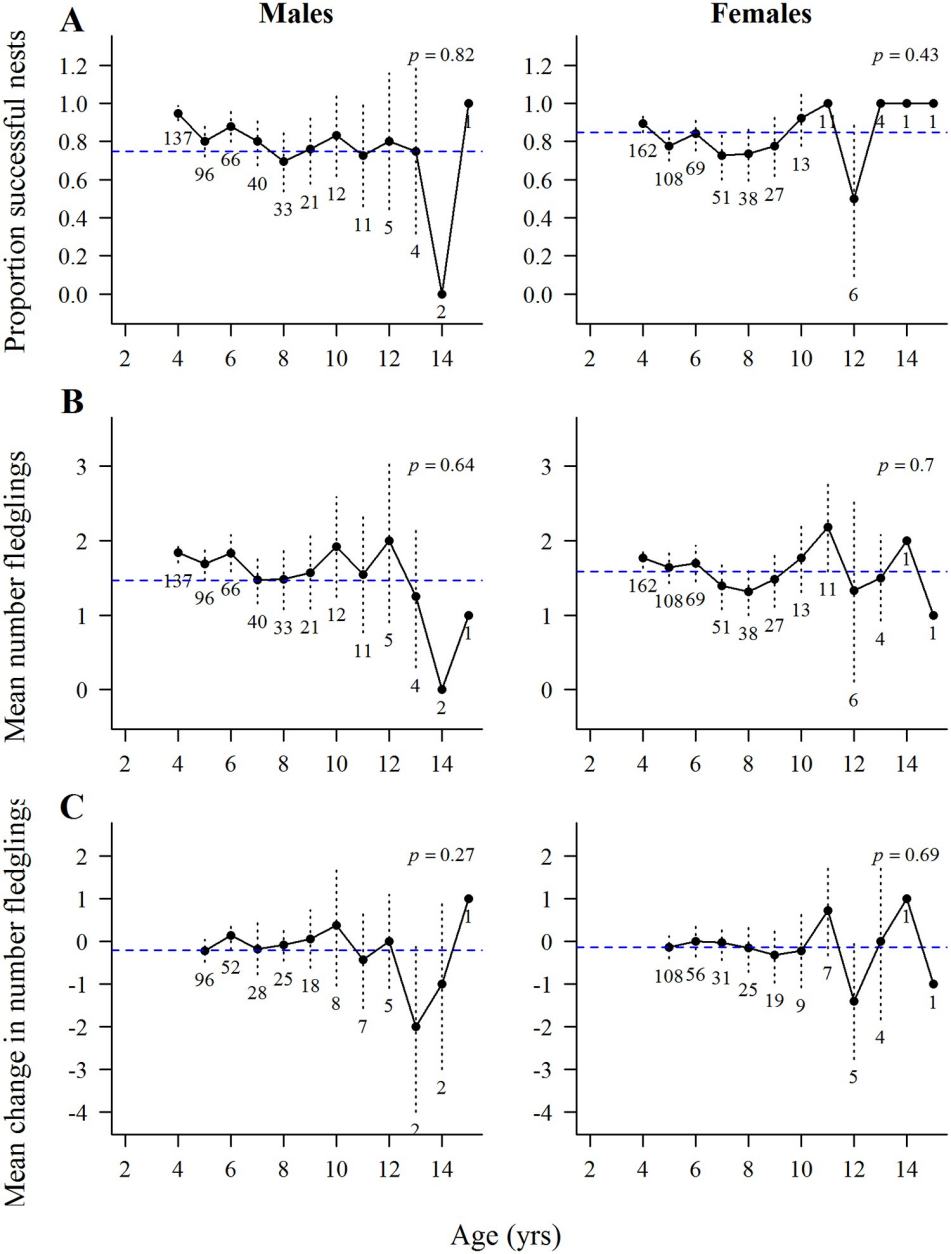
Age-specific reproduction by goshawks aged ≥4-years-old at first breeding. Means, ±2 standard errors, and overall mean values (horizontal dashed line) of raw data from the GAMM analyses of age-specific reproduction by male and female northern goshawks assigned a minimum age of ≥4-years old at their first breeding attempt in Arizona, USA. Numbers below standard error bars indicate the number of males and females in each age group. (A) nest success (fledged ≥1 young) as a function of age in 428 breeding attempts by 137 males and 491 attempts by 162 females, (B) fledgling production as a function of age in 428 attempts by 137 males and 491 attempts by 162 females (population-level analyses), and (C) changes in numbers of fledglings produced in 244 attempts by 107 individual males and in 266 attempts by 119 females that bred in one and the next year (individual-level analyses).

In all of the GAMM analyses of age effects on nest success (fledged ≥1 young) and fledgling production by goshawks there were large standard errors associated with the oldest age classes (12- to 15-year-old) due to small samples of old breeders. The binomial GAMM for nest success for known-age hawks showed no age effects on nest success in 164 breeding attempts by 58 males (*P* = 0.82) or by 88 females (*P* = 0.43) in 249 attempts (Fig 5A). Among ≥4-years-old hawks there was a significant decline in nest success with age in 428 breeding attempts by 137 males (*P* = 0.005) but no age effects in 491 attempts by 162 females (*P* = 0.09) (Fig 6A). There were no significant year effects on nest success for either male (*P* = 0.82) or female (*P* = 0.90) of known-age hawks or for male (*P* = 0.37) and female (*P* = 0.39) ≥4-years hawks.

#### Fledgling production

**Fig 7:**
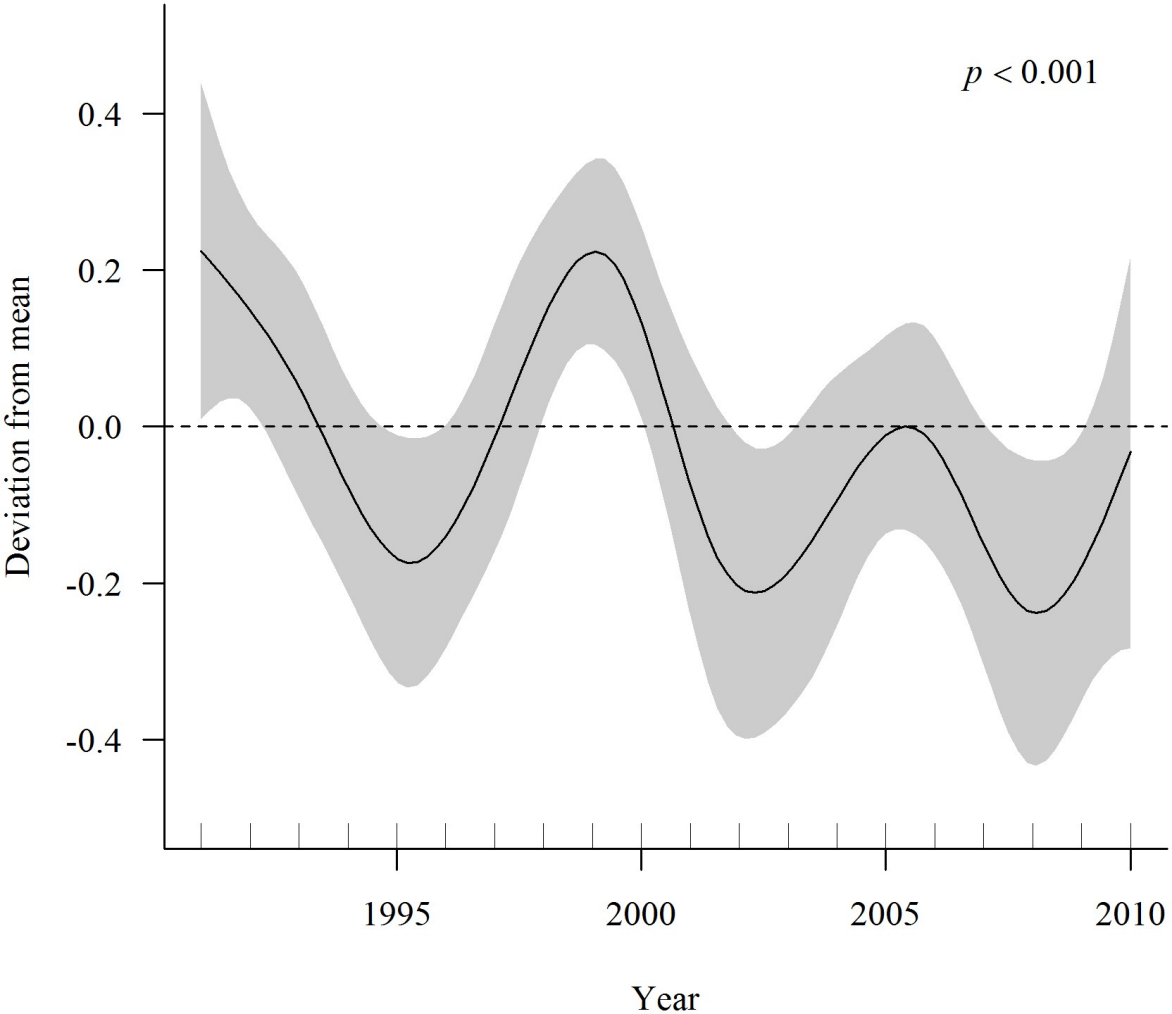
Year effect on fledgling production by goshawks. Expected year effect on fledgling production in 740 breeding attempts by 250 female (combined known-age and hawks aged ≥4-year-old on first breeding) northern goshawks from Poisson GAMM of population-level analysis of age-specific reproduction in Arizona, USA. The y-axis shows the deviation from the mean response and the shaded region depicts the 95% CI. The expected year effect on fledgling production by male goshawks (592 attempts by 195 males) was nearly identical to the female response.

The GAMM analysis of fledgling production by known-age hawks in the population-level analysis showed no significant age effects in 164 breeding attempts by 58 males (*P* = 0.64) or by 88 females (*P* = 0.70) in 491 attempts (Fig 5B). Likewise, among the ≥4-year-old hawks, there were no significant age effects in 428 breeding attempts by 137 males (*P* = 0.25) or 162 females (*P* = 0.46) in 491 attempts (Fig 6B). There were no significant year effects on males (*P* = 0.66) or females (*P* = 0.24) of known-age, but year effects were significant for both male (*P* = 0.03) and female (*P* = 0.06) ≥4-years-old hawks. A plot of year effects on fledgling production by 180 females of the combined known-age ≥4-year-olds clearly showed that 1991–1993, 1998–2000, and 2004–2006 were good breeding years, and 1994–1996, 2001–2003, and 2007–2008 were poor breeding years (Fig 7).

The individual level of the GAMM analyses of change in fledgling production in 85 sequential breeding attempts by 40 males and 133 attempts by 55 females of known-age that bred in one to the next year also showed no significant age effects (males, *P* = 0.27; females, *P* = 0.69) and no significant year effects on fledgling production (males, *P* = 0.50; females, *P* = 0.21) (Fig 5C). Likewise, for ≥4-year-old hawks there were no significant age effects (males, *P* = 0.94; females, *P* = 0.84) and no significant year effects on fledgling production (males, *P* = 0.52, females, *P* = 0.42) (Fig 6C).

#### Early breeding effects

Of the 28 breeding males and 41 females whose ages and lifespans were known (hawks in the 1992‒2000 breeding cohorts), 12 males (42%) and 14 females (34%) started breeding at age 2-years, and 12 males (42%) and 18 (44%) females started breeding at age 3-years (Table 4). Both male and female goshawks first breeding at age 2-years had shorter lifespans than individuals that delayed breeding until at least 4-years-old (Tukey’s multiple comparison tests; *P* < 0.1). Despite shorter lifespans, there were no differences in breeding lifespans or numbers of breeding attempts between any of the 3 age at first breeding groups. While there were no differences in nest failure rates among females in the 3 age groups, nest failure rates were higher (Tukey’s multiple comparison tests, *P* < 0.1) in 3-year-old first time breeding males than in males delaying until at least age 4-years or older. A likely consequence of the higher 3-year-old male failure rate was their significantly lower LR (*P* < 0.05) than of males delaying until 4-years or older (Table 4).

**Table 4:** Life history characteristics of goshawks first breeding at ages 2-, 3-, and 4+-year-olds. Tukey multiple comparisons of mean (± 95% CI) lifespans (years), breeding lifespans (years), number of breeding attempts, nest failure rates, and lifetime reproduction (LR) for 164 first breeding at ages 2-, 3-, and 4+-year-olds (4-year and older known-age + ≥4-years-old) goshawks in the 1992‒2000 cohorts in Arizona, USA.

Age 1st breeding	*n*	Lifespan^1^	Breeding lifespan^1^	Breeding attempts^1^	Nest failure rate^1^	LR^1^
Males						
2-years	12	5.83 (4.3–7.4) ^A^	3.83 (2.3–5.4) ^A^	3.17 (2.2–4.2) ^A^	0.29 (0.15–0.43) ^AB^	4.33 (3.2–5.5) ^**CD**^
3-years	12	6.58 (5.0–8.2) ^AB^	3.58 (2.0–5.1) ^A^	2.83 (1.9–3.8) ^A^	0.35 (0.19–0.51) ^A^	3.83 (2.7–4.9) ^**C**^
4+years	51	7.92 (7.2–8.7) ^B^	3.96 (3.2–4.7) ^A^	3.43 (2.9–3.9) ^A^	0.18 (0.12–0.23) ^B^	5.90 (5.2–6.6) ^**D**^
Females						
2-years	14	6.14 (4.7–7.6) ^A^	4.14 (2.7–5.6) ^A^	3.42 (2.5–4.4) ^A^	0.23 (0.11–0.35) ^A^	5.57 (4.3–6.8) ^A^
3-years	18	7.72 (6.4–9.0) ^AB^	4.72 (3.4–6.0) ^A^	3.56 (2.7–4.4) ^A^	0.20 (0.10–0.30) ^A^	6.33 (5.2–7.5) ^A^
4+years	57	≥8.16 (7.4–8.9) ^B^	3.93 (3.2–4.7) ^A^	3.30 (2.8–3.8) ^A^	0.17 (0.12–0.24) ^A^	5.63 (5.0–6.2) ^A^

^1^Non-overlapping letters A and B indicate significant differences between age groups at *P* < 0.1. Non-overlapping letters C and D indicate a significant differences between age groups at *P* < 0.05.

### Mate choice

**Fig 8:**
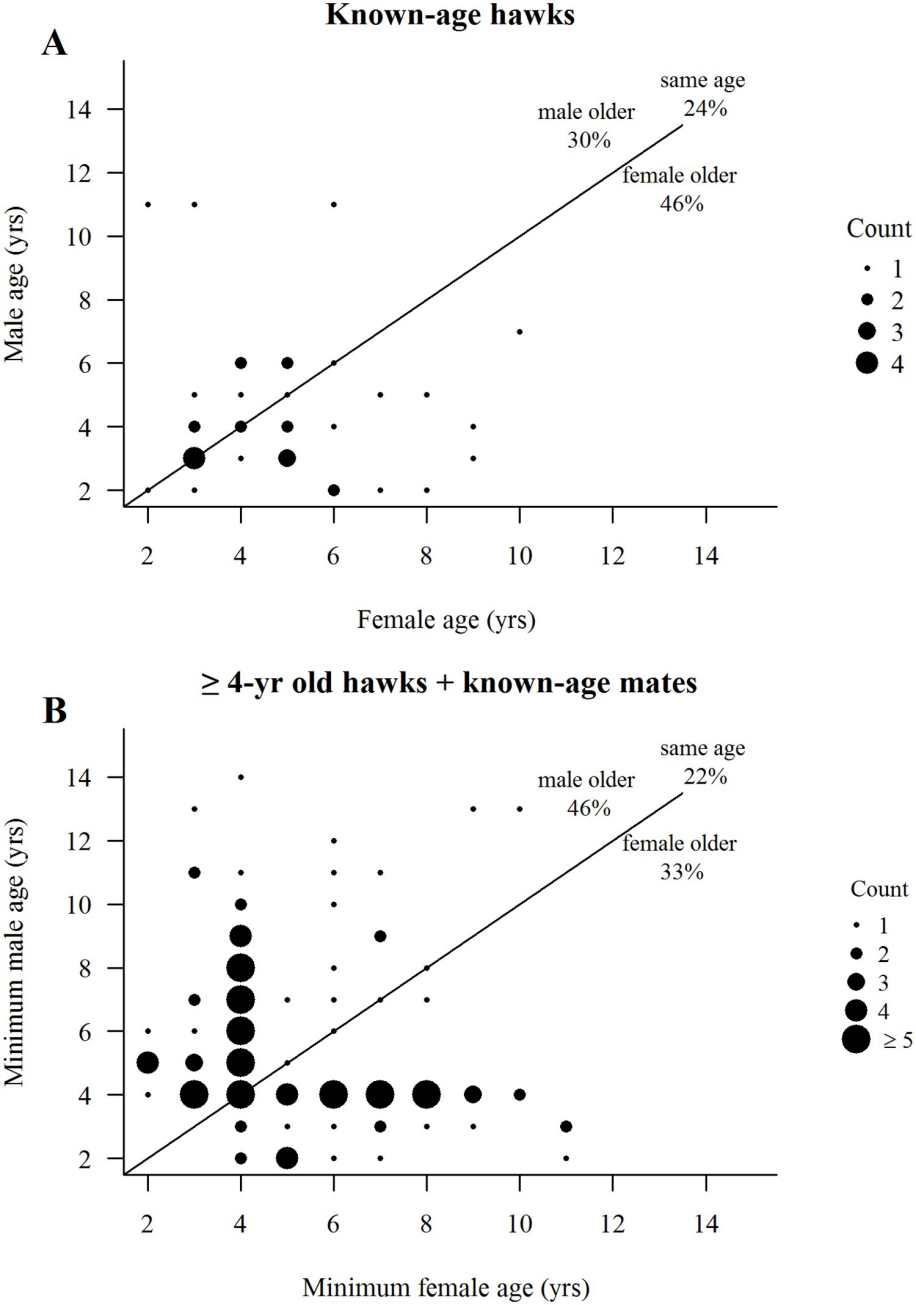
Is mate choice based on mate age? (A) Mate age composition of 37 pairings (33 males, 32 females) of known-age hawks, and (B) mate age composition of 169 pairings (120 males, 139 females) of hawks aged ≥4-year-old (including their known-age mates where relevant) in the year of pair formation by northern goshawks on the Kaibab Plateau, Arizona, USA. Size of dots indicates numbers of pairs observed in each mate-age category. Solid lines depict one-to-one relationships.

In the year of pair (or re-pair) formation in pairs where exact mate ages were known, only 24% of 37 pairs were of same-age hawks, males were older than their mates in 30% of pairings, and females were older in 46% of these initial pairings (Fig 8A). When known-age hawks were combined with ≥4-year-old hawks under the assumption that ≥4-year-old hawks were actually 4-years-old at first pairing, then 22% of 260 pairs at initial pairing were comprised of same-age hawks, males were older in 46% of pairs, and females were older in 33% of pairs (Fig 8B). Of course, the abundance of cases of pairs comprised of at least one 4-year-old (*n* = 169 pairs) in Fig 8B was due in large part to the assumption that all ≥4-year-olds were actually 4-years at initial pairing. Given our estimate (see above) that most (72%) ≥4 year-old hawks were actually 4-years (40%) or 5-years-old (32%) at first breeding, then our expectation is that a large proportion of individuals comprising the 4-year-old dots would shift to the right and/or up 1- to 2-years of age in the figure.

**Fig 9:**
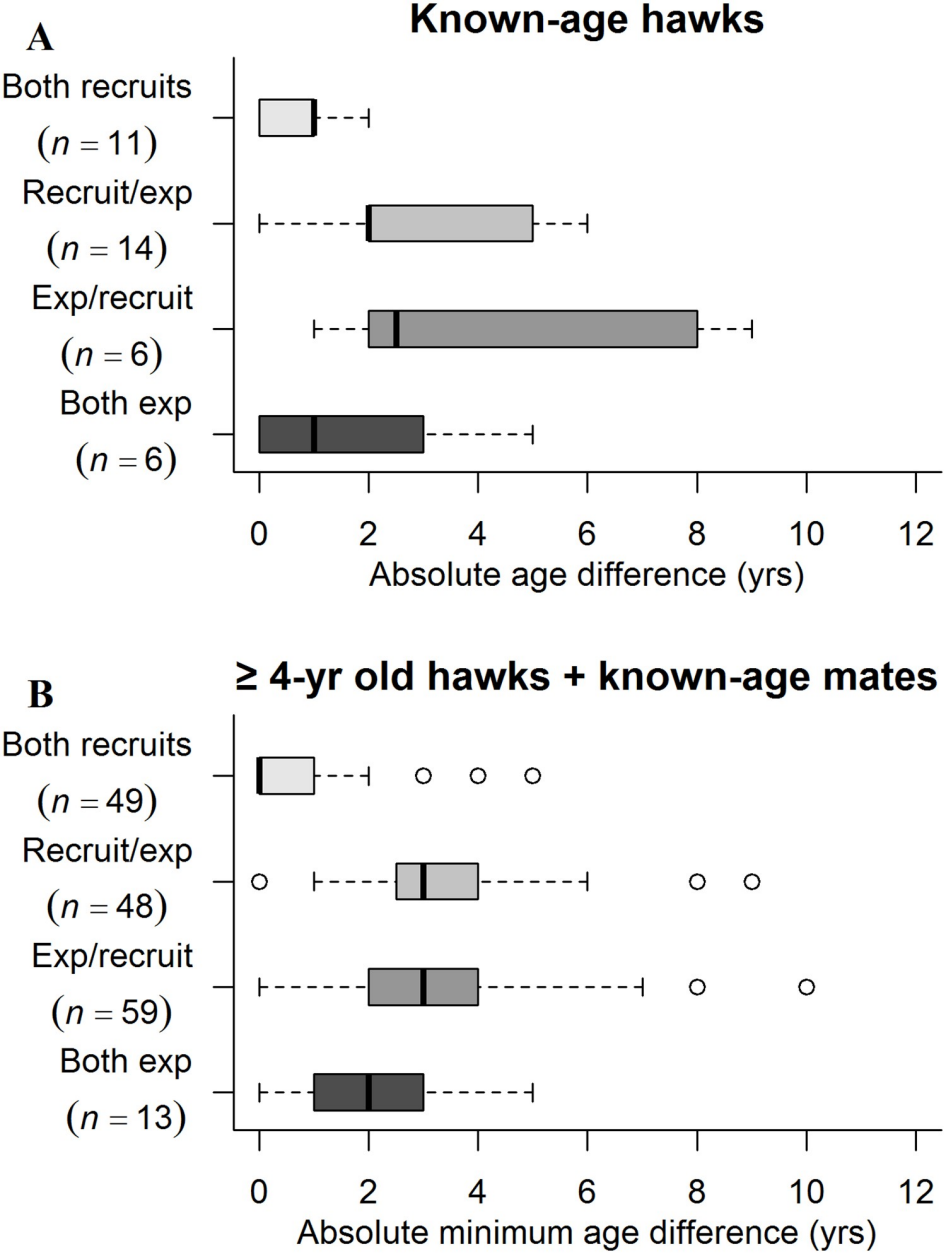
Is mate choice based on a mate’s breeding experience? Box plots of pair age differences in the year of pair formation with respect to a mate’s breeding experience by northern goshawks in Arizona, USA. Vertical bars are medians (i.e., 50^th^ percentile), boxes contain the central 50% of differences (i.e., bounded by the 25^th^ and 75^th^ percentiles), and dots are outliers. “Both recruits” pairs are both first-time breeders, “recruit/exp” pairs are male recruit/female experienced, “exp/recruit” pairs are male experienced/female recruit, and “both exp” pairs are both previous breeders. (A) Known-age hawks only and (B) hawks aged ≥4-years-old with their known-age mates where relevant.

There were no significant correlations between male and female mate ages in the initial year of pair formation for known-age hawks (*W* = 1.32, *P* = 0.19) or for pairs of hawks aged ≥4-years (*W* = -1.26, *P* = 0.21). Thus, mate choice was random with respect to mate age, a likely consequence of replacements of lost older mates by young individuals since the mean absolute age difference (female age–male age) of mates among known-age hawks was 2.24±0.38 years at first pairing. With respect to prior breeding experience at initial pairing by known-age hawks, recruit-to-recruit (no breeding experience) pairings comprised 30% (11 of 37) of pairs with a mean age difference of 0.6±0.2 years (Fig 9A). Male recruit-to-experienced female pairs comprised 38% (14 of 37) of pairings with a mean age difference of 2.9±0.6 years, while male experienced-to-female recruit pairs comprised 16% (6 of 37) of pairings with a mean age difference of 4.2±1.4 years. Finally, experienced-to-experienced pairs comprised 16% of pairings with a mean age difference of 1.6±0.8 years (experienced-to-experienced pairs were the consequences of a few hawks changing territories (breeding dispersals) and pairing with other experienced hawks). This pattern was only slightly different for ≥4-year-old hawks. Recruit-to-recruit pairings comprised 29% (49 of 169) of pairs, male recruit-to-experienced female pairs comprised 28% (48 of 169), male experienced-to-female recruit pairs comprised 35% (59 of 169), and experienced-to-experienced pairs comprised 8% (13 of 169) of pairings (age difference among these pairs were unknown because of uncertain ages of ≥4-year-old hawks) (Fig 9B). Among known-age hawks, variation in mate ages was least variable among recruit-to-recruit pairs, followed by experienced-to-experienced, and was most variable among pairs with a recruit and an experienced hawk. The lesser age variation in recruit-to-recruit pairs probably reflected the young ages of individuals in the pool of potential recruits, whereas the larger age variation in recruit-to-experienced pairs reflected the replacement of lost mates of older experienced hawks by younger recruits. For known-age hawks, pairwise comparisons of mean age differences between mates at first pairing with respect to pair composition and previous breeding experience showed an overall significant difference (*F* = 4.73, *P* = 0.008) only between comparisons of recruit/recruit pairs to male experienced/female recruit pairs (*P* = 0.01) and to male recruit/female experienced pairs (*P* = 0.04).

**Fig 10:**
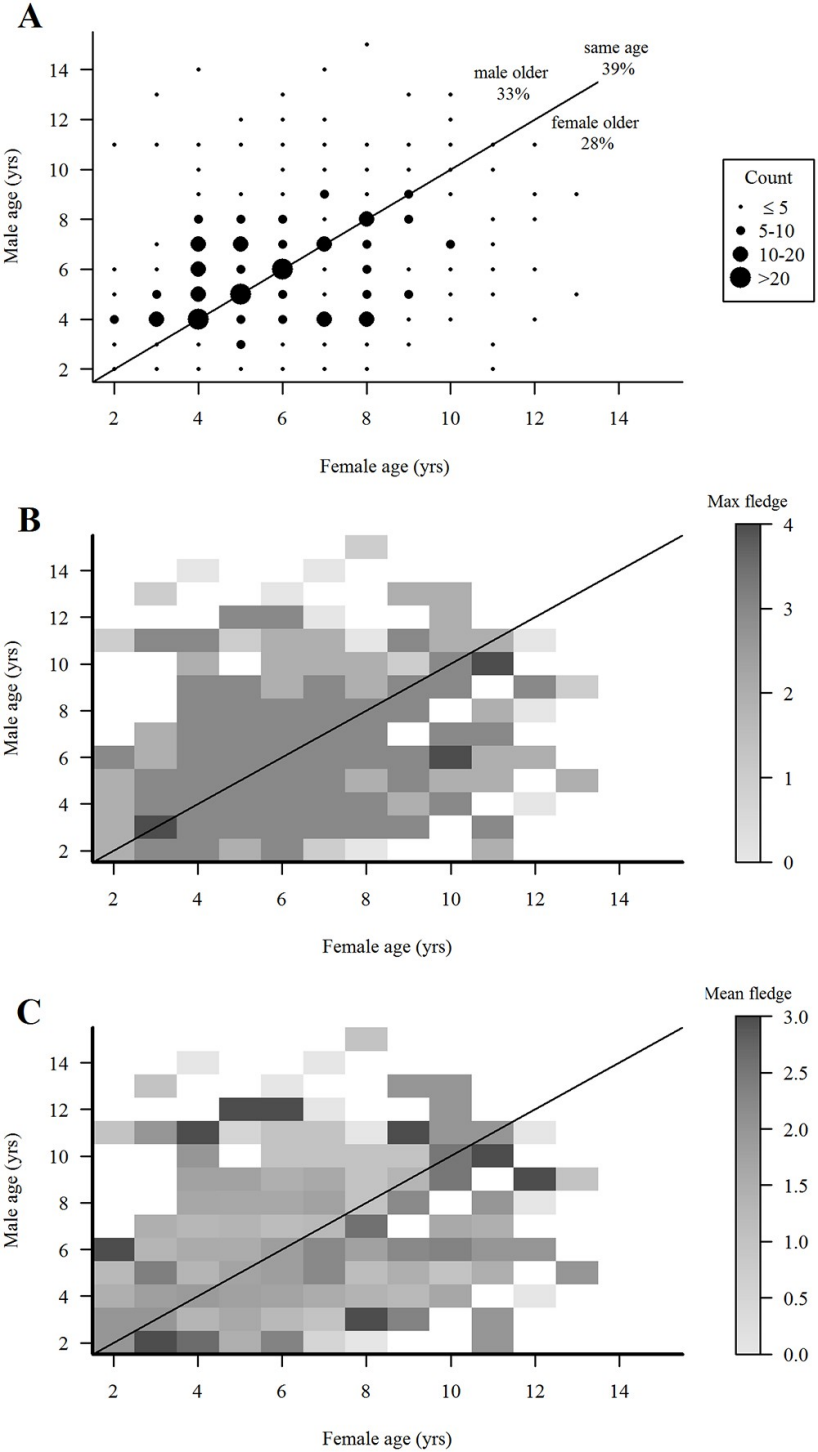
**(A) Variation in ages of pairs over time and fledgling production in all breeding attempts by pairs of different age compositions**. Variation in age composition and fledgling production through the duration of pair bonds (not limited to initial year of pair formation) in 505 breeding attempts by 168 male and 180 female northern goshawks (known-age + ≥4-years-old hawks). Size of dots indicates numbers of pairs observed in each mate-age category. (B) heat map of maximum number of fledglings produced in each breeding attempt by pairs of mixed ages, and (C) heat map of mean number of fledglings produced in these breeding attempts in Arizona, USA. Gray-scale indicates the maximum number of fledgling (includes 0 fledglings) produced by pairs in each mate-age composition category; white areas indicate no data.

A plot of the range of mate-age compositions at all breeding attempts of known-age combined with ≥4-year-old hawks (270 pairs, 574 breeding attempts) showed that 39% of breeding attempts were by pairs of the same-age, that the majority (66%) of pairings were comprised of mates whose ages differed by 4- to 8-years, and that 10 years was the maximum age difference between mates (Fig 10A). As with the initial pairings of ≥4-year-old hawks (Fig 8B), there was a tendency for males to be older than their mates through their breeding lifespans. Heatmaps of the maximum and range of numbers of fledglings produced in the 574 attempts showed a consistent maximum production of 3 fledglings by pairs of mixed ages between 3- and 9-years of age and a lower maximum of 0‒2 fledglings by pairs comprised of an older (≥12-years) male or female (Fig 10B and 10C). Whether lower production by older hawks reflects senescence is unclear because the small sample of old hawks limited the maximum and range of their fledgling production. Nonetheless, there was some evidence that male-older pairs were slightly more consistent in breeding performance than female-older pairs.

**Fig 11:**
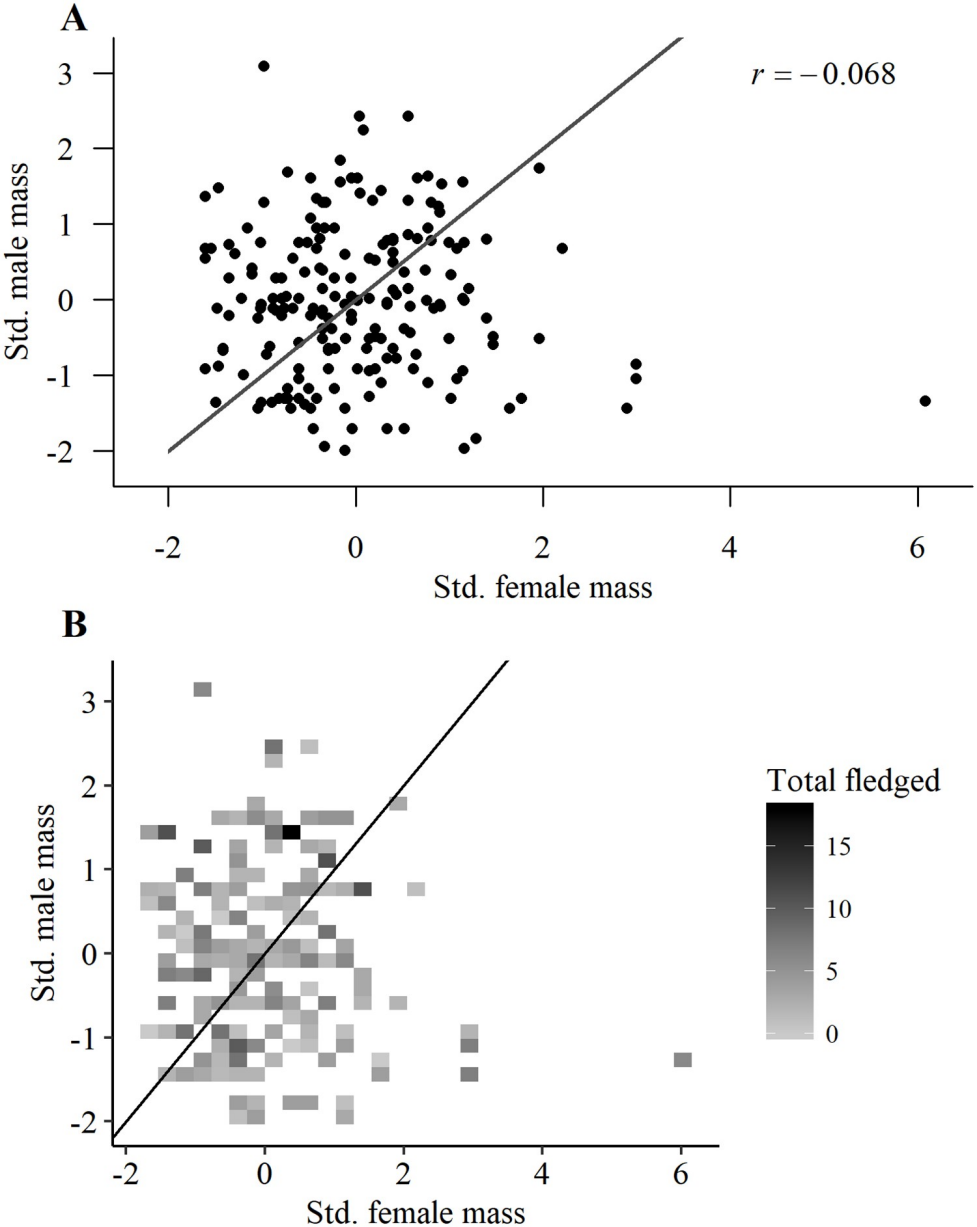
Is mate choice based on mate size or physiological condition? Scatter plot of *z*-scores for paired male and female goshawks showing (A) body mass and (B) heat map of the smoothed total number of fledglings per pair in 423 breeding attempts (not limited to initial year of pair formation) by 147 male and 151 female goshawks of known-mass on the Kaibab Plateau, Arizona, USA. Solid lines depict one-to-one relationships.

Our investigation of assortative mating based on body mass or size in the initial year of pairing (or re-pairing) included 147 male and 151 female goshawks whose mass, wing cord, and tarsom and tail length were known. The first PC for males explained 40.8% in the variation of morphological measurements and was significantly correlated with body mass (0.79%), wing cord (0.75%), and tail length (0.60%), and to a lesser degree tarsom length (0.31%). The first PC for females explained 32.0% of the variation in morphological measurements and was comprised primarily of a contrast between body mass (0.72%) plus tarsom (0.65%) and wing cord (-0.41%) plus tail length (-0.42%). Because body mass in our PCAs explained the majority of size variation in both males and females, we henceforth considered mass alone to be a sufficient index to both potential mate condition and body size. There was no correlation (*r = -0*.*07*, *P* = 0.34) between mate body masses of males and females at first pairing; like mate age, mate choice with respect to mate quality was random. Likewise, in a heatmap of LR_pair_ there was no pattern in total fledgling production among pairs of differing masses (Fig 11).

In our assessment of whether condition (mass) or size (the 1^st^ PC) of either males or females was related to LR_pair_, female condition in both GAMM models was insignificant (*P* = 0.74 for mass and *P* = 0.78 for the 1^st^ PC) but male mass and size were significantly related to *LR*_*pair*_ (*P* = 0.05 for mass and *P* = 0.006 for the 1^st^ PC), with larger males tending to produce more fledglings across the mating pair’s lifetime (S2 Fig).

#### Individual and environmental covariates

Our Poisson GLM analyses of individual and environmental covariates of LR included up to 75 male and 89 female goshawks (see S3–S7 Figs for box plots of raw data for each covariate and LR). We analyzed 2 different data sets for each sex (i.e., 4 data sets), one with 13 environmental and individual explanatory variables using a slightly smaller sample size (65 males, 86 females), including measured and imputed morphological data for each sex, and another set with 9 explanatory variables that excluded the morphological variables, *tarsom*, *wingC*, *mass*, and *tail* (*avgpermass* retained). For analyses including *tarsom*, *wingC*, *mass*, and *tailL*, the percent of 65 males whose 5 morphological variables were imputed varied between 1.5% (*tarsom*) and 6.2% (*wingC*, *tailL*), and for 86 fema1es with 6 imputed variables, 1.2% (*tarsom*) and 16.3% (*avgpermass*). For analyses excluding *tarsom*, *wingC*, *mass*, and *tailL*, the percent of 75 males whose morphological variables were imputed was 1.3% (*avgmaterank*) and 14.7% (*avgpermass*), and for 89 fema1es, 9.0% (a*vgmaterank*) and 19.1(*avgpermass*).

*Lifespan* was strongly correlated with other explanatory variables in all data sets (S1 and S2 Tables) while *avgpermass* and *mateswitch* were weakly correlated with other explanatory variables. Because of strong correlation with other variables, we excluded *lifespan* from our candidate model set. *Avgterrank* and *avgmaterank* were negatively correlated (due to the top producing territories and mates receiving ranks of 1 and less productive territories and mates ranks <1) with *lifespan* and *breedingattempts*. For both males and females *materank* was positively correlated with *nummates*. In both data sets, *nestfailures* were strongly positively correlated with *lifespan*, *breedingattempts*, and *nummates*; as lifespan increased, so did breeding attempts, number of mates, and nest failures. For both sexes, *Lifespan* was correlated positively with *agefirstbreeding*, reflecting an up to a 2-year cost of future lifetime by breeding before age ≥ 4-years (Table 4, S1 and S2 Tables). In males, *mass* was strongly correlated with *tarsom*, *wingC*, and *tailL*, but only with *tarsom* in females. This difference may reflect more variable female than male mass during the nestling period (when females were trapped and measured), the consequences of among-year and among-territory variability in prey abundance, differences in clutch and brood sizes, and male competencies in food provisioning.

There were 8 candidate models within 2 *AICc* units of the top model for females and 10 for males (S3 and S4 Tables). For both data sets, slope estimates (Table 5) were positive (*breedingattempts)*, negative (*nestfailures*), or negligible (*avgmaterank*, *agefirstbreeding*, *avgbrpairs*, *avgpermass*, *nummates*, *mateswitch*, and *avgterrrank*, *wingC*, *tarsom*, *mass*, and *tailL*), using α = 0.05. Results were similar with data sets that excluded *tarson*, *wingC*, *mass*, and *tailL*, with the exception of negative slope estimates with *agefirstbreeding* and *nestfailures* for females (Table 5 and S5 Table). The GLM analysis of LR showed that *nestfailures* and *breedingattempts* had relative importance of terms ≥0.8 in male and female data sets. For females, the slope of the relationship between LR and *agefirstbreeding* was negative (although not significant at *a* = 0.05), whereas in males, the relationship was not significant. Thus, there is some evidence that *agefirstbreeding* in females was linearly related to LR, where LR was slightly higher if they started breeding before age 4-years. Conversely, no significant linear relationship was found between *agefirstbreeding* and LR in males.

**Table 5:** Influence of individual and environmental effects on lifetime reproduction (LR) of goshawks. Slope parameter estimates from the generalized linear model candidate set with morphological data (*tarsom*, *wingC*, *mass*, and *tail*) included. Shown is adjusted standard error ($\hat{S}E_{adj}$), relative importance, and *p*-values for the model terms for the influence of individual and environmental effects on lifetime reproduction of 65 male and 86 female northern goshawks in Arizona, USA.

	Males			Females		
Model term	Estimate (±SE_adj_)	Relative importance	*P*^1^	Estimate (±SE_adj_)	Relative importance	*P*^1^
***Intercept***	1.465(0.137)		<0.001***	1.701(0.153)		<0.001***
***Avgbrpairs***	-0.020(0.053)	0.296	0.705	-0.011(0.038)	0.277	0.764
***Agefirstbreeding***	0.010(0.041)	0.254	0.798	-0.112(0.070)	0.849	0.109
***Avgmaterank***	-0.029(0.057)	0.362	0.615	-0.007(0.035)	0.265	0.834
***Avgpermass***	0.005(0.032)	0.234	0.874	0.002(0.028)	0.235	0.957
***Avgterrank***	-0.050(0.075)	0.456	0.506	-0.044(0.059)	0.510	0.453
***Breedingattempts***	0.646(0.081)	1.000	<0.001***	0.627(0.054)	1.000	<0.001***
***Nummates***	0.021(0.051)	0.312	0.682	0.017(0.042)	0.328	0.690
***Mate switch*, *no change in mate***	0.046(0.134)	0.551	0.729	-0.127(0.172)	0.433	0.463
***Mate switch*, *larger mate***^2^	0.201(0.223)	-	0.368	-0.097(0.157)	-	0.536
***Nestfailures***	-0.310(0.086)	0.997	<0.001***	-0.267(0.057)	1.000	<0.001***
***Mass***	0.001(0.030)	0.223	0.968	0.006(0.028)	0.252	0.835
***TailL***	0.008(0.032)	0.251	0.797	0.009(0.029)	0.269	0.769
***Tarsom***	0.009(0.037)	0.249	0.809	0.005(0.028)	0.247	0.846
***WingC***	0.012(0.040)	0.265	0.769	0.006(0.029)	0.252	0.822

^1^Significance level: *0.05, ** 0.01, *** 0.001.

^2^Mate switch is a categorical variable with multiple factor levels. Relative importance tracks the explanatory variable only, not each level; hence, the multiple rows in the table.

#### LR, recruitment, and fitness

**Fig 12:**
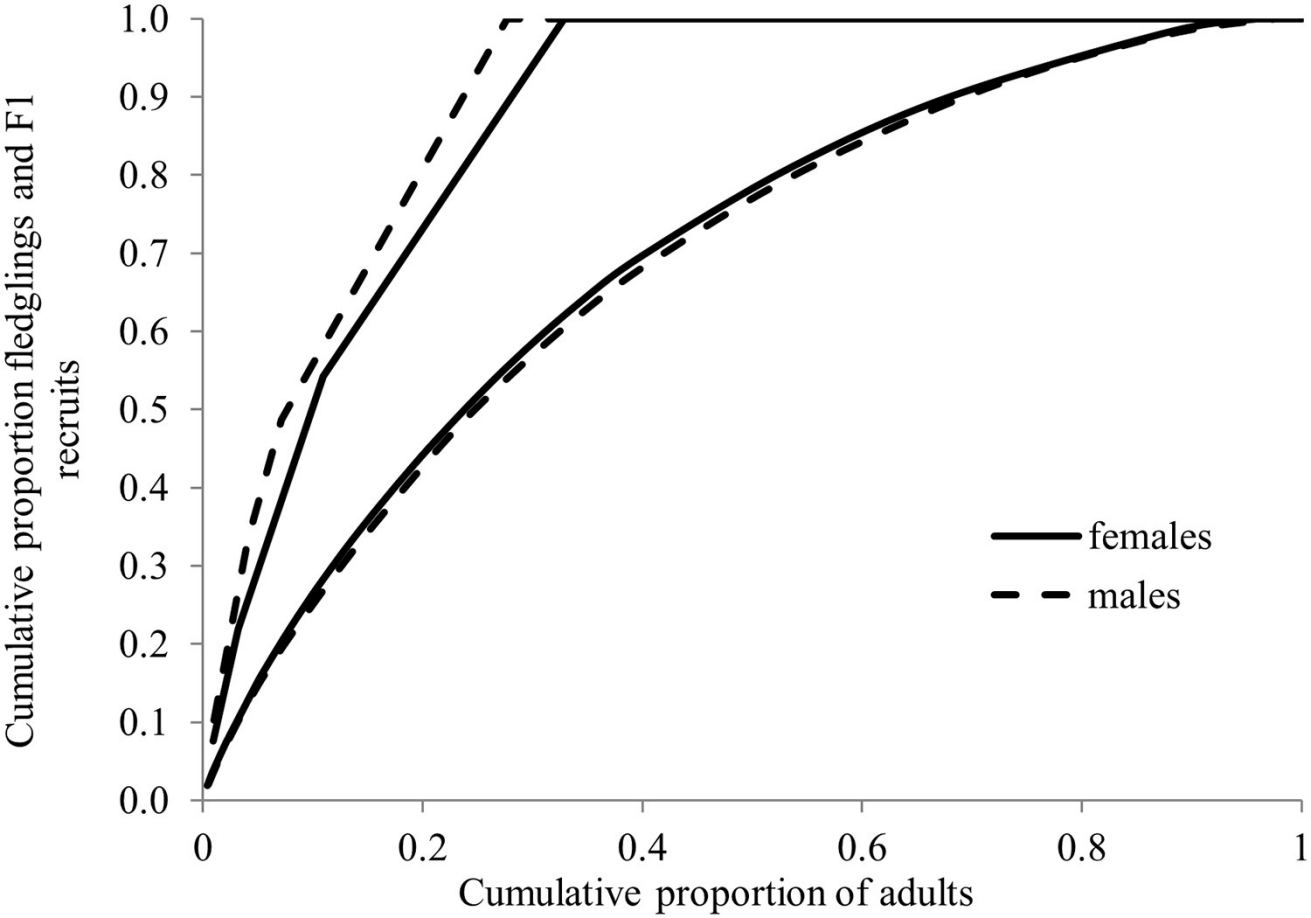
Proportion of total fledglings and F1 recruits produced by varying proportions of individual breeders. Proportional variation among 195 male and 250 female goshawks in total fledgling production and number of recruits to the local breeding population in Arizona, USA. Although 189 (96.4%) males and 238 (95%) females fledged young (lower curve), only 52 (27%) males and 71 (28%) females produced fledglings that recruited into the local breeding population (upper curve).

**Fig 13:**
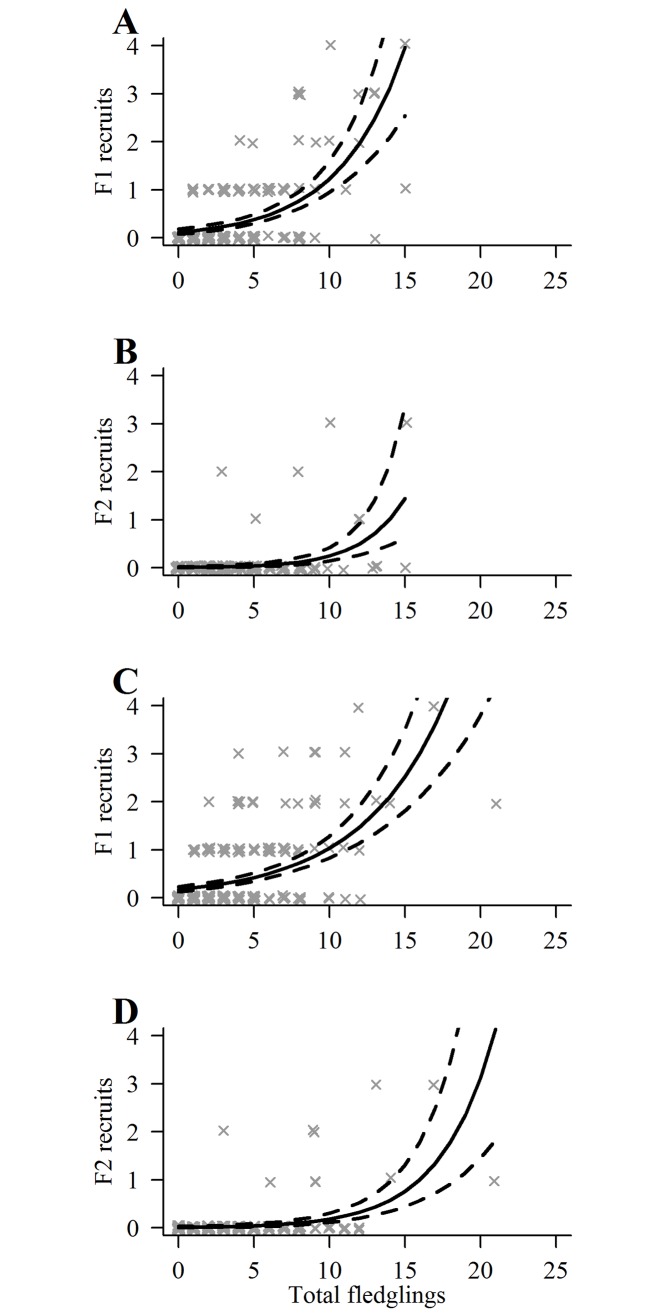
Number of children and grandchildren that locally recruited as breeders in relation to the number of fledglings produced by individual male and female goshawks. Number of banded descendants of 196 male (A, B) and 250 female (C, D) northern goshawks that recruited into the local breeding population as children (F1 generation) and grandchildren (F2 generation) in relation to the number of fledglings each breeder produced in Arizona, USA. Each ‘x’ represents an individual adult. Trend lines determined with Poisson regression with log-link functions.

Of 862 nestlings banded in 1991–2008 (nestlings banded in 2009–2010 excluded because of 2-year-old minimum age at first breeding precluded their recruitment in the final 2 years of the study), 104 (45 males, 59 females) were recruited into the local (*in situ*) breeding population, giving a recruitment rate of 0.12. These F1 recruits produced 490 nestlings that were banded, 17 (7 males, 10 females) of which recruited (rate ~ 0.04) as local breeders. The 17 F2 (grandchildren) breeders produced 120 fledglings that were banded, of which 6 (3 males, 3 females) recruited (rate ~ 0.05) as local breeders. Declines in recruitment rates over these generations mostly reflected the decreasing number of study-years available for successive generations to recruit. Numbers of local recruits from each year’s cohort of banded fledglings was positively correlated with the size of the cohort (S8 Fig). On combining known-aged hawks and ≥4-year-old breeding hawks, the cumulative distributions of fledgling production showed that about 26% of both genders produced about 52% of total Kaibab fledglings produced, and about 11% of breeders produced about 52% of local F1 recruits (Fig 12). While recruitments of F2, and especially F3, generations were likely to have been underestimated due to insufficient study years for recruitment to occur, only 4.1% of males (*n* = 7) and 4.6% of females (*n* = 10) produced all local F2 recruits and 1.7% of males (*n* = 3) and 1.4% of females (*n* = 3) produced all local F3 recruits. Thus, a relatively small proportion of the parental generation produced a disproportionate number of the total fledglings and local recruits. Of the 195 breeding males and 250 females, the average male produced 3.9 ± 0.34 fledglings, 0.4 ± 0.06 F1 recruits, 0.07 ± 0.03 F2 recruits, and 0.03 ± 0.02 F3 recruits and the average female produced 4.6 ± 0.39 fledglings, 0.4 ± 0.05 F1 recruits, 0.07 ± 0.02 F2 recruits, and 0.02 ± 0.01 F3 recruits. Our GLM analyses of LR of individual males and females and numbers of local recruits they produced showed that number of fledglings produced by both sexes was a highly significant predictor of the number of both F1 (males, *P* < 0.001, df = 193; females, *P* < 0.001, df = 248, Fig 13A and 13C) and F2 recruits (males, *P* < 0.001, df = 19; females *P* < 0.001, df = 248, Fig 13B and 13D). Thus, the number of fledglings produced by an individual was a good predictor of its fitness, as also reported for goshawks in Germany [19] (but see [16]).

#### Territory-specific reproduction: Breeding attempts, breeders, and mates

**Fig 14:**
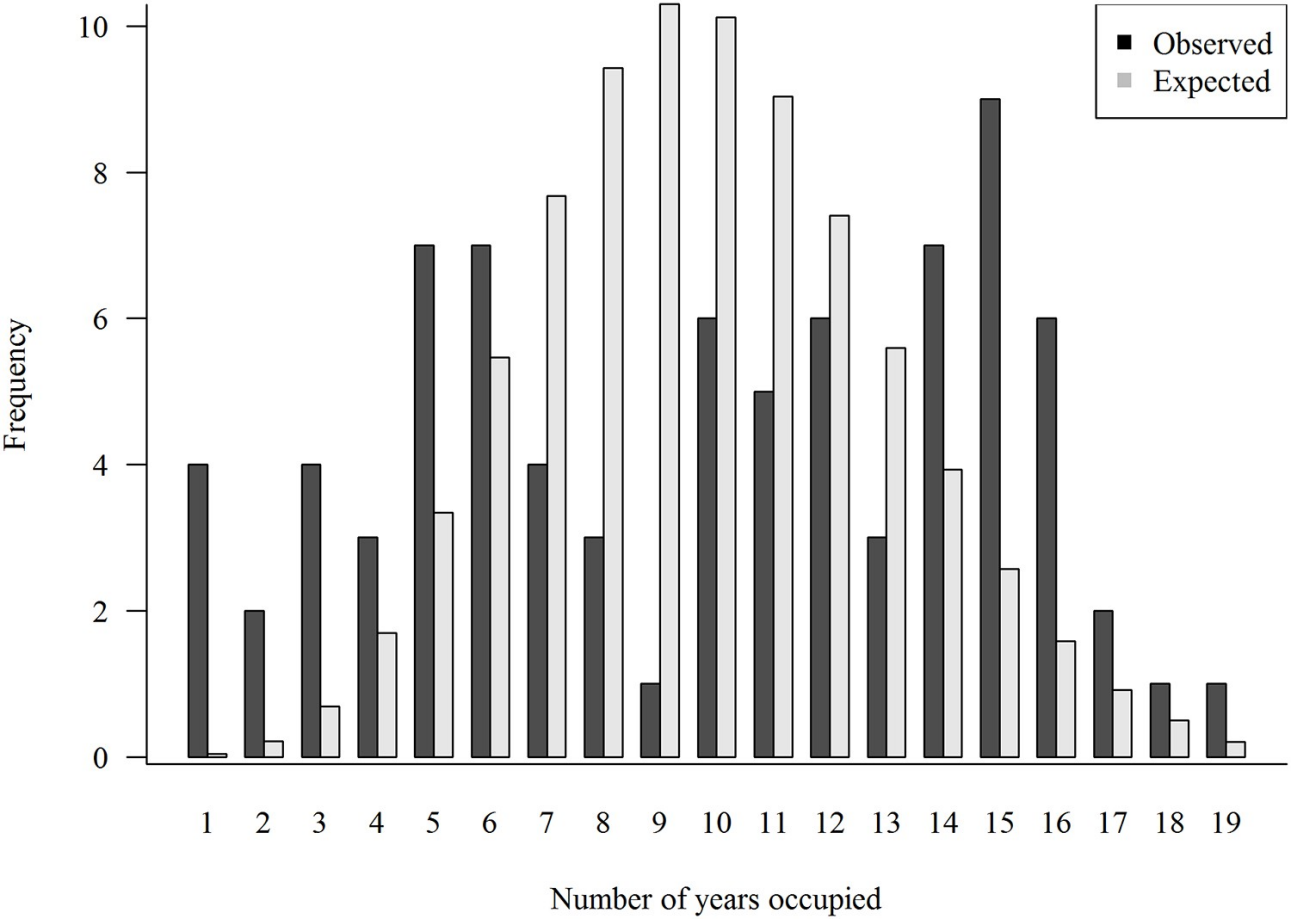
Breeding goshawks occupied territories non-randomly. Observed versus expected (from Poisson distribution) pattern of occupancy of 79 territories over 20 years by northern goshawks in Arizona, USA.

**Fig 15:**
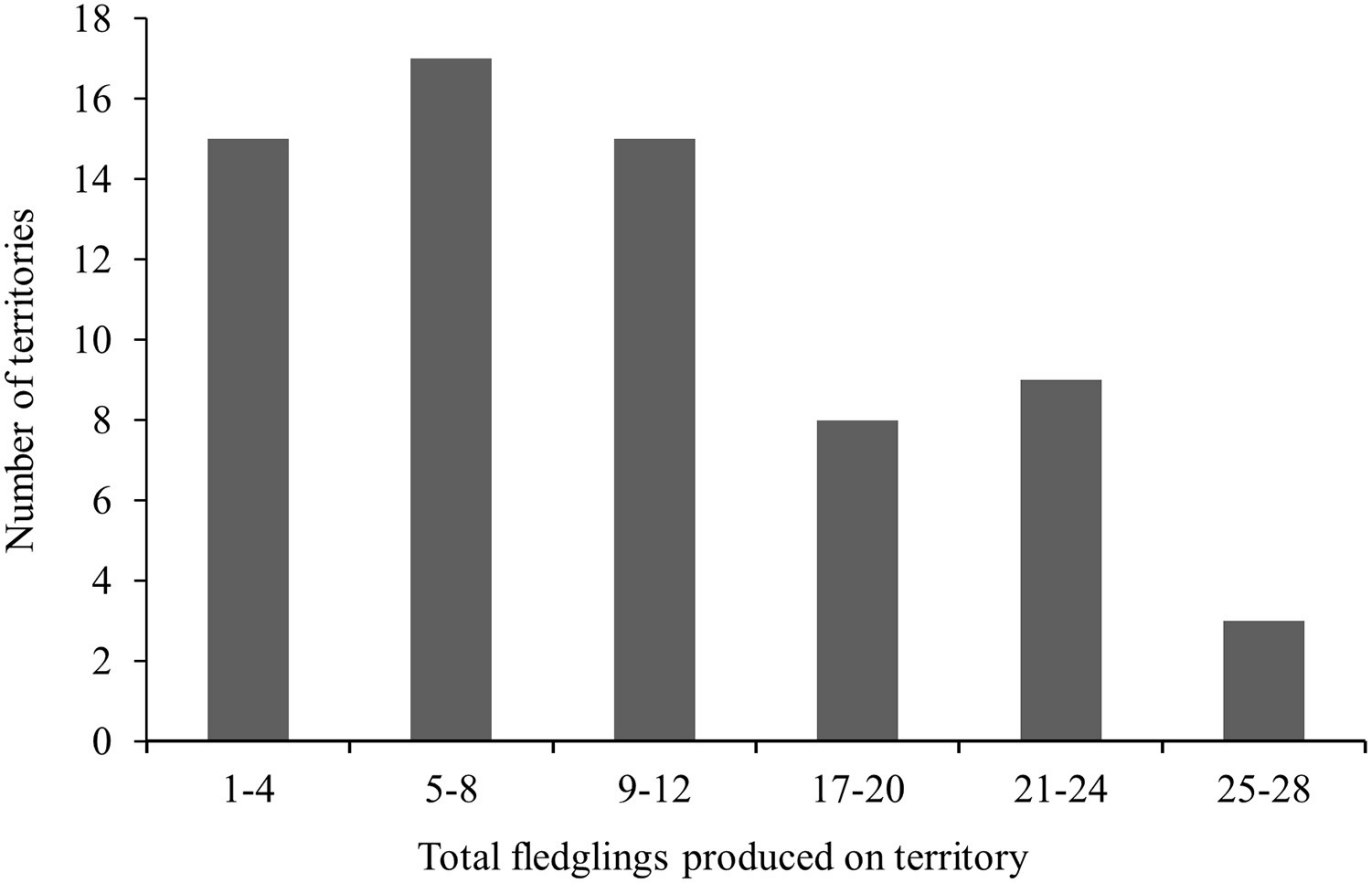
Total fledglings produced by goshawks on 79 territories monitored at least 18 years in Arizona, USA.

**Fig 16:**
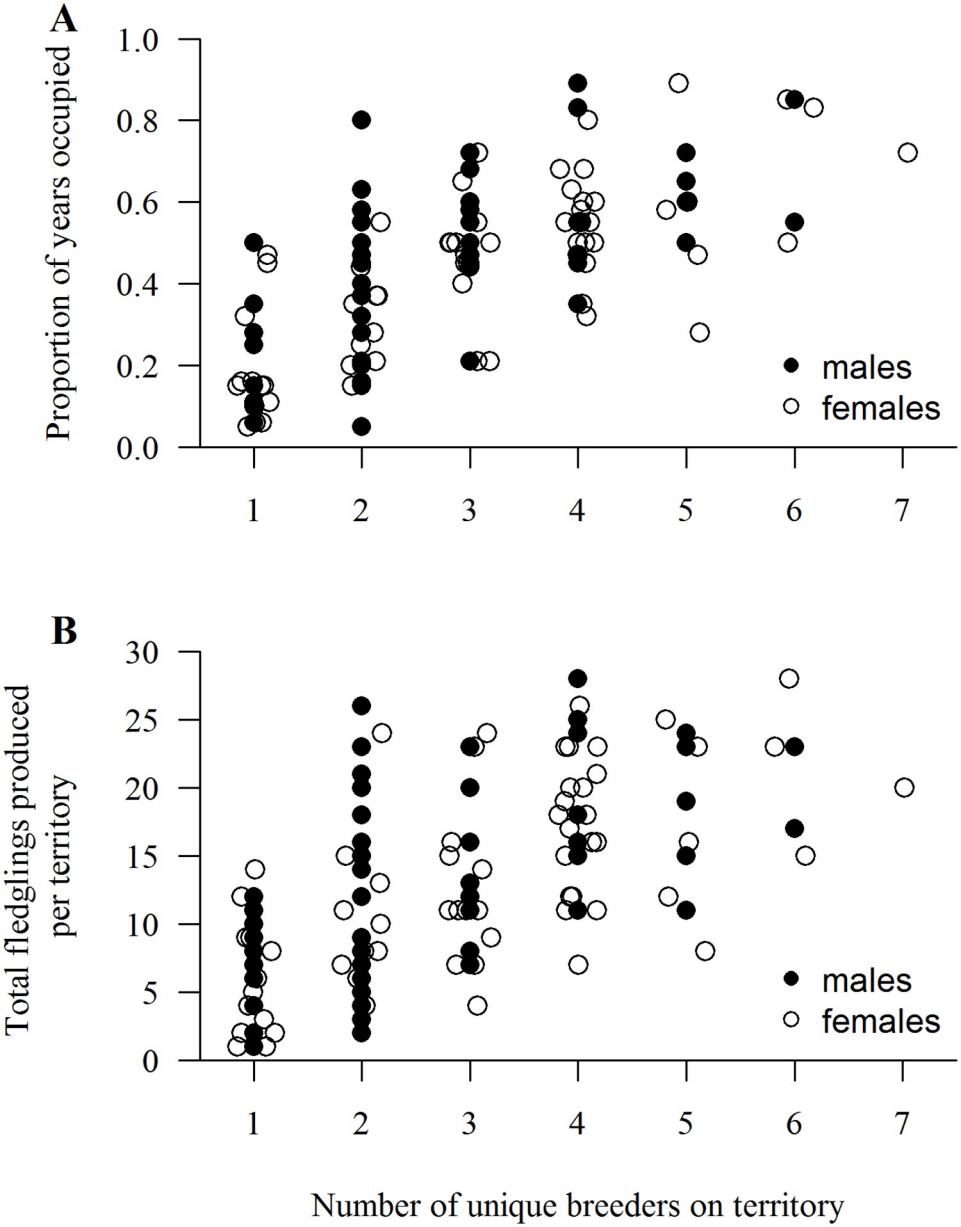
The number of unique breeders on a territory in relation to frequency of territory-specific occupancy and total fledgling production. (A) Numbers of sequential breeding male and female goshawks on each of 36 territories monitored for 20 years in relation to (A) the number of years each territory had breeders, and in relation to (B) the 20-year total fledglings produced on each of the 36 territories in Arizona, USA.

Our chi-square goodness-of-fit test for random vs. preferential nesting within 79 territories monitored for at least 18 years showed that goshawks occupied territories non-randomly across years ($X_{5}^{2}=126.35$, *P* < 0.001); some territories appeared to be occupied preferentially while others were avoided (Fig 14). The range in percent of years in which breeding occurred on the 79 territories was highly variable (5–89%, $\bar{x}=39\%\pm 0.02$, median = 4.5) as was the 18-year total number of fledglings produced on each (range = 1 − 28 fledglings; $\bar{x}=11.8\pm 0.81$, median = 11.0, mode = 11.0) (Fig 15). The number of unique sequential breeders on the 79 territories ranged from 1–6 males ($\bar{x}=2.7\pm 0.17$, median = 2) and 1–7 females ($\bar{x}=2.9\pm 0.18$, median = 3) (Fig 16). Some of the most frequently occupied and most productive (Table 6, Fig 16) of territories were occupied by as few as 2 long-lived goshawks while others had as many as 6 short-lived breeders. Number of lifetime mates per male on the 79 territories ranged from 1–4 ($\bar{x}=1.4\pm 0.05$ females, median = 1, *n* = 156 males) and 1–5 mates per female ($\bar{x}=1.4\pm 0.04$ males, median = 1, *n* = 248 females) and was as expected correlated with the breeding lifespans of individuals (males, *r* = 0.593; females, *r* = 0.704). Although long-lived individuals typically had more mates than short-lived hawks, several goshawks with breeding lifespans ≥8 years had as few as 1 long-lived lifetime mates (S6 Table).

**Table 6:** No differences in the proportion of 2- or 3-years-old breeders in the less productive than more productive territories.

**Less productive territories**								
**Territory cohort**	**Number of unique breeders**			**Number (%) of 2-year-olds**		**Number (%) of 3-year-olds**		
	male	female	total	male	female	male	female	total
20-yr, 36 territories	28	30	58	2(7)	3(10)	2(7)	1(1)	8(14)
19-yr, 26 territories	16	21	37	1(6)	3(14)	1(6)	1(5)	6(16)
18-yr, 18 territories	6	14	20	1(17)	0	0	3(21)	4(20)
**More productive territories**								
20-yr, 36 territories	63	70	133	6(10)	2(3)	2(3)	8(11)	18(14)
19-yr, 26 territories	30	43	73	1(3)	3(7)	3(10)	3(7)	10(14)
18-yr, 18 territories	16	29	45	1(6)	5(17)	2(13)	2(7)	10(22)

Comparisons of the percentages of total territory-specific breeders that were 2- or 3-years-old in less productive territories than in the more productive than the overall mean number of fledglings in three cohorts of territories (36 territories monitored 20 years, 25 territories monitored 19 years, 18 territories monitored 18 years) in Arizona, USA.

**Fig 17:**
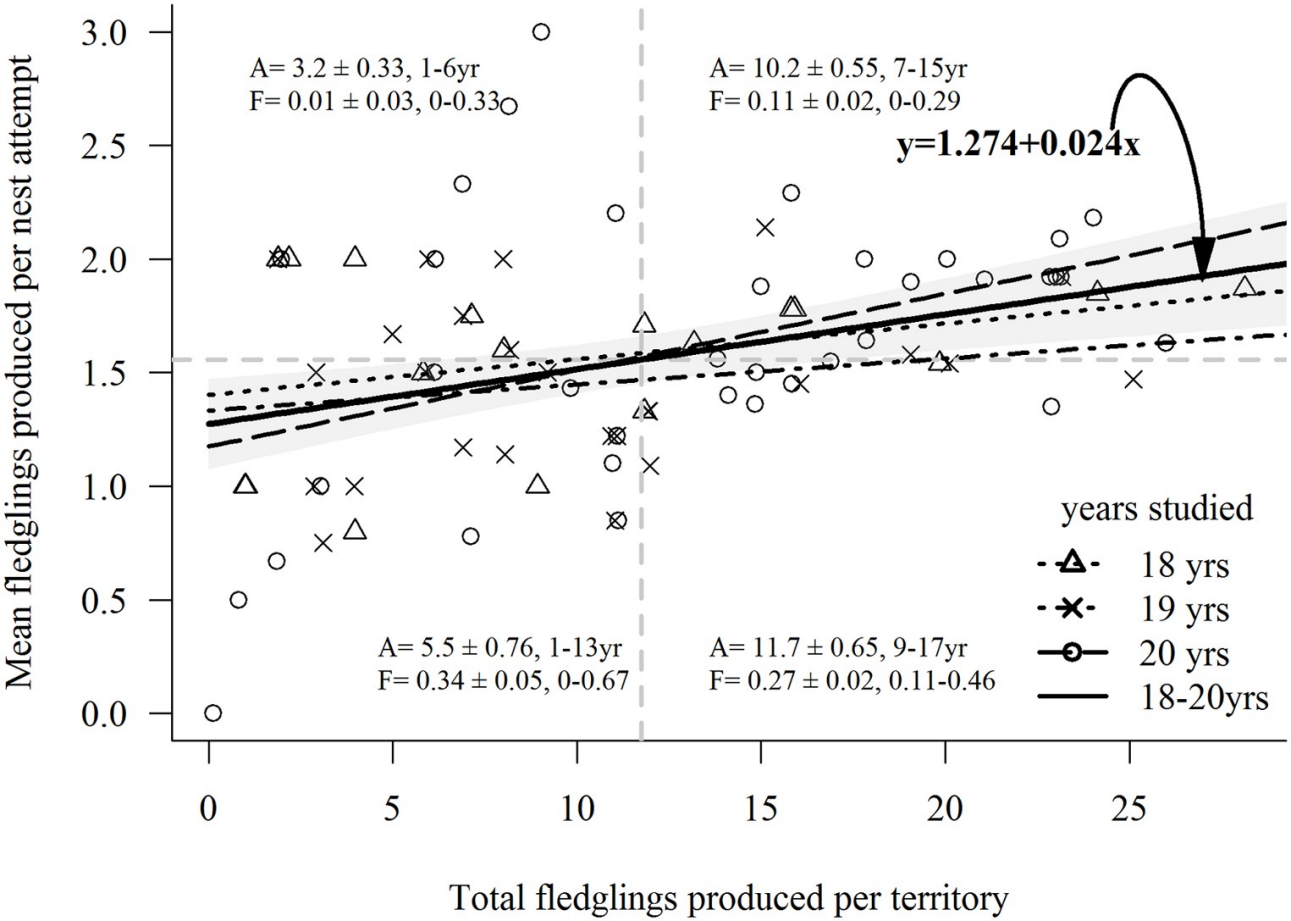
Are fewer fledglings per breeding attempt produced on less productive (low quality?) territories than on more productive (high quality?) territories? Linear regressions of mean numbers of fledglings produced per breeding attempt on low productivity vs. high productivity territories and the sum total fledglings produced by 3 different cohorts of territories (36 territories monitored 20 years [1991‒2010]; 25 territories monitored 19 years [1992–2010]; and 18 territories monitored 18 years [1993–2010]) by northern goshawks in Arizona, USA. Territories in each cohort are divided into 4 quadrants based on their mean total fledglings produced per study period (vertical line) and means of numbers of fledglings produced per breeding attempt (horizontal line). Means (±SE) and ranges of numbers of years that territories were active (A; eggs laid), and means (±SE) and ranges of nest failures (F) are provided for each quadrant.

**Fig 18:**
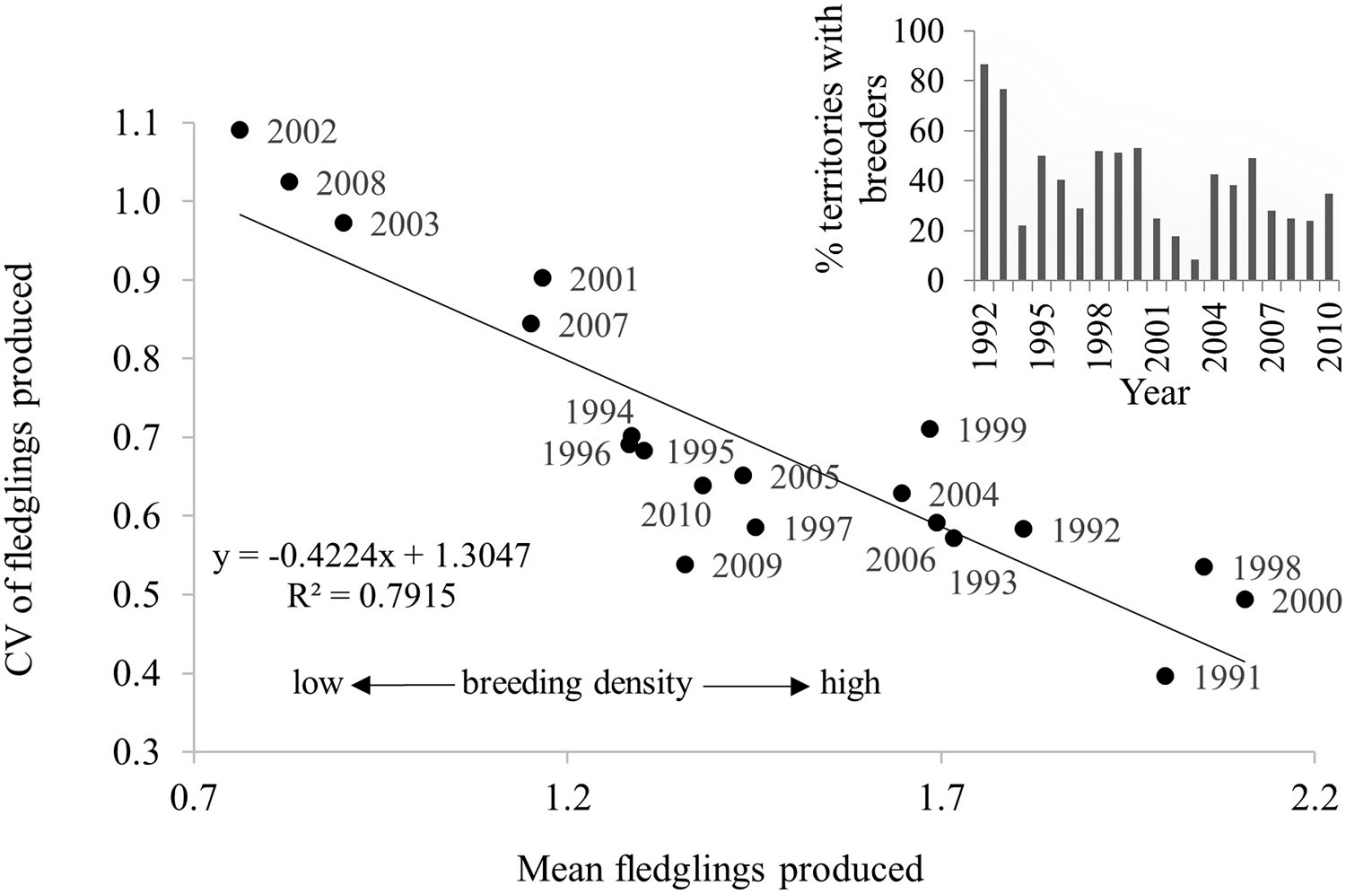
Variation in annual fledgling production and goshawk breeding density contradicts the ideal pre-emptive model. Contrary to predictions from the ideal pre-emptive model of habitat selection wherein the annual coefficient of variation (CV) in annual fecundity increases with breeder density as more lower quality territories become occupied and produce fewer and fewer young, the CV of annual fledgling production declined as breeding density increased (percent of territories with breeders; see inset) increased in Arizona, USA.

Long-term total reproduction on territories was a function of the number of successful breeding attempts and brood sizes per attempt. Mean fledglings produced per attempt, whether in frequently or infrequently occupied territories, was only weakly correlated (ignoring homoscedasticity violations) with total fledglings produced on 3 different cohorts of territories; 36 territories studied 20 years (1991–2010); 26 territories studied 19 years (1992–2010); and 18 territories studied 18 years (1993–2010). Because the 3 regression slopes were not different (*P* = 0.65), the 3 cohorts were pooled (Fig 17). Although mean fledglings produced per breeding attempt was consistently more variable in the less productive (those left of mean fledgling production) than in the more productive territories (those right of the mean), mean fledgling production per attempt in some less productive territories exceeded the highest mean per attempt in the more productive territories. Differences in total fledglings produced between high and low productive territories reflected differences in the frequencies of occupancy, brood sizes, and failure rates. Among the most productive territories, those in the upper-right quadrant were occupied by breeders a mean of 10.2 years, had a mean brood size of 1.9±0.04 and a mean nest failure rate of 0.11, whereas, while those in the lower right quadrant were occupied more frequently ($\bar{x}=11.7$ years), these territories had smaller broods ($\bar{x}=1.4\pm 0.05$) and higher nest failure rates ($\hat{p}=0.27$). Among the less productive territories, those in the upper-left quadrant had the largest broods ($\bar{x}=2.0\pm 0.09$) and the lowest failure rates ($\hat{p}=0.01$) overall territories, but were occupied a mean of only 3.2 years, most (67%, 42 of 63 breeding attempts) of which occurred during best of the 3-year periods of good breeding (1991–1993, 1998–2000) when brood sizes were large and nest failures low (see [75]). Because territories in the lower-left quadrant were occupied about 2 years longer ($\bar{x}=5.5$ years) than upper-left territories, breeding in the lower-left territories more often occurred during poor breeding years leading to smaller brood sizes ($\bar{x}=1.0\pm 0.06$) and more frequent nest failures ($\hat{p}=0.34$). Contrary to IPD predictions, the annual CV of fledgling production among all territories decreased as breeder density increased (Fig 18) and there were no differences in the frequency of sub-adult (2- and 3-year-old) goshawks breeding in the more- vs. less frequently occupied territories (Table 6).

## Discussion

### Influence of lifespan and age at first breeding on LR

LR of Kaibab Plateau goshawks increased with number of breeding attempts, which was strongly correlated with lifespan, breeding lifespan, and nest failures. Because most goshawks started breeding at age ≥4-years, lived less than 8 years, had breeding lifespans of about 4 years, and typically skipped breeding in 1‒3 years [75], the typical breeder made 3 lifetime breeding attempts and produced a total of 5.3 (males) and 5.8 (females) fledglings. Nonetheless, the salient feature of this population was a strongly right-skewed among-individual variation in LR. A minority (25%) of males and females produced 2 or fewer fledglings, the consequence of 18.7% of males and 21.3% of females breeding only once and about 3% of both sexes producing clutches but failing to fledge any young in their lifetimes. Only 15% of males and 16% of females produced 10 or more fledglings, with a maximum production of 19 fledglings by males and 23 by females. The extent of individual variation in LR among Kaibab goshawks was similar to LR variation among female goshawks in Bavaria [106], in male Cooper’s hawks (*A*. *cooperii*) in New York [107], female sparrowhawks (*A*. *nisus*) in Scotland [108], in several other hawks and owls [109–111], and in birds in general [3, 112].

LR can be affected by age of first breeding, which is often influenced by population structure and dynamics [3, 27, 113]. Density-dependent breeding by young raptors is driven by the availability of breeding territories, food, or mates and typically manifests as changes in the number of young breeders in a population as breeder density fluctuates [3, 29, 114]. When a breeding population falls below habitat saturation the proportion of young breeders typically increase (resource permitting), but as density increases and fewer territories are available, young hawks may wait years for vacancies [26, 27]. Both goshawk sexes are capable of breeding at 9 months of age and the proportion of these first-year breeders in populations have been reported as high as 35–40%, with first-year females more commonly breeding than first-year males [30, 115, 116]. In highly size-dimorphic raptors such as goshawks, males typically defend territories and provision their mates and broods with food while females remain at nests incubating, brooding, and defense of nests. Less frequent breeding by young males may reflect increased costs of reproduction due to their limited experience at foraging and territorial defense [28, 34, 83, 117]. More frequent nest failure and lower LR of 2- and 3-year-old Kaibab males compared to the less frequent failures and higher LR of 2- and 3-year-old females and those males delaying breeding until >3-years-old attests to the importance of male experience. We find it interesting that, against expectations, 2-year-old first breeding Kaibab males had higher LR than 3-year-old first time breeders. We suspect that this difference was related to the fact that 5 (42%) of the 12 2-year-old male breeders first nested in 1992 and 1993, by far the best of breeding years [77], whereas none of the 12 3-year-old first breeding males nested in these years. Unusually high prey abundance in 1992‒1993 likely countered the effect of inexperience on the LR of 2-year-olds.

Interestingly, while the mean lifespan of 2- and 3-years-old Kaibab males and females breeding on the Kaibab were shorter by as much as 2 years than lifespans of both sexes that delayed till age >3-years, there were no differences in either breeding lifespans and or number of breeding attempts between these 2 groups. The similarities may be related to the duration of the trough-to-peak periodicity of good and poor breeding conditions on the Kaibab Plateau [75]. Many first-time Kaibab breeders (young or older) laid eggs in the initial years of a 3‒4-year period of improved breeding conditions whereas only a fraction of territories had breeders during a subsequent 3‒4 year period of poor conditions (see Fig 4). The periodicity of breeding year quality likely played a role in restricting the breeding lifespans of many of the hawks in each age group to 3 and 4 years, and may have also resulted in underestimates of the lifespans of some hawks. Three to 4 years of good breeding (when most pairs laid eggs) combined with low resighting probability of non-breeders during the following poor year period (when few pairs laid eggs) would underestimate lifespans of any individuals that survived into but not through the poor years to breed (and be resighted) again the next good breeding period. Like females on the Kaibab Plateau, female goshawks in Germany showed no differences in breeding lifespans among females that bred early ($\bar{x}=2.8-\text{year}$) versus those that bred later in life. However, unlike the slight differences in LR of 2- and 3-year-old Kaibab females, the German females first breeding at 2-years had significantly lower LR than those starting at 3-years [19].

The absence of breeding by goshawks <2-year-old on the Kaibab Plateau was intriguing. According to the delayed breeding hypothesis (i.e., individuals delaying first breeding are of higher quality) birds that delay breeding are expected to have higher LR than those that breed at an early age [118, 119]. However, the not uncommon breeding by ≤1-year-old goshawks elsewhere in their geographic range and occasional breeding by 2- and 3-year-old goshawks on the Kaibab Plateau suggests there may be a fitness benefit to starting early in life [19, 33]. The temporal variability in breeding by 2- and 3-year-old goshawks on the Kaibab appears to reflect the limited life experience of these young individuals in an environment with highly variable food resources where even experienced adult breeders frequently do not produce eggs in periods of low food abundance [77, 86]. It is not surprising then that even less experienced goshawks in their first or second year would breed on the Kaibab. Nonetheless, given the average 3 lifetime-breeding attempts irrespective of age at first breeding, it would seem adaptive to start early in life, especially for females as there were no LR costs for starting at least at 2-years. While breeding early may bet-hedge against the probability of death before reproduction [105, 120, 121], breeding by 2- and 3-year-old Kaibab goshawks was clearly opportunistic and dependent on food abundance (sufficient to produce a clutch) as well as availability of mates (i.e., vacancies on territories). Unfortunately, the extent to which vacancies existed on the Kaibab was difficult to determine because of the low detectability of non-breeders. The likelihood of accrued adult mortality through non-breeding periods and the absence of occupancy evidence (hawk sightings, refurbished nests, molted feathers, feces) in many territories during low breeding years suggested that vacancies were not uncommon [75]. In fact, we suspect that acquisitions of territories by some 2- and 3-year-old hawks occurred during low breeding periods but went unobserved due to the inability of the hawks to produce a clutch and low detection probabilities of non-breeders. Increases in 2-year-old breeders as breeding conditions improved affirmed the presence of young floaters as well as vacancies. The steep declines to nil in numbers of 2-year-old breeders on deterioration of breeding conditions was likely a manifestation of the inability of young inexperienced breeders to procure sufficient food in years when many experienced adults failed to lay. The high proportion of 2-year-old breeders in 1991 (6%) and 1992 (13%) (Fig 4) indicated that breeding conditions were extraordinary in 1991 through 1993, resulting in the highest percentages of territories with breeders in our 20-year study (86% in 1992, 77% in 1993) and was coincident with a record-long El Niño wet period [122] that increased primary forest productivity, bird and mammal prey abundance, and goshawk breeding [75].

There were a few Kaibab goshawks of both sexes that were not observed breeding until age 9-years. These hawks may have acquired territories when younger but may have been unable to produce clutches for multiple years due to a string of poor breeding years. Alternatively, these hawks may have dispersed to a monitored territory from a yet to be discovered territory where they previously bred (more likely to occur in the early years of our study when fewer territories were being monitored), or they may have bred elsewhere and immigrated to the Kaibab. Due to high territory fidelity of breeding goshawks on the Kaibab Plateau and elsewhere [34, 78, 80, 87] we contend that older first-breeding Kaibab goshawks were mostly individuals that acquired territories earlier in life but were delayed in breeding by food limitation.

### Age-specific reproduction

Reproductive performance in many long-lived birds initially improves with age with a strong increase early in life followed by a plateau. While detrimental effects of aging on reproduction might be expected, such effects are difficult to document due to the sharp declines in numbers of old individuals within populations. The only significant effect of aging in our 12 GAMM analyses of age-specific reproduction was on nest success (a nest attempt producing ≥1 fledgling) of ≥4-year-old males (Fig 6) where there was a steady decline in nest success with increasing age, although this effect was likely strongly influenced by two males breeding at age 14-years whose nests failed (for annual nest success on the Kaibab see [77]). There was, however, an incipient (nonsignificant) concave curve in the individual-level analyses of age-specific fledgling production of known-age males (Fig 5). Both curves are suggestive of a gradual decline with age in a male’s ability to provision food to his female and brood. The otherwise flatness of the curves in both the population-level and individual-level analysis of male and female age-specific fledgling production on the Kaibab contrasted with the concave curves of increased fledgling production early in life followed by either a stable productivity through age 10-years or a decline with age in productivity by European goshawks and several other raptors [18, 33, 123, 124]. Because the concave curves in European goshawks started with significantly lower fledgling production by first-year (9-months old) and 1-year-old hawks [19, 33], we suspect that the absence of concave curves on the Kaibab may have been due to the absence of breeding by hawks younger than 2-years. Our finding of no significant age effects on fledgling production from age 2- to 15-years is similar to Abt’s [124] finding that brood size in 919 goshawk breeding attempts in Germany (see [36]) was remarkably stable from age 4-years to over 10-years.

Based on the association between extensive inter-annual variations in food abundance and variation in nest success and brood size of Kaibab goshawks [77], we were surprised to find a significant year effect only on age-specific brood size in the population-level analysis of ≥4-year-old goshawks. This shows that brood size was more sensitive to environment fluctuations than nest success and that the non-detection of a year effect on brood size in other age-specific analyses may have been related to sample size. The number of broods in the population-level analyses of ≥4-year-old hawks (*n* = 919) was nearly double the nest attempts included in the individual-level (*n* = 510) and as much as 2- to 4-times as many in the analyses of known-age hawks (population-level, 413 attempts; individual-level, 218 attempts).

### Mate choice

Researchers are often interested in measures that are proxies for fitness in order to predict individual differences in survival and reproductive success. Such proxies are typically breeding age, breeding experience, body condition, and body size, and these are likely to be mediated by individual quality because those of higher quality are presumed better at acquiring resources and increasing survival and reproduction. While assortative mating in birds is common in long-lived, territorial, and monogamous species, the patterns (expressed at the population level) and preferences (expressed at the individual level) of mate choice are poorly understood [125, 126]. Age-based assortative mating where birds mate with individuals of certain ages has been shown to be adaptive because adult-adult pairings are typically more productive than inexperienced-inexperienced and mixed-age parings [37, 41, 127]. In raptor studies where only two age classes, juvenile and adult (ensemble of all precisely-aged adults), were included, age-based assortative mating was found; there were fewer juvenile-adult pairing and more juvenile-juvenile and adult-adult pairing than expected by chance. However, when precisely-aged adult-adult pairs were considered there was no tendency for hawks of similar age to be paired [37–39]. While age assortative mating can result from active mate choice, other factors such as little variation in age at first breeding, high survival, and high site and mate fidelity, can produce strong correlations between mate ages [40, 41]. While Kaibab goshawks exhibited wide variation in age at first breeding (2- to 9-years-old), most (86‒88% of known-age hawks) started breeding between ages 2- and 5-years, had relatively high adult annual survival rate (0.78 peak survival at age 5-year [75]), and showed strong mate and territory fidelity [77, 78], our analyses provided no evidence of active age-based mate choice. The small proportion (22‒24%) of same-age pairs (Fig 8) probably reflected the relative abundance of 3- and 4-year-old goshawks in the floater pool, especially in the years of expanding numbers of breeders, whereas the more frequent (56‒79%) pairing of variable-aged pairs was the result of new recruits (typically 2- to 5-years-old) pairing with older breeders whose mates had disappeared.

Body size in birds may indicate a potential mate’s quality because an individual’s condition predicts its fitness inasmuch as those in better condition have more resources to allocate towards increasing their reproductive fitness [11, 12, 128, 129]. In size-dimorphic raptors a potential mate’s size may indicate its quality because pairs with larger females (or smaller males) have higher fitness as a result of a directional selection arising from their different efficiencies in their divergent breeding sex roles [130–132]. As in the lack of age-based assortative mating, we found no evidence of body condition or size-based assortative mating in Kaibab goshawks; mate choice on the Kaibab appeared to be and on a “first-come, first-serve” basis, an expectation if available mates were in short supply.

### LR: Individual and environmental correlates

The salient reproductive feature of goshawks in our study was extensive among-individual variation in lifetime production of fledglings and recruits. Cumulative distributions of individual male and female fledgling production showed that only a third of breeders produced two-thirds of the population’s fledglings, and increasingly smaller proportions of these breeders produced local (*in situ*) recruits in 3 succeeding generations. Production of the majority of fledglings and subsequent recruits by a small number of breeders has been documented in other raptors [109, 111, 133, 134] and in birds in general [15, 135, 136]. The significant relationship between individual fledgling production and numbers of recruits by goshawks on the Kaibab Plateau supports the contention that individual LR on the Kaibab is a good predictor of fitness.

Our investigations into how individual and environmental conditions combined to shape a goshawk’s lifetime breeding performance and fitness potential revealed that numbers of breeding attempts and nest failures accounted for most of the variation in male LR, and number of breeding attempts, number of nest failures, and age at first breeding accounted for most of the variation in female LR. The slopes of the relationships in the GLMs between LR and age of first breeding for females were negative, although not significant in the model including all morphological variables, indicating that females had marginally higher LR if they started breeding at a younger age. When age at first breeding was treated categorically and pooled for all females breeding for the first time at or after age 4 years (Table 4), this relationship was washed-out and no significant differences were found. Similarly, in our categorical analyses of the effects of age at first breeding on LR, males breeding for the first time at 2 years-old had significantly lower LR than those delaying until age 4 years or older, whereas no significant relationship was found when age at first breeding was treated continuously in the GLM analyses (Table 5). These discrepancies were due to the few outlying individuals that delayed breeding until well past age 4 years (i.e., one male first breeding at age 9 but none breeding between 6 and 8 years-old) as well as the timing of breeding by many males for the first time at 4 years during periods of good breeding years. Thus, despite the prospect of a reduction in lifespan of up to 2 years in both males and females that first bred before age 4-years, neither showed substantial differences in LR compared to hawks delaying till age 4-years. On the other hand, males breeding before age 4-years suffered increased nest failures, where nests of males that delayed breeding until age 4-years or more were half as likely to fail. Why then did male goshawks on the Kaibab attempt to breed before age 4? Life-history theory predicts that fitness is highly sensitive to changes in age at first reproduction [16], because early breeding increases the potential number of lifetime breeding attempts, starting as early as possible is intuitively a good strategy. Nonetheless, breeding early in life can depress survival probability and accelerate reproductive senescence [137–139]. Decisions of when to begin breeding can also be affected by the environmental conditions experienced by individuals either early or later in life. Given that both goshawk genders are mature sexually before the end of their first year and life expectancy for Kaibab goshawks began to decline after age 3-years (S7 Table), it was surprising not to see more 2- and 3-year-old breeders. Breeding by young goshawks on the Kaibab was less common than in other goshawk populations, and breeding by 2- and 3-year-old Kaibab goshawks was almost entirely limited to the first and second year of a series of years of improving breeding conditions. Due to accrued mortalities of territory owners during a series of poor breeding years, vacancies on territories increased opportunities for 2- or 3-year-old goshawks to recruit and begin breeding as conditions improved. Despite potentially shorter lifespans, increased nest failures, and reduced LR among males, the majority (72%) of 2- or 3-year-old males successfully fledged young. These young recruits augmented the much more frequent recruitment of full adult (≥4-years-old) goshawks as breeding conditions improved and the proportion of territories with breeders increased [77].

The negative correlations between *avgbrpairs*, the annual average density of breeding pairs (Table 1, S1 and S2 Tables), and a hawk’s breeding lifespan was the opposite of our expectations. The negative correlation likely reflected interactions between the timing of first breeding and the length of a hawk’s breeding lifespan. Long-lived individuals (e.g., breeding lifespans >10 years) experienced multiple 3–4 year periods of good (high breeder density) and poor breeding years (low density), whereas breeding by short-lived hawks more commonly started and finished in a sequence of 3–4 good years when breeding density was high. The mix of periods of high versus low quality breeding years experienced by long-lived hawks lowered the average number of breeding pairs during their reproductive lifespans relative to short-lived individuals. The positive correlation between age of first breeding and lifespan reflected the shorter lifespan of goshawks first breeding at ages 2- and 3-years and longer lifespans of hawks that delayed breeding till later in life. Long-lived hawks also had more opportunities to breed, had more mates, and more nest failures than short-lived hawks. It remains to point out that our inability to resight non-breeders (common during poor breeding years) may have underestimated lifespans because non-breeders could not be resighted as alive.

Among environmental effects, territory quality and mate quality (both estimated by total fledgling production) were positively correlated with a goshawk’s breeding lifespan, number of breeding attempts, and number of mates. However, due to strong territory fidelity by Kaibab goshawks [78], territory quality could not be separately estimated from hawk quality because the two were confounded; the more long-lived and productive an individual goshawk, the more productive its territory. The same confounding occurred between individual quality and mate quality because of strong mate fidelity. None of the individual morphological characteristics (mass, tail length, tarsom length, wing cord) of goshawks explained variation in LR for either sex. This contrasted with goshawks on the Baltic island of Gotland where males with the greatest body mass produced more fledglings, and where male wing length was shorter in rabbit-poor areas and longer in rabbit-rich areas where more fledglings were produced [140].

Age and breeding experience of mates (especially males) may affect the reproductive performance of goshawks on the Kaibab Plateau. Contrary to our expectations that females should avoid pairing with inexperienced (younger) males given their more frequent nest failure, the mean age of male mates was younger than their females across the majority of age-classes (Fig 8). Younger males may be a manifestation of a male floater pool younger on average than female floaters because of lower male than female survival as both juveniles and adults [77, 141, 142]. Nonetheless, mate ages were most similar in the 3- to 6-year age-classes, perhaps a consequence of the floater pool being comprised of young goshawks, the similarity of breeding lifespans of both sexes, and high mate fidelity. Increases in age variation among pairs after age 8-years reflected mortalities of original mates and the predominance of young goshawks in the floater pool of potential replacements.

### Reproduction on territories: Individual versus habitat quality?

Our ultimate research objective is to identify the compositional and structural components of forest vegetation that confers habitat quality to goshawks. Our aim is to determine the relationship between the species composition and 3-dimentional structure of forest vegetation in territories and the long-term demographic performance of territory occupants. It is often assumed that individuals either initially settle in high quality territories or disperse from poor quality sites to better habitat to improve their reproductive success [63, 133, 143]. However, the specific reproductive performance of individuals may also reflect their innate abilities to contest for territories or mates, use resources efficiently, and survive longer to reproduce more often (individual abilities are known to improve with age and experience [19, 33, 140]). Thus, territory-specific estimates of habitat quality based on reproductive output alone may be substantially biased by variation in individual quality [63].

The question of habitat vs. individual quality (i.e., fitness) has important conservation implications, especially where species are conserved via habitat management [24, 96, 144]. However, if individual quality is a major contributor to demographic performance, management could be counter-productive unless the two qualities are positively correlated [24]. Understanding the extent of synergy between individual and habitat quality requires reliable metrics of individual fitness, territory-specific habitat composition and structure, and the behavioral processes and choices made in territory selection ‒ choices that distribute goshawks among habitats [63]. Habitat selection is a hierarchical process of responses (choices) that typically result in a disproportionate use of habitat patches that differ in resources and environmental conditions [58, 62]. Because habitat preferences of individuals are assumed adaptive, there is an expectation of a congruence between a species’ evolved habitat preference and an individual’s fitness [59, 145, 146]. This expectation is supported by numerous bird studies reporting differential reproductive performance in different habitats [3, 56, 144]. However, because little is known how individual quality vs. habitat quality affects individual performances, most habitat quality studies treat conspecifics as ecological equivalents [147].

Prospecting animals select habitats based on a variety of cues: the composition and/or structure of vegetation [49, 148, 149]; food [50, 150, 151]; presence of nest sites, competitors, and predators [52, 53, 152]; interspecific attraction or other social information such as old nest structures [153–155]; and a potential mate’s characteristics such as body condition or size that might reflect the quality of a mate’s habitat [11, 42, 58, 156]. The usefulness of environment and social cues of course depends on their temporal variability and predictability at appropriate spatial scales. Researchers have variously estimated habitat quality by frequency of occupancy, survival of occupants, annual and total reproductive rates, dates of occupation in seasonal settings, frequency of receipt of breeding dispersals, and quantity and quality of food and nest sites [24, 157–159]. Because some territories are occupied more frequently than others and occupancy rates are known to be correlated with other measures of territory quality, occupancy rate is considered a consistent measure of territory quality [143]. However, occupancy assumes that prospecting individuals, whether first-time or dispersing breeders, choose territories based on habitat assessments and that choices are not spatially and temporally constrained by the number of territories an individual is capable of assessing [160]. Finally, occupancy measures of habitat quality may be ambiguous in long-lived birds with strong site fidelity because individuals remain faithful to their territory even if better territories become available [143].

On the other hand, individual quality has been estimated variously by age, morphology (e.g., tarsus, wing, tail length, body condition, body mass), longevity (survival), reproduction, and LR (a composite measure of survival and reproduction) [123, 161, 162]. Accordingly, extensive heterogeneity in individual survival and reproduction has been documented in many species [105, 107, 108], and several studies report strong correlations between individual LR and number of recruits. Thus, LR in an expanding list of species have proven to be reliable measures of fitness [13, 14, 105].

Selecting the best habitat requires prospectors to visit a sample of habitats, determine the best-of-*n*, and return to settle (assuming a vacancy) in the best [163]. However, the number of territories a young, inexperienced goshawk could adequately assess for food availability or vegetation composition and structure is likely constrained perceptually, spatially, and temporally. First, young prospecting Kaibab goshawks would have to assess conditions in large areas (i.e., home ranges can be up to 3,500 ha; [32]). Second, prospecting time is constrained by a need, driven by limited lifespans and breeding opportunities, to quickly acquire a territory and begin breeding. Observations of juvenile non-breeding goshawks on the Kaibab suggested that prospecting initially involves continuous wandering until appropriate selection cues are encountered when, thereafter, movements are limited to smaller areas [164]. Based on ages at first breeding, initial prospecting may persist for several years and may even be employed by post-breeding adults as evidenced by reports in goshawks and other raptors [165–167] of extra-territory excursions and intraspecific nest intrusions and occasional short breeding dispersals by Kaibab goshawks. Nonetheless, near-lifetime fidelity to territories was a prominent behavior of Kaibab goshawks suggesting that, because most spent their entire breeding lives in their initial territory, prospectors ought to devote substantial time and energy assessing habitats.

Krüger and Lindström (66), Squires and Kennedy (72), and Kenward (34) suggests that selection of breeding habitat, and the subsequent distribution of goshawks among habitats, follows the ideal pre-emptive distribution (IPD) model [70, 168, 169]. However, if goshawks follow the IPD model, and if there is synergy between individual and habitat quality, then individual quality may mediate, offset, or exaggerate habitat quality effects [24]. If reproductive costs and energy allocation tradeoffs in a habitat are affected by individual quality, then individual quality must be controlled for when making inferences about habitat quality based on the individual demographic performance. Expected outcomes under IPD settling are: territories are occupied non-randomly over years, some are frequently occupied and others rarely; more of the infrequently-occupied territories become occupied during periods of population growth; among-year fledgling production is more variable in less-frequently occupied territories; there is a positive relationship between mean fledgling production on a territory and its coefficient of variation (CV) as a population’s breeding density increases; and less-frequently occupied territories are occupied more commonly by young breeders [73, 151, 170].

In support of the supposition that Kaibab goshawks settled according to the IPD, Kaibab territories were (1) occupied non-randomly year-to-year, (2) more of the less-frequently occupied territories transitioned to being occupied as the density of breeders increased and declined as density declined, and (3) fledgling production per breeding attempt was more variable in less-frequently occupied territories. However, contrary to expectations, the annual CV of fledgling production across all territories decreased as breeder density increased, and there were no differences in the frequency of sub-adult goshawks breeding on more- or less-frequently occupied territories (Fig 18). Based on the IPD and the convention that habitat-specific measures of individual survival and reproduction are gold standard measures of habitat quality [58], the more consistently occupied and productive territories were expected to contain high quality habitat, high quality hawks, or both. Yet, this expectation was controverted by our data showing that (1) over the long-run, the top producing (>20 fledglings) territories were occupied by as few as 2 long-lived (high fitness) goshawks to as many as 7 short-lived (low fitness) breeders, that (2) despite greater variation in fledgling production per attempt in the less productive territories (<12 fledglings), many of the less productive territories were as equally productive per attempt as the most productive of territories, that (3) instead of habitat cues *per se*, prospecting hawks appeared to have been attracted by settled conspecifics and 70% of recruits filled vacancies on territories, and that (4) the quality of territorial singletons appeared to have little effect on a prospector’s habitat choice because choice on the Kaibab was random with respect to mate age, condition, or body size. Mate choice seemed to occur on “first-come, first-serve” basis, an expectation if potential mates were in short supply. A “first-come, first-serve” mate choice also raises the possibility that, over the long-run, differences in the fitness of successive breeders ‒ the individual contributions to the long-term production of fledglings in a territory ‒ might be negated by a “washing out” of among-individual differences (*sensu* [24]). A washing-out seems corroborated by the fact that top producing territories were either occupied by a few long-lived (high fitness) goshawks, multiple short-lived (low fitness) individuals, or long-lived breeders paired with multiple short-lived mates.

Identifying the cues used by Kaibab goshawks during habitat selection is complicated by the fact that competition among prospectors for territories was likely low due to apparent low territory occupancy in 14 of the 20 study years (1994‒1997, 2003‒2010) [75]. Low occupancy suggested that the resource most limiting to prospectors may not have been habitat at all but was instead the availability of territorial singletons. Furthermore, the top fledgling-producing territories on the Kaibab occurred across the entire elevational gradient from low elevation ponderosa pine-dominated territories to upper elevation wet mixed-conifer-dominated territories, and, based on our qualitative assessment of the vertical and horizontal structure of the conifer forests across the gradient, other than tree density there was little heterogeneity in habitat structure *at the territory scale* (~11.3 km^2^). The lack of strong among-territory structural differences combined with any perceptual, temporal, and spatial constraints on young prospecting goshawks raises questions regarding the direct role of habitat cues during habitat selection by prospectors.

That habitat selection may not be directly cued to habitat seems problematic given the substantial evidence that habitat affects fitness through variations in environmental conditions and resources that in turn affect individual survival and reproduction [58, 146, 171]. With the exception of several long (<15 km), narrow (<0.5 km) meadows, several high-severity fire scars (see Fig 2 in [75]), and scattered small (2–17 ha) shelterwood and seed-tree harvested areas (each of which retained some mature trees), forest cover on the Kaibab Plateau was unbroken. Despite gradual increases in tree density and species diversity with increasing elevation (see Study Area), forest structure on the Kaibab was relatively homogeneous and potential nest sites (small areas with large trees and relatively high tree and canopy density [172, 173]) were abundant and randomly distributed across the study area [174]. In fact, the near continuous conifer forest cover on our study area was considerably less heterogeneous than the degree of habitat heterogeneity in other raptor studies where territories were unequally and non-randomly occupied ([24], see [151, 175]). Due to the relative homogeneity of Kaibab forests, it seems unlikely that forest structure in and of itself would provide sufficient predictive cues to habitat quality, especially in view of extensive periodic variation in food abundance on the Kaibab [86].

Whether prey type and abundance cues were used by Kaibab goshawks was of course unknown. However, prey abundance on the Kaibab varied extensively over years [86, 176] and the simultaneous Kaibab-wide increases in number of goshawk territories that changed occupancy states from no breeders to breeding pairs (or breeding pairs to no breeders as food abundance increase (or decreased) indicated that prey abundance varied synchronous across the Kaibab [75]. Unfortunately, we were unable to determine if variation in prey abundance was equal in productive vs. less productive territories. Due to the 3–4 year periodicity in Kaibab prey abundance, food availability in a given year was unlikely to have been a reliable cue to future habitat quality.

Given the perceptual, temporal, and spatial constraints on young prospecting goshawks in relatively homogeneous habitat with temporally-variable food resources, it would not be surprising that prospectors used settled conspecifics in choosing their habitats. Settled conspecifics, especially if evidence of past or current successful breeding was available, provide cues to the potential quality of a habitat. Conspecific attraction also affords opportunities to detect and fill vacancies in established territories [154, 155, 177]. In fact, the importance of conspecifics to prospecting Kaibab goshawks was evidenced by the fact that 70% of new breeding recruits were replacements (turnovers) on territories where mates had either died or changed territories. Thus, rather than habitat quality *per se*, conspecific attraction could result in non-ideal habitat and/or mate choice whereby the most fit individuals did not necessarily settle in the best habitat or pair with the best mate. Conspecific attraction could result in consistent mate replacement that would, in and of itself, confer more breeding attempts and ultimately more productive and higher ranking territories.

In future research into the relative effects of individual quality and habitat quality on long-term territory-specific reproduction in goshawks, we anticipate investigating whether total fledgling production on a territory is better explained by differences among individual hawks, hawk pairs, or territories using both individual and territory identities as correlates of “territory quality” (*sensu* [147]). We then hope to investigate differences, if any, in over-story and understory species composition and 3-dimentional structure (from LiDAR) of forest vegetation at the territory-scale across the array of low to high fledgling-producing territories on the Kaibab Plateau. Finally, we caution that in future reproductively-based studies of habitat quality, it is essential to control for differences in individual quality (fitness) and to incorporate in such investigations as much as is known of the cues used, and decisions made, by individual birds during the process of habitat selection before drawing conclusions about habitat quality. This is especially true when the intent is to maintain population viability of a species by prioritizing high quality sites for conservation.

## Supporting information

S1 Fig Variance of lifespan and breeding lifespan in annual cohorts of 195 male and 250 female goshawks in Arizona, USA. Cohorts are collections of hawks (each individual represented by a row) first found breeding in a given year, 1991‒2010. Row cells completely filled with thick lines were unbanded individuals breeding on newly discovered territories, all had unknown prior breeding careers. Cells half-filled with thick lines represents replacements of prior breeders (i.e., turnovers) on territories under continuous monitoring; all replacements were assumed first-time breeders. Cells with thin lines leading to thick lines that fill a cell completely were known-age hawks (hatched in the year at the start of thin lines) whose first breeding (thick line) occurred in newly discovered territories. Based on 2-year-old minimum age at first breeding and infrequent first breeding by 3-year-olds, these hawks (*n* = 6) were assumed first-time breeders. Cells with thin lines leading to cells half filled with thick lines were known-age hawks that were replacements of prior breeders on continuously monitored territories. Length of thick lines represent observed breeding lifespans (first to last breeding attempt). Hawks still alive in 2009 may have had unknown future breeding careers.
(TIFF)

S2 Fig Scatterplot of body sizes of paired male and female goshawks and each pair’s total fledgling production from principle component analysis. (**A**) Body sizes in the year of pairing from the first principle component (PC; inset depicts variable loadings from the first PCs for males and females where body sizes were estimated from tarsom length, wing cord, tail length, and body mass) for 147 males and 151 females, and (**B**) heat map showing total fledgling produced over the duration of each pair bond (LR_pair_) from the first PC in 423 breeding attempts by northern goshawks in Arizona, USA, 1991–2010. Solid lines depict one-to-one relationships.
(TIFF)

S3 Fig Changes in LR associated with changes in mate mass following mate change and in ages at first breeding. Box plots of lifetime reproduction (LR) in relation to (**A**) direction of changed mate size for 89 females following breeding dispersal, (**B**) direction of changed mate size for 75 males following breeding dispersal, (**C**) standardized differences in age at first breeding for females, (**D**) standardized differences in age at first breeding for male northern goshawks in Arizona, USA.
(TIF)

S4 Fig Changes in LR associated with changes in number of breeding attempts and in lifespans. Box plots of individual lifetime reproduction (LR) in relation to (**A**) standardized number of breeding attempts by 89 females, (**B**) standardized number of breeding attempts by 75 males, (**C**) standardized lifespans of females, (**D**) standardized lifespans of male northern goshawks in Arizona, USA.
(TIF)

S5 Fig Changes in LR associated with changes in number of mates and in number of nest failures. Box plots of individual lifetime reproduction (LR) in relation to (**A**) standardized number of mates over the lifetime of 89 females, (**B**) standardized number of mates over the lifetime of 75 males, (**C**) standardized number of nest failures during the lifetime of females, (**D**) standardized number of nest failures during lifetime of male northern goshawks in Arizona, USA.
(TIF)

S6 Fig Changes in LR associated with the average rank of mates during a hawks’ lifetime and with changes in the density of breeders. Box plots of individual lifetime reproduction (LR) in relation to (**A**) standardized average mate rank (rank-ordering all males and females on LR where a ranking of 1 was the most productive) during lifetime of 89 females, (**B**) standardized average mate rank during lifetime of 75 males, (**C**) standardized average number of breeding pairs during reproductive years for females, (**D**) standardized average number of breeding pairs during reproductive years for male northern goshawks in Arizona, USA.
(TIF)

S7 Fig Changes in LR associated with changes in a territory’s rank and with changes in the degree of pair-specific differences in body sizes. Box plots of individual lifetime reproduction (LR) in relation to (**A**) standardized average territory rank (rank-ordered on final count of fledglings standardized by number of years a territory was monitored) over lifetime of 89 females, (**B**) standardized average territory rank over lifetime of 75 males, (**C**) standardized average percent mass of all mates (a measure of the degree of pair-specific size dimorphism, males smaller than females) during lifetime of females, (**D**) standardized average percent mass of all mates during lifetime of male northern goshawks in Arizona, USA.
(TIF)

S8 Fig Larger annual cohorts of fledglings produced more recruits. Number of banded northern goshawks recruits from each annual cohort of fledglings compared to the number of fledglings banded in that cohort in Arizona, USA.
(TIF)

S1 Table Correlations (*r*) for quantitative explanatory variables of lifetime reproduction (LR) for 65 male northern goshawks in Arizona, USA. (DOCX)

S2 Table Correlations (*r*) for quantitative explanatory variables of lifetime reproduction for 86 female northern goshawks in Arizona, USA. (DOCX)

S3 Table Candidate model set (*ΔAICc* < 2) for generalized linear models of individual and environmental influences on LR of 65 male goshawks including morphological data *tarsom*, *wingC*, *mass*, and *tailL* in Arizona, USA. All models include an intercept term. Degrees of freedom (*df*), log likelihood (*logLik*), *AICc*, delta *AICc*, and model weight (*weight*) are included.
(DOCX)

S4 Table Candidate model set (*ΔAICc* < 2) for generalized linear models of individual and environmental influences on LR of 86 female goshawks including morphological data *tarsom*, *wingC*, *mass*, and *tailL* in Arizona, USA. All models include an intercept term. Degrees of freedom (*df*), log likelihood (*logLik*), *AICc*, delta *AICc*, and model weight (*weight*) are included.
(DOCX)

S5 TableInfluence of individual and environmental effects on lifetime reproduction of goshawks.Relative slope parameter estimates from the generalized linear model candidate set without *tarsom*, *wingC*, *mass*, and *tailL* data. Presented is adjusted standard error ($\hat{S}E_{adj}$), relative importance, and *p*-values for the model terms for the influence of individual and environmental effects on lifetime reproduction of 75 male and 89 female northern goshawks in Arizona, USA.(DOCX)

S6 Table Breeding lifespans and number of lifetime mates of northern goshawks in Arizona, USA. (DOCX)

S7 Table Life expectancy of breeding northern goshawks in Arizona, USA. (DOCX)
