# Supplementary figures and images for: Factors affecting lifetime reproduction, long-term territory-specific reproduction, and estimation of habitat quality in northern goshawks
Source: PLoS One. 2019 May 22;14(5):e0215841. doi: 10.1371/journal.pone.0215841 (PMC6530838; doi:10.1371/journal.pone.0215841)

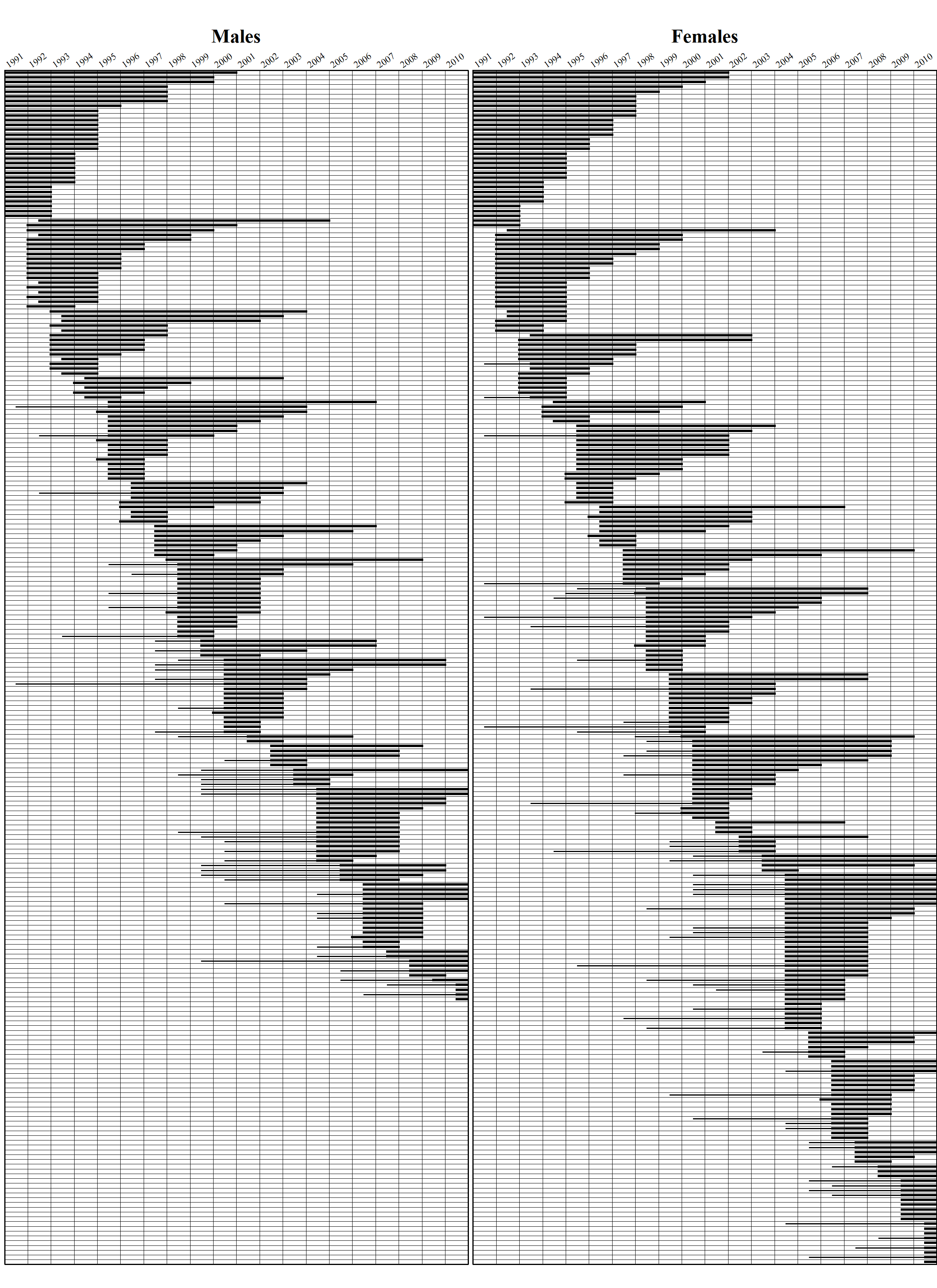

Supplement: S1 Fig — Cohorts are collections of hawks (each individual represented by a row) first found breeding in a given year, 1991‒2010. Row cells completely filled with thick lines were unbanded individuals breeding on newly discovered territories, all had unknown prior breeding careers. Cells half-filled with thick lines represents replacements of prior breeders (i.e., turnovers) on territories under continuous monitoring; all replacements were assumed first-time breeders. Cells with thin lines leading to thick lines that fill a cell completely were known-age hawks (hatched in the year at the start of thin lines) whose first breeding (thick line) occurred in newly discovered territories. Based on 2-year-old minimum age at first breeding and infrequent first breeding by 3-year-olds, these hawks (n = 6) were assumed first-time breeders. Cells with thin lines leading to cells half filled with thick lines were known-age hawks that were replacements of prior breeders on continuously monitored territories. Length of thick lines represent observed breeding lifespans (first to last breeding attempt). Hawks still alive in 2009 may have had unknown future breeding careers. (TIFF) [file pone.0215841.s001.tiff]

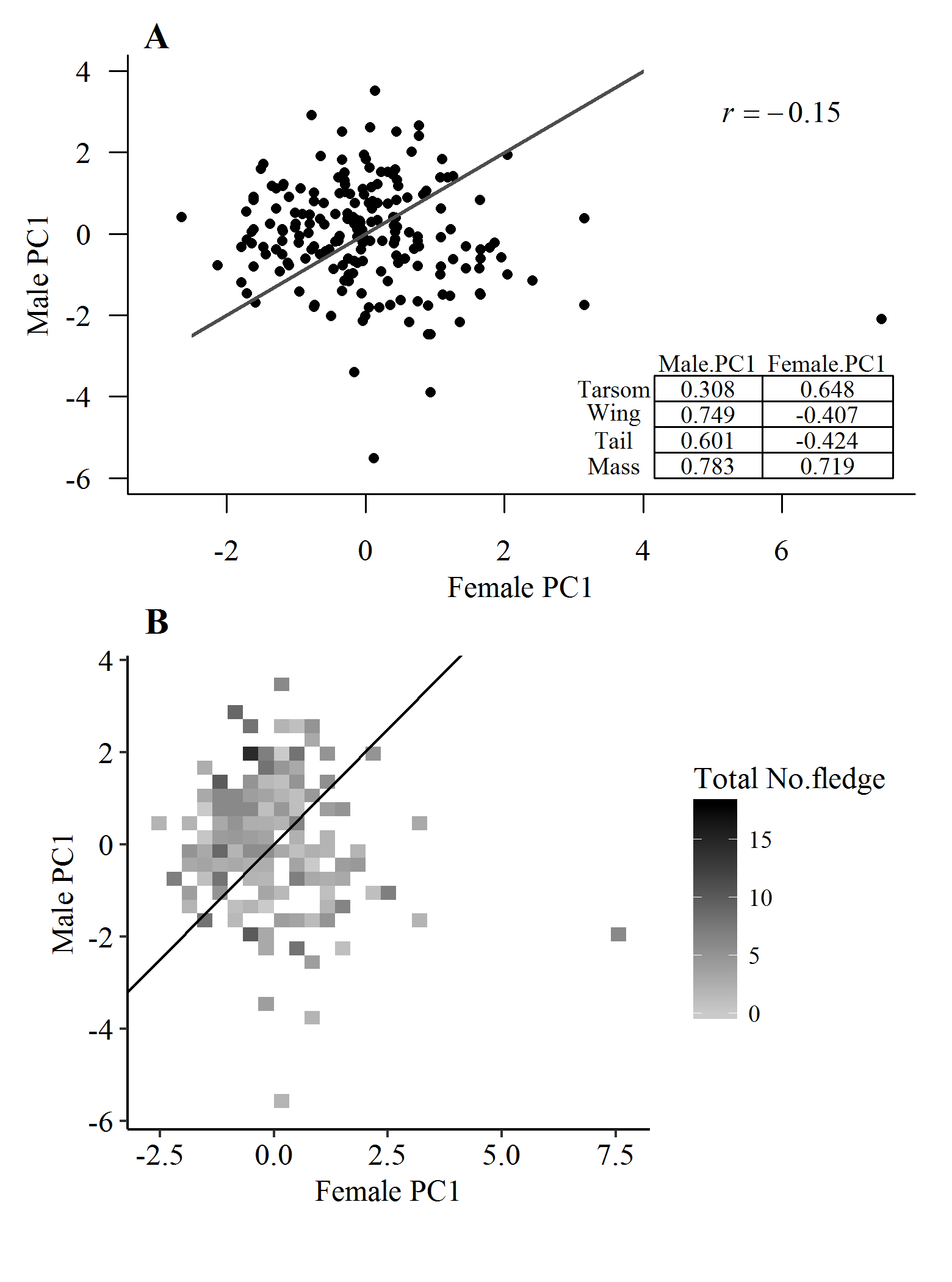

Supplement: S2 Fig — (A) Body sizes in the year of pairing from the first principle component (PC; inset depicts variable loadings from the first PCs for males and females where body sizes were estimated from tarsom length, wing cord, tail length, and body mass) for 147 males and 151 females, and (B) heat map showing total fledgling produced over the duration of each pair bond (LRpair) from the first PC in 423 breeding attempts by northern goshawks in Arizona, USA, 1991–2010. Solid lines depict one-to-one relationships. (TIFF) [file pone.0215841.s002.tiff]

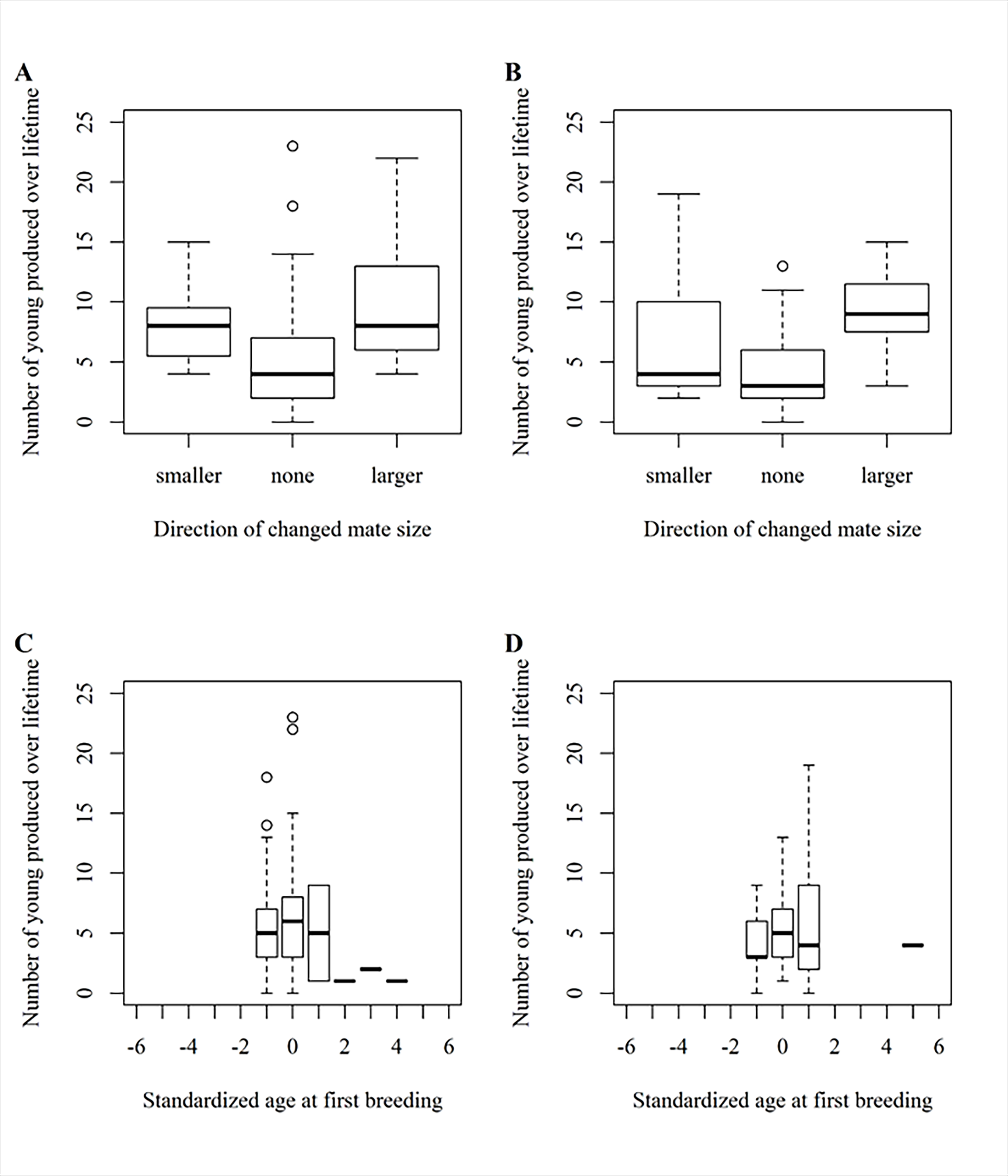

Supplement: S3 Fig — Box plots of lifetime reproduction (LR) in relation to (A) direction of changed mate size for 89 females following breeding dispersal, (B) direction of changed mate size for 75 males following breeding dispersal, (C) standardized differences in age at first breeding for females, (D) standardized differences in age at first breeding for male northern goshawks in Arizona, USA. (TIF) [file pone.0215841.s003.tif]

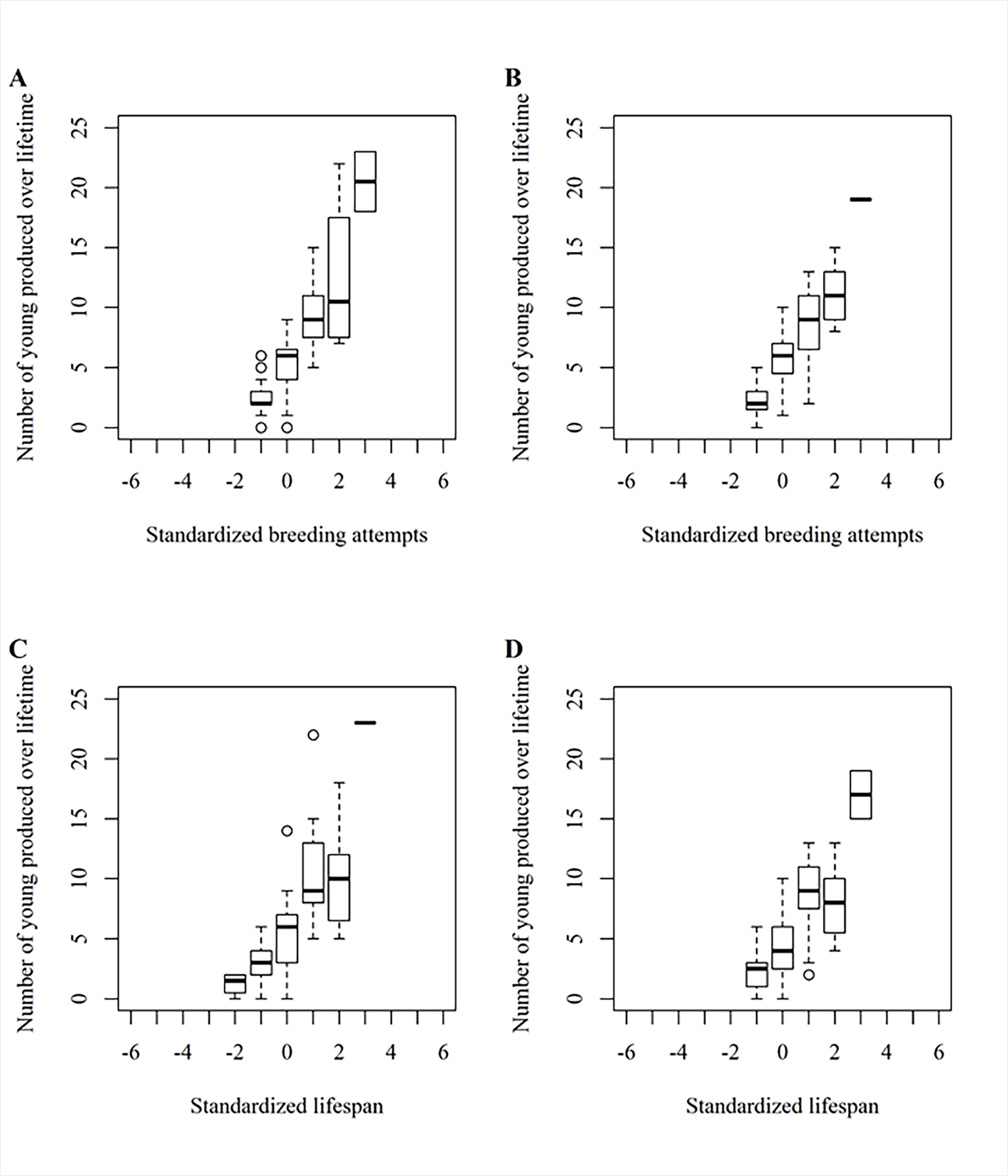

Supplement: S4 Fig — Box plots of individual lifetime reproduction (LR) in relation to (A) standardized number of breeding attempts by 89 females, (B) standardized number of breeding attempts by 75 males, (C) standardized lifespans of females, (D) standardized lifespans of male northern goshawks in Arizona, USA. (TIF) [file pone.0215841.s004.tif]

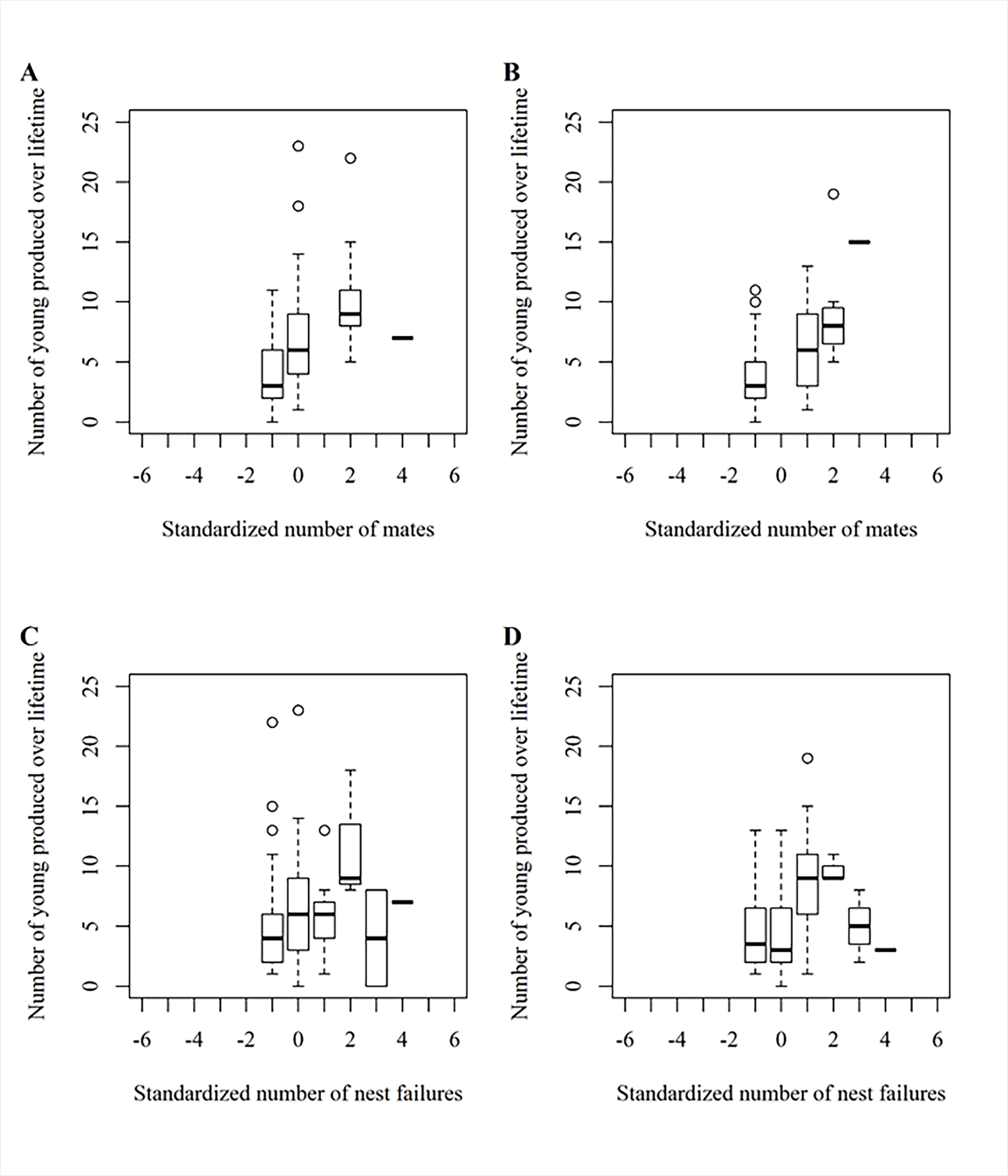

Supplement: S5 Fig — Box plots of individual lifetime reproduction (LR) in relation to (A) standardized number of mates over the lifetime of 89 females, (B) standardized number of mates over the lifetime of 75 males, (C) standardized number of nest failures during the lifetime of females, (D) standardized number of nest failures during lifetime of male northern goshawks in Arizona, USA. (TIF) [file pone.0215841.s005.tif]

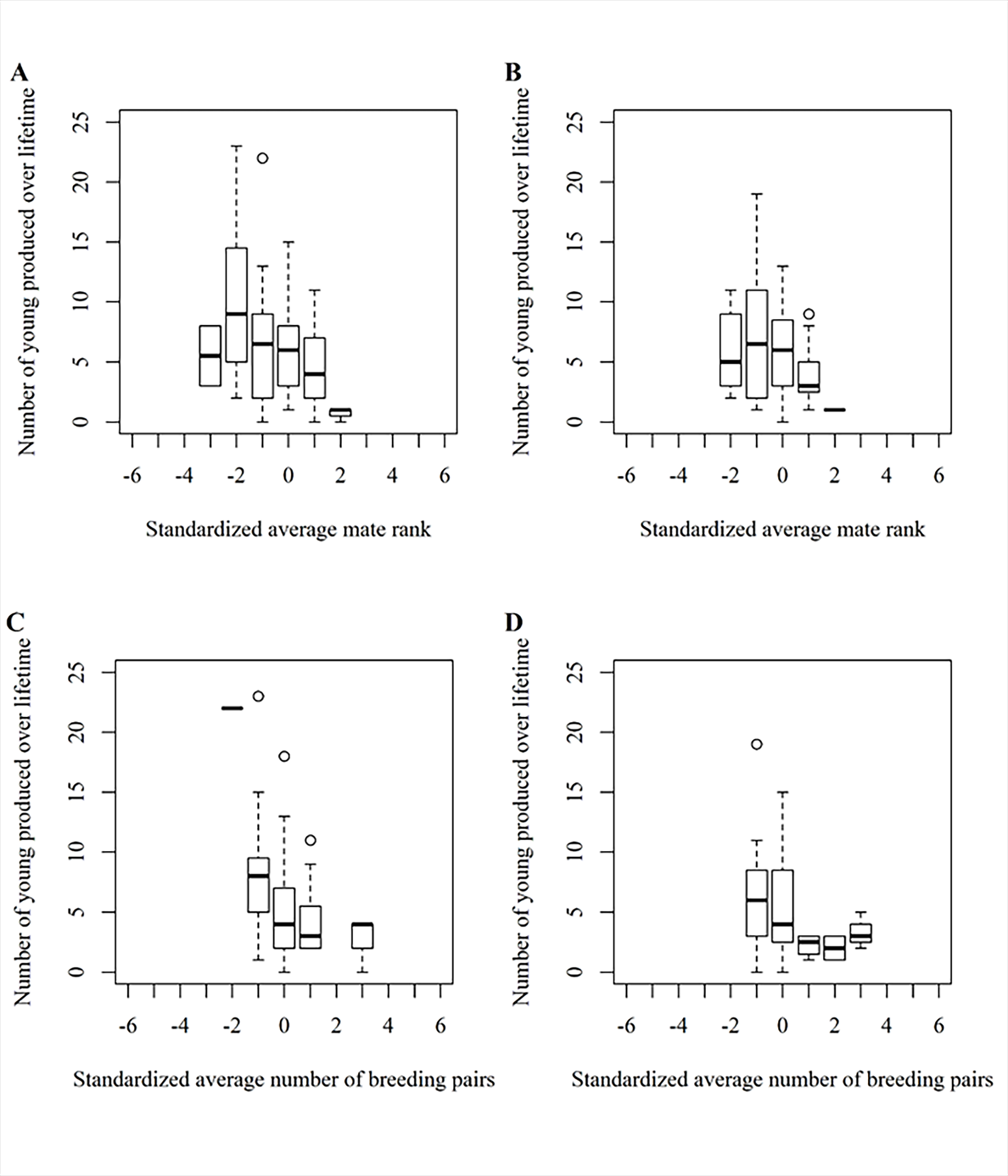

Supplement: S6 Fig — Box plots of individual lifetime reproduction (LR) in relation to (A) standardized average mate rank (rank-ordering all males and females on LR where a ranking of 1 was the most productive) during lifetime of 89 females, (B) standardized average mate rank during lifetime of 75 males, (C) standardized average number of breeding pairs during reproductive years for females, (D) standardized average number of breeding pairs during reproductive years for male northern goshawks in Arizona, USA. (TIF) [file pone.0215841.s006.tif]

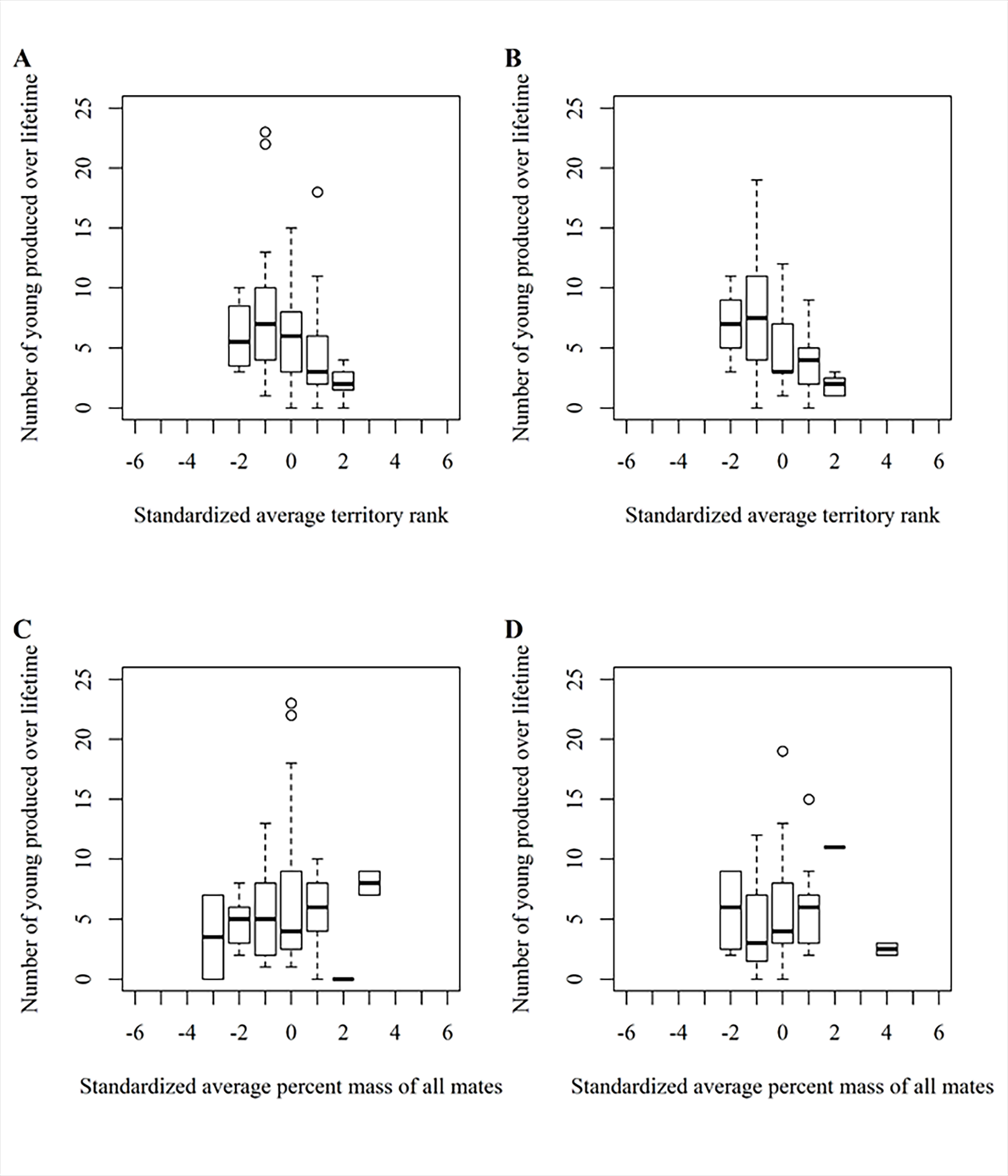

Supplement: S7 Fig — Box plots of individual lifetime reproduction (LR) in relation to (A) standardized average territory rank (rank-ordered on final count of fledglings standardized by number of years a territory was monitored) over lifetime of 89 females, (B) standardized average territory rank over lifetime of 75 males, (C) standardized average percent mass of all mates (a measure of the degree of pair-specific size dimorphism, males smaller than females) during lifetime of females, (D) standardized average percent mass of all mates during lifetime of male northern goshawks in Arizona, USA. (TIF) [file pone.0215841.s007.tif]

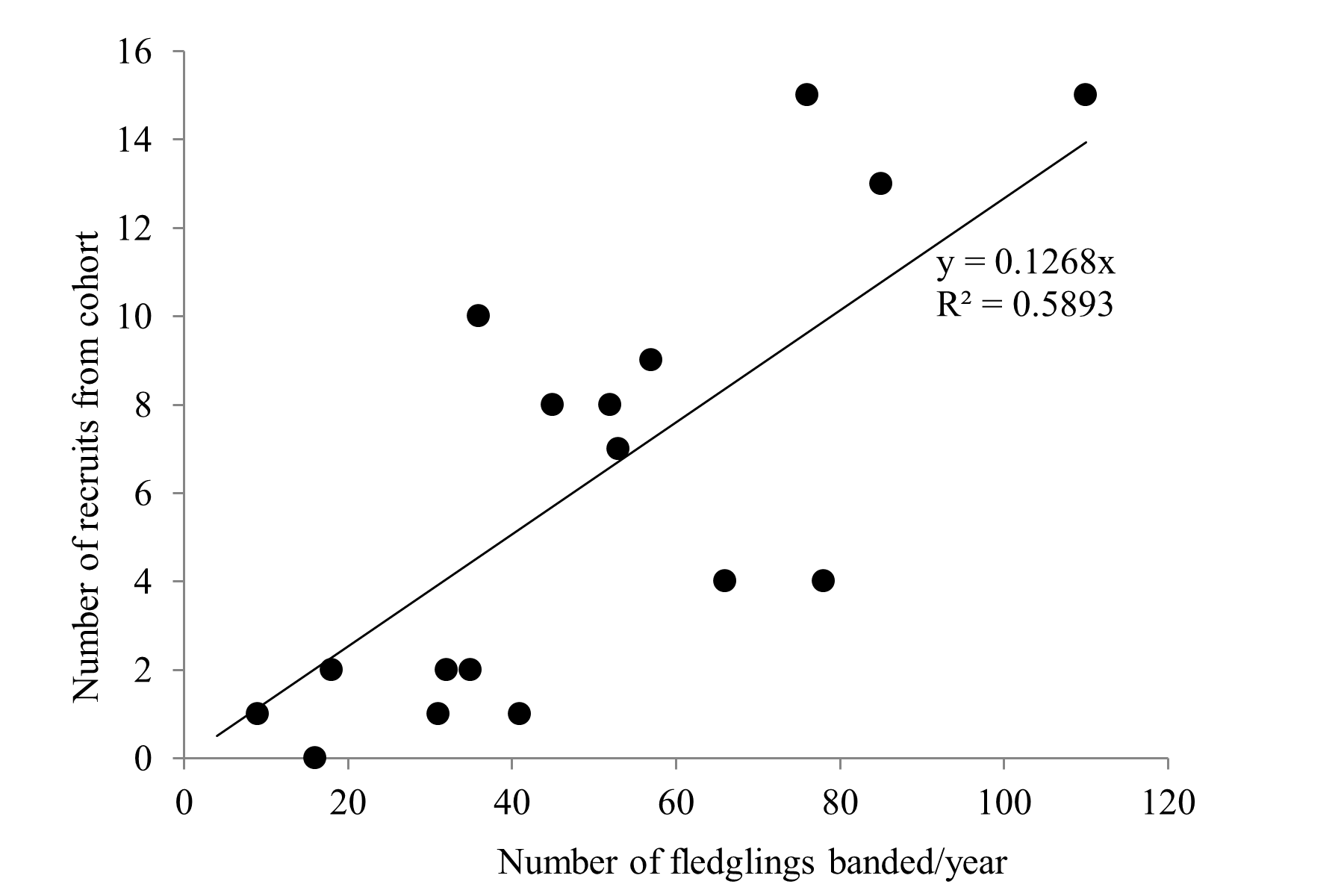

Supplement: S8 Fig — Number of banded northern goshawks recruits from each annual cohort of fledglings compared to the number of fledglings banded in that cohort in Arizona, USA. (TIF) [file pone.0215841.s008.tif]
