# Supplementary material for: Factors affecting lifetime reproduction, long-term territory-specific reproduction, and estimation of habitat quality in northern goshawks
Source: PLoS One. 2019 May 22;14(5):e0215841. doi: 10.1371/journal.pone.0215841 (PMC6530838; doi:10.1371/journal.pone.0215841)
Supplement: S1 Table — (DOCX) [file pone.0215841.s009.docx]

**S1 Table. This is the S1 Table Title**. **Correlations (*r*) for quantitative explanatory variables of lifetime reproduction (LR) for 65 male northern goshawks in Arizona, USA.**

|  | *lifespan* | *agefirst*  *breeding* | *breeding*  *attempts* | *Num*  *mates* | *mate*  *switch* | *nest*  *failures* | *tarsom* | *wingC* | *tail* | *mass* | *avgper*  *mass* | *avgbr*  *pairs* | *avgter*  *rank* | *avgmate*  *rank* |
| --- | --- | --- | --- | --- | --- | --- | --- | --- | --- | --- | --- | --- | --- | --- |
| *lifespan* | NA | 0.393 | 0.880 | 0.774 | 0.147 | 0.570 | -0.052 | 0.064 | 0.018 | 0.097 | 0.157 | -0.465 | -0.245 | -0.010 |
| *agefirstbreeding* |  | NA | 0.005 | 0.246 | 0.077 | -0.139 | -0.098 | -0.346 | -0.410 | -0.077 | 0.027 | -0.185 | 0.098 | 0.187 |
| *breedingattempts* |  |  | NA | 0.611 | 0.186 | 0.700 | 0.054 | 0.263 | 0.220 | 0.170 | 0.136 | -0.379 | -0.269 | -0.123 |
| *nummates* |  |  |  | NA | 0.112 | 0.451 | -0.039 | -0.005 | -0.127 | -0.052 | 0.047 | -0.346 | -0.188 | 0.193 |
| *mateswitch* |  |  |  |  | NA | 0.128 | 0.078 | 0.197 | 0.039 | 0.117 | -0.046 | 0.034 | -0.091 | 0.052 |
| *nestfailures* |  |  |  |  |  | NA | -0.013 | 0.268 | 0.268 | 0.222 | 0.106 | -0.259 | 0.056 | -0.005 |
| *tarsom* |  |  |  |  |  |  | NA | 0.076 | -0.096 | 0.174 | 0.010 | -0.125 | 0.001 | 0.183 |
| *wingC* |  |  |  |  |  |  |  | NA | 0.420 | 0.415 | 0.245 | 0.112 | -0.069 | -0.021 |
| *tail* |  |  |  |  |  |  |  |  | NA | 0.210 | 0.049 | 0.077 | -0.050 | -0.174 |
| *mass* |  |  |  |  |  |  |  |  |  | NA | 0.509 | 0.130 | -0.075 | 0.178 |
| *avgpermass* |  |  |  |  |  |  |  |  |  |  | NA | 0.034 | -0.165 | 0.001 |
| *avgbrpairs* |  |  |  |  |  |  |  |  |  |  |  | NA | 0.234 | 0.065 |
| *avgterrank* |  |  |  |  |  |  |  |  |  |  |  |  | NA | 0.273 |
| *avgmaterank* |  |  |  |  |  |  |  |  |  |  |  |  |  | NA |
