# Supplementary material for: Factors affecting lifetime reproduction, long-term territory-specific reproduction, and estimation of habitat quality in northern goshawks
Source: PLoS One. 2019 May 22;14(5):e0215841. doi: 10.1371/journal.pone.0215841 (PMC6530838; doi:10.1371/journal.pone.0215841)
Supplement: S2 Table — (DOCX) [file pone.0215841.s010.docx]

**S2 Table. This is the S2 Table Title**. **Correlations (*r*) for quantitative explanatory variables of lifetime reproduction for 86 female northern goshawks in Arizona, USA.**

|  | *lifespan* | *agefirst*  *breeding* | *breeding*  *attempts* | *Num*  *mates* | *mate*  *switch* | *nest*  *failures* | *tarsom* | *wingC* | *tail* | *mass* | *avgper*  *mass* | *avgbr*  *pairs* | *avgter*  *rank* | *avgmate*  *rank* |
| --- | --- | --- | --- | --- | --- | --- | --- | --- | --- | --- | --- | --- | --- | --- |
| *lifespan* | NA | 0.206 | 0.845 | 0.610 | -0.136 | 0.400 | 0.180 | -0.048 | 0.012 | 0.034 | -0.057 | -0.484 | -0.176 | -0.254 |
| *agefirstbreeding* |  | NA | -0.152 | -0.169 | -0.097 | -0.161 | 0.018 | 0.028 | 0.029 | -0.061 | -0.068 | -0.080 | -0.086 | -0.164 |
| *breedingattempts* |  |  | NA | 0.582 | 0.077 | 0.554 | 0.130 | -0.044 | -0.042 | -0.043 | -0.087 | -0.405 | -0.201 | -0.302 |
| *nummates* |  |  |  | NA | 0.071 | 0.262 | 0.238 | 0.000 | -0.101 | 0.001 | 0.013 | -0.388 | -0.106 | 0.155 |
| *mateswitch* |  |  |  |  | NA | 0.005 | 0.018 | 0.015 | 0.072 | -0.265 | -0.130 | -0.020 | -0.179 | -0.077 |
| *nestfailures* |  |  |  |  |  | NA | -0.054 | -0.060 | 0.058 | -0.079 | -0.277 | -0.211 | 0.112 | -0.035 |
| *tarsom* |  |  |  |  |  |  | NA | -0.079 | 0.053 | 0.181 | -0.011 | -0.363 | -0.103 | 0.147 |
| *wingC* |  |  |  |  |  |  |  | NA | 0.201 | 0.199 | 0.304 | 0.226 | -0.060 | -0.163 |
| *tail* |  |  |  |  |  |  |  |  | NA | 0.102 | 0.094 | 0.175 | 0.026 | -0.120 |
| *mass* |  |  |  |  |  |  |  |  |  | NA | 0.599 | -0.007 | 0.039 | 0.131 |
| *avgpermass* |  |  |  |  |  |  |  |  |  |  | NA | 0.039 | -0.004 | 0.000 |
| *avgbrpairs* |  |  |  |  |  |  |  |  |  |  |  | NA | 0.278 | 0.118 |
| *avgterrank* |  |  |  |  |  |  |  |  |  |  |  |  | NA | 0.438 |
| *avgmaterank* |  |  |  |  |  |  |  |  |  |  |  |  |  | NA |
