# Supplementary material for: Factors affecting lifetime reproduction, long-term territory-specific reproduction, and estimation of habitat quality in northern goshawks
Source: PLoS One. 2019 May 22;14(5):e0215841. doi: 10.1371/journal.pone.0215841 (PMC6530838; doi:10.1371/journal.pone.0215841)
Supplement: S3 Table — All models include an intercept term. Degrees of freedom (df), log likelihood (logLik), AICc, delta AICc, and model weight (weight) are included. (DOCX) [file pone.0215841.s011.docx]

**S3 Table. This is the S3 Table Title.** **Candidate model set (*ΔAICc* < 2) for generalized linear models of individual (including *tarsom*, *wing****C***, *mass*, and** *tailL***) and environmental influences on LR of 65 male goshawks in Arizona, USA.** All models include an intercept term. Degrees of freedom (*df*), log likelihood (*logLik*), *AICc*, delta *AICc*, and model weight (*weight*) are included.

| Model | *df* | *logLik* | *AICc* | *ΔAICc* | *weight* |
| --- | --- | --- | --- | --- | --- |
| *breedingattempts+mateswitch+nestfailures* | 5 | -123.990 | 258.997 | 0.000 | 0.171 |
| *avgterrank+breedingattempts+nestfailures* | 4 | -125.402 | 259.470 | 0.473 | 0.135 |
| *avgmaterank+breedingattempts+mateswitch+nestfailures* | 6 | -123.016 | 259.480 | 0.484 | 0.134 |
| *avgterrank+breedingattempts+mateswitch+nestfailures* | 6 | -123.055 | 259.558 | 0.561 | 0.129 |
| *avgbrpairs+breedingattempts+mateswitch+nestfailures* | 6 | -123.349 | 260.146 | 1.150 | 0.096 |
| *breedingattempts+nestfailures* | 3 | -127.169 | 260.731 | 1.734 | 0.072 |
| *avgterrank+breedingattempts+nestfailures+nummates* | 5 | -124.898 | 260.813 | 1.816 | 0.069 |
| *avgmaterank+avgterrank+breedingattempts+mateswitch+nestfailures* | 7 | -122.445 | 260.855 | 1.859 | 0.067 |
| *avgbrpairs+avgmaterank+breedingattempts+mateswitch+nestfailures* | 7 | -122.486 | 260.937 | 1.940 | 0.065 |
| *avgpermass+breedingattempts+mateswitch+nestfailures* | 6 | -123.765 | 260.978 | 1.981 | 0.063 |
