# Supplementary material for: Factors affecting lifetime reproduction, long-term territory-specific reproduction, and estimation of habitat quality in northern goshawks
Source: PLoS One. 2019 May 22;14(5):e0215841. doi: 10.1371/journal.pone.0215841 (PMC6530838; doi:10.1371/journal.pone.0215841)
Supplement: S4 Table — All models include an intercept term. Degrees of freedom (df), log likelihood (logLik), AICc, delta AICc, and model weight (weight) are included. (DOCX) [file pone.0215841.s012.docx]

**S4 Table. This is the S4 Table Title.** **Candidate model set (*ΔAICc* < 2) for generalized linear models of individual (including *tarsom*, *wing****C***, *mass*, and *tail****L***) and environmental influences on LR of 86 female goshawks in Arizona, USA.** All models include an intercept term. Degrees of freedom (*df*), log likelihood (*logLik*), *AICc*, delta *AICc*, and model weight (*weight*) are included.

| Model | *df* | *logLik* | *AICc* | *ΔAICc* | *weight* |
| --- | --- | --- | --- | --- | --- |
| *agefirstbreeding+avgterrank+breedingattempts+mateswitch+nestfailures* | 7 | -165.415 | 346.265 | 0.000 | 0.197 |
| *agefirstbreeding+avgterrank+breedingattempts+nestfailures* | 5 | -167.916 | 346.583 | 0.318 | 0.168 |
| *agefirstbreeding+breedingattempts+mateswitch+nestfailures* | 6 | -166.885 | 346.833 | 0.568 | 0.149 |
| *agefirstbreeding+breedingattempts+nestfailures* | 4 | -169.223 | 346.939 | 0.674 | 0.141 |
| *agefirstbreeding+avgterrank+breedingattempts+nestfailures+nummates* | 6 | -167.221 | 347.504 | 1.239 | 0.106 |
| *agefirstbreeding+breedingattempts+nestfailures+nummates* | 5 | -168.529 | 347.808 | 1.543 | 0.091 |
| *agefirstbreeding+avgterrank+ breedingattempts+mateswitch+nestfailures+tailL* | 8 | -165.179 | 348.228 | 1.963 | 0.074 |
| *agefirstbreeding+avgterrank+breedingattempts+mass+nestfailures* | 6 | -167.596 | 348.255 | 1.990 | 0.073 |
