# Supplementary material for: Factors affecting lifetime reproduction, long-term territory-specific reproduction, and estimation of habitat quality in northern goshawks
Source: PLoS One. 2019 May 22;14(5):e0215841. doi: 10.1371/journal.pone.0215841 (PMC6530838; doi:10.1371/journal.pone.0215841)
Supplement: S5 Table — Relative slope parameter estimates from the generalized linear model candidate set without tarsom, wingC, mass, and tailL data. Presented is adjusted standard error (S^Eadj), relative importance, and p-values for the model terms for the influence of individual and environmental effects on lifetime reproduction of 75 male and 89 female northern goshawks in Arizona, USA. (DOCX) [file pone.0215841.s013.docx]

## **S5 Table. This is the Table S5 Title. Influence of individual and environmental effects on lifetime reproduction of goshawks.** Slope parameter estimates from the generalized linear model candidate set without *tarsom*, *wingC*, *mass*, and *tailL* data. Presented are adjusted standard error ($\hat{S}E_{adj}$), relative importance, and *p*-values for the model terms for the influence of individual and environmental effects on lifetime reproduction of 75 male and 89 female northern goshawks in Arizona, USA.

|  | **Males** | | | **Females** | | |
| --- | --- | --- | --- | --- | --- | --- |
| **Model term** | **Estimate**  **(±SE_adj_)** | **Relative importance** | ***P*^1^** | **Estimate (±SE_adj_)** | **Relative importance** | ***P*^1^** |
| ***Intercept*** | 1.425(0.129) |  | <0.001*** | 1.789(0.160) |  | <0.001*** |
| ***Avgbrpairs*** | -0.014(0.044) | 0.283 | 0.750 | -0.010(0.035) | 0.272 | 0.779 |
| ***Agefirstbreeding*** | 0.000(0.030) | 0.232 | 0.991 | -0.134(0.068) | 0.915 | 0.048* |
| ***Avgmaterank*** | -0.034(0.058) | 0.419 | 0.556 | -0.018(0.045) | 0.327 | 0.694 |
| ***Avgpermass*** | 0.002(0.029) | 0.236 | 0.934 | 0.004(0.027) | 0.246 | 0.879 |
| ***Avgterrank*** | -0.062(0.076) | 0.543 | 0.421 | -0.060(0.065) | 0.607 | 0.356 |
| ***Breedingattempts*** | 0.653(0.075) | 1.000 | <0.001*** | 0.612(0.055) | 1.000 | <0.001*** |
| ***Nummates*** | 0.018(0.045) | 0.315 | 0.693 | 0.008(0.037) | 0.281 | 0.822 |
| ***Mate switch, no change in mate*** | 0.041(0.127) | 0.603 | 0.749 | -0.257(0.190) | 0.742 | 0.177 |
| ***Mate switch, larger mate***^2^ | 0.207(0.210) | - | 0.324 | -0.175(0.175) | - | 0.316 |
| ***Nestfailures*** | -0.343(0.080) | 1.000 | <0.001*** | -0.252(0.055) | 1.000 | <0.001*** |

^1^Significance level: *0.05, ** 0.01, *** 0.001.

^2^Mate switch is a categorical variable and had multiple factor levels (hence the multiple lines within the table). Relative importance only tracks the explanatory variable, not the levels.
