# Supplementary material for: Factors affecting lifetime reproduction, long-term territory-specific reproduction, and estimation of habitat quality in northern goshawks
Source: PLoS One. 2019 May 22;14(5):e0215841. doi: 10.1371/journal.pone.0215841 (PMC6530838; doi:10.1371/journal.pone.0215841)
Supplement: S6 Table — (DOCX) [file pone.0215841.s014.docx]

**S6 Table. This is the S6 Table Title. Breeding lifespans and number of lifetime mates of northern goshawks in Arizona, USA.**

|  |  | Number of hawks by breeding lifespan | |
| --- | --- | --- | --- |
| Breeding lifespan of hawk (yrs) | Number of lifetime mates | Males | Females |
| 1 | 1 | 61 | 39 |
| 2 | 1 | 47 | 40 |
| 2 | 2 | 2 | 5 |
| 3 | 1 | 30 | 33 |
| 3 | 2 | 9 | 9 |
| 3 | 3 | 1 |  |
| 4 | 1 | 17 | 10 |
| 4 | 2 | 8 | 8 |
| 4 | 3 | 1 | 1 |
| 5 | 1 | 11 | 1 |
| 5 | 2 | 5 | 11 |
| 5 | 3 | 2 | 1 |
| 5 | 4 | 1 |  |
| 6 | 1 | 6 | 3 |
| 6 | 2 | 11 | 7 |
| 6 | 3 | 3 | 1 |
| 7 | 1 | 2 |  |
| 7 | 2 | 8 | 6 |
| 7 | 3 | 2 | 1 |
| 7 | 4 |  | 1 |
| 8 | 1 | 3 | 4 |
| 8 | 2 | 5 | 5 |
| 8 | 3 | 2 | 1 |
| 9 | 2 | 3 | 3 |
| 9 | 3 | 3 | 2 |
| 9 | 5 | 1 |  |
| 10 | 2 | 3 |  |
| 10 | 5 |  | 1 |
| 11 | 1 | 1 |  |
| 11 | 4 |  | 1 |
| 12 | 2 | 1 |  |
| 12 | 3 |  | 1 |
