# Supplementary material for: Factors affecting lifetime reproduction, long-term territory-specific reproduction, and estimation of habitat quality in northern goshawks
Source: PLoS One. 2019 May 22;14(5):e0215841. doi: 10.1371/journal.pone.0215841 (PMC6530838; doi:10.1371/journal.pone.0215841)
Supplement: S7 Table — (DOCX) [file pone.0215841.s015.docx]

**S7 Table. This is the S7 Table Title. Life expectancy of breeding northern goshawks in Arizona, USA.**

| Age (yr) | Apparent survival^1^ | Mortality rate^2^ | Crude death rate^3^ | Proportional death rate^4^ | Survivorship^5^ | Midpoint survivorship^6^ | Age categories left^7^ | Life expectancy |
| --- | --- | --- | --- | --- | --- | --- | --- | --- |
| 2 | 0.74 | 0.26 | 10,000 | 2,600 | 1 | 1.37 | 5.43 | 5.43 |
| 3 | 0.76 | 0.24 | 7,400 | 1,776 | 0.74 | 1.02 | 4.06 | 5.49 |
| 4 | 0.77 | 0.23 | 5,624 | 1,293 | 0.56 | 0.78 | 3.04 | 5.40 |
| 5 | 0.78 | 0.22 | 4,331 | 953 | 0.43 | 0.60 | 2.26 | 5.22 |
| 6 | 0.77 | 0.23 | 3,378 | 777 | 0.34 | 0.47 | 1.66 | 4.91 |
| 7 | 0.76 | 0.24 | 2,601 | 624 | 0.26 | 0.36 | 1.19 | 4.58 |
| 8 | 0.75 | 0.25 | 1,977 | 494 | 0.20 | 0.27 | 0.83 | 4.20 |
| 9 | 0.72 | 0.28 | 1,483 | 415 | 0.15 | 0.20 | 0.56 | 3.77 |
| 10 | 0.69 | 0.31 | 1,068 | 331 | 0.11 | 0.14 | 0.36 | 3.35 |
| 11 | 0.64 | 0.36 | 737 | 265 | 0.07 | 0.10 | 0.21 | 2.90 |
| 12 | 0.58 | 0.42 | 472 | 198 | 0.05 | 0.06 | 0.12 | 2.47 |
| 13 | 0.51 | 0.49 | 274 | 134 | 0.03 | 0.03 | 0.06 | 2.03 |
| 14 | 0.43 | 0.57 | 140 | 92 | 0.01 | 0.02 | 0.02 | 1.52 |
| 15 | 0.34 | 0.66 | 48 | 31 | 0.00 | 0.01 | 0.00 | 1.01 |
| 16 | 0.26 | 0.74 | 17 | 12 | 0.00 | 0.00 | 0.00 | 0.00 |
| 17 | 0.18 | 0.82 | 5 | 4. | 0.00 | 0.00 | 0.00 | 0.00 |
| 18 | 0.12 | 0.88 | 1 | 1. | 0.00 | 0.00 | 0.00 | 0.00 |

^1^Apparent survival of breeding goshawks from [75].

^2^1 - survival.

^3^Number of individuals who die within age group.

^4^Proportion of individuals who die within age group.

^5^Proportion of individuals who survive to the beginning of the next age group.

^6^ Proportion of individuals who survive to the midpoint of the next age group.

^7^Number of age groups remaining until death for individuals surviving to the next age group.
